# Copper(II) Complexes of Selected Acylhydrazones as Potential Biological Agents

**DOI:** 10.3390/ijms262210980

**Published:** 2025-11-13

**Authors:** Izabela Czyżewska, Liliana Mazur, Robert Mroczka, Anna Biernasiuk, Anna Hordyjewska, Łukasz Popiołek

**Affiliations:** 1 Chair and Department of Organic Chemistry, Faculty of Pharmacy, Medical University of Lublin, 4A Chodźki Street, 20-093 Lublin, Poland; 50460@umlub.edu.pl; 2 Institute of Chemical Sciences, Faculty of Chemistry, Maria Curie-Skłodowska University, Maria Curie-Skłodowska Sq. 2, 20-031 Lublin, Poland; liliana.mazur@mail.umcs.pl; 3 Laboratory of X-ray Optics, Centre for Interdisciplinary Research, The John Paul II Catholic University of Lublin, 1J Konstantynów Street, 20-708 Lublin, Poland; robert.mroczka@kul.pl; 4 Chair and Department of Pharmaceutical Microbiology, Faculty of Pharmacy, Medical University of Lublin, 1 Chodźki Street, 20-093 Lublin, Poland; anna.biernasiuk@umlub.pl; 5 Department of Medicinal Chemistry, Faculty of Medical Sciences, Medical University of Lublin, 4A Chodźki Street, 20-093 Lublin, Poland; anna.hordyjewska@umlub.pl

**Keywords:** copper(II) complexes, acylhydrazones, crystal structure, antibacterial activity, anticancer activity, cytotoxicity, halogen bond

## Abstract

In the current research a series of new copper(II) complexes with novel acylhydrazone ligands were synthesized and their antibacterial and anticancer activities were determined. The complexes were characterized by molecular spectroscopy (FT-IR and UV-Vis) and conductivity measurements. Additionally, their structure was confirmed by single-crystal X-ray analysis. The crystallographic data revealed that all compounds are mononuclear Cu(II) species. The Cu(II) ion is four-coordinated by the ONO donor set from mono-deprotonated hydrazone ligand and one Cl^¯^ anion, forming distorted square-planar geometry. The biological studies revealed that the compounds exhibit high antimicrobial activity, especially against Gram-positive bacteria, in some cases greater than the reference substances, and better activity than free ligands. The tested complexes possessed the lowest MIC and MBC values towards *Staphylococcus epidermidis* ATCC 12228 and *Micrococcus luteus* ATCC 10240. Furthermore, they showed no toxicity towards normal cell lines.

## 1. Introduction

The problem of drug resistance is currently a major challenge for modern medicine. Bacteria have developed various mechanisms to defend themselves against antibiotics [1]. The World Health Organization has recognized drug resistance as one of the three greatest threats to public health of the 21st century. Infections with drug-resistant bacteria are associated with an increase in mortality compared with infections caused by antibiotic-sensitive bacteria. They also cause an increase in medical costs, which constitute a heavy burden on the economy [2]. Hence, there is an urgent need to search for new antibacterial agents.

Acylhydrazones have been studied and reported in the scientific literature for many years [3,4,5]. They are formed mainly by the condensation reaction of aldehydes or ketones with carboxylic acid hydrazides [4,5,6]. They are interesting mainly due to their biological activities, such as their antibacterial [6,7], anticancer [8], antifungal [9] and antituberculosis properties [10]. On the pharmaceutical market there are medicines which contain acylhydrazone moiety like nitrofurazone, furazolidone or nitrofurantoin [4,5,6]. They are also very useful in chemical organic synthesis and as ligands in coordination chemistry due to their chelating ability, mainly in reaction with transition metals [11,12].

Metal complexes can possess biological activity because of the presence of both the ligand and the metal atom. In many cases they display positive synergism and possess better activity than organic ligands themselves [12,13]. Complexation of acylhydrazones usually occurs via an imine nitrogen atom, an amide oxygen and, additionally, a suitable donor oxygen or nitrogen atom located in the appropriate position for chelation, forming three-coordination complexes [14,15,16]. Complexes of acylhydrazones with copper(II) as well as with nickel(II), cobalt(II) and zinc(II) are of great interest [6,17,18,19,20,21,22].

The scientific literature describes positive aspects of using complexation reactions to enhance the biological activity of compounds, e.g., their antimicrobial, anticancer, anti-inflammatory activity. Complexation increases lipophilicity, allowing better drug penetration through the bacterial cell membrane [23]. Metal complexes can show biological activity because of the presence of both the ligand and the metal atom. In many cases they display positive synergism and manifest better activity than the organic ligands themselves. The mechanism of antibacterial action of complexes can also be explained by the disruption of the activity of the bacterial enzyme DNA gyrase, necessary for DNA replication and transcription [23,24].

Among them, copper complexes receive the most attention. In particular, copper(II) has a significant impact on antibacterial activity. This ion has redox reactivity, converting from Cu^2+^ to Cu^+^, thus generating reactive oxygen species (ROS) that can damage bacterial DNA, proteins, and cell membranes. Moreover, copper has the ability to bind to enzymes such as cytochrome C through the SH group in bacterial cells [25].

Extensive studies have been conducted on improving the antibacterial activity of antibiotics through reactions with transition metals. The hypothesis proposed in this study was confirmed, which represents an interesting direction of research into discovering new compounds with significant antimicrobial potential [24].

Considering the above-described facts, in particular concerning the biological activity of acylhydrazones and their metal complexes, five new copper(II) complexes of acylhydrazones of 2- or 3-iodobenzoic acid were synthesized. The synthesis of the ligands and their biological activity were described in our previous paper [6]. The aim of this work was to determine antimicrobial and anticancer activity of the newly obtained complexes and to assess how biological activity changes with the modification of the chemical structure. Another goal was to establish the crystal structure of the compounds and assess their stability in the solid state and solution. An important aspect of this work is to determine whether the activity of the obtained complexes will be higher than the activity of the ligands themselves, as indicated by the literature data.

## 2. Results and Discussion

### 2.1. Chemistry

#### 2.1.1. Synthesis and Chemical Characterization of Obtained Complexes

Copper(II) complexes **1**–**5** were prepared with good yield (70–85%) in the reaction of appropriate acylhydrazone ligand **HL^1^**–**HL^5^** [6] dissolved in hot ethanol with ethanol solution of CuCl_2_, both mixed at a 1:1 molar ratio. All studied complexes **1**–**5** were obtained as green crystalline solids being air stable. The obtained compounds are hardly soluble in water and in most polar organic solvents. Moderate solubility in dimethylsufoxide (DMSO) allowed us to conduct biological assays in polar solutions by adding the corresponding compound in DMSO.

The comparison of the FT-IR spectra of the complexes **1**–**5** (Figures S5–S9, Supplementary Materials) with those of the free ligands **HL^1^**–**HL^5^** [6] evidenced coordination of hydrazones to the Cu(II) ion via enolate oxygen, azomethine nitrogen and phenolate oxygen. In the FT-IR spectra of the complexes **1**–**5**, their characteristic ν(C=O) and ν(O–H) vibrational modes disappear, whereas a very strong band at 1377–1369 cm^−1^ appears, indicating that the hydrazone ligands are deprotonated and coordinated to the metal center through the enol tautomer. The characteristic ν(N–N) stretching vibrations observed in the spectra of neutral acylhydrazones around 1040 cm^−1^ undergo a shift to higher wavenumbers (1064–1046 cm^−1^) due to an increase in the double bond character, this being further proof of the ligand coordination via azomethine nitrogen. Moreover, there are newly formed bands in the range of 1522–1499 cm^−1^ because of the partly double C^1^ = N^1^ bond creation, which further confirms ligands enolization. In the FT-IR spectrum of complex **4**, a very broad band with the maximum at 3364 cm^−1^ confirms the presence of ethanol molecules in the crystal.

#### 2.1.2. Conductivity Measurements

**Figure 1:**
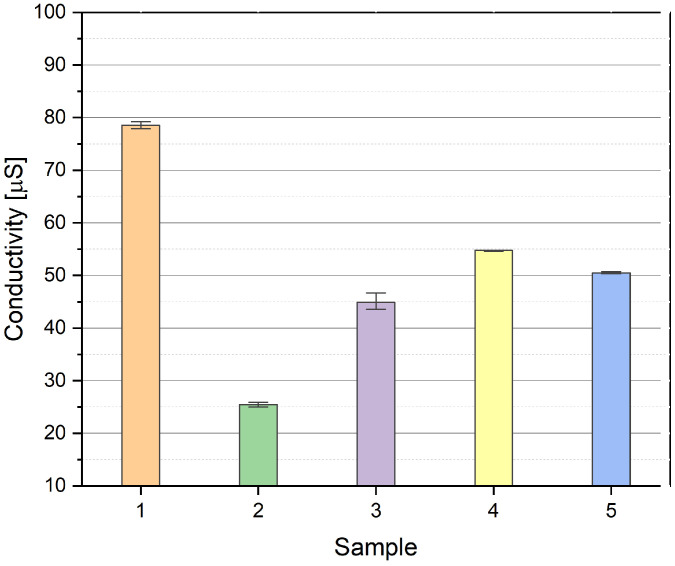
The conductivity (μS/m) of complexes **1**–**5** in DMSO solution.

The conductivity of the examined complexes **1**–**5** is varied (Figure 1). The greatest conductivity (around 80 µS/m) is observed for complex **1** and the lowest for **2**. It is determined by the substitution of two chlorides by bromides in the organic ligand in complex **2**. Complexes **3**–**5** demonstrate a roughly similar conductivity, being equal to around 50 µS/m. The conductivity measurements confirm that the studied complexes **1**–**5** are stable, and the anions are coordinated to the metal ions in the test solution medium.

#### 2.1.3. UV-Vis Spectroscopy

**Figure 2:**
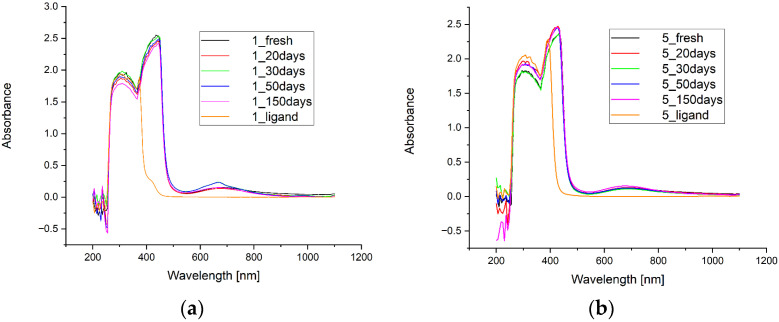
UV-Vis spectra of copper(II) complexes **1** (**a**) and **5** (**b**) in DMSO, recorded for fresh solutions as well as after 20, 30, 50 and 150 days, in comparison with the spectra of the respective hydrazone ligands.

The electronic spectra of complexes **1**–**5** in DMSO (Figure 2 and Figure S4, Supplementary Materials) demonstrate characteristic absorption bands for the Cu(II) complexes.

In the spectra of the starting ligands **HL^1^**–**HL^5^**, the absorption bands with maximum around 310 nm are characteristic for all acylhydrazones and drop rapidly at 384 nm for **HL^1^** and **HL^2^** and around 415 nm for **HL^3^**–**HL^5^**, respectively. The highest intensity of the absorption bands for complexes **1** and **2** are observed at 440 nm, while, for **3**, **4** and **5**, the maximum of the band is blue-shifted to 425 nm (hypsochromic effect). The absorption bands around 430 nm are determined by π → π*, while d-d bands around 650 nm are contributed to from four excitation state transitions [26].

The greatest and perfect stability (evaluated for fresh solutions and after 20, 30, 50 and 150 days) is observed for complex **2**, while other complexes demonstrate very high stability as well. Small differences observed for UV-VIS spectra can arise from instrument issues or lower homogeneity of solutions in comparison to complex **2**. The transparency of **HL^3^** ligand solution was not complete unlike with other solutions of the ligands and determines greater absorbance for the region 450–1100 nm.

#### 2.1.4. X-Ray Crystallography

**Figure 3:**
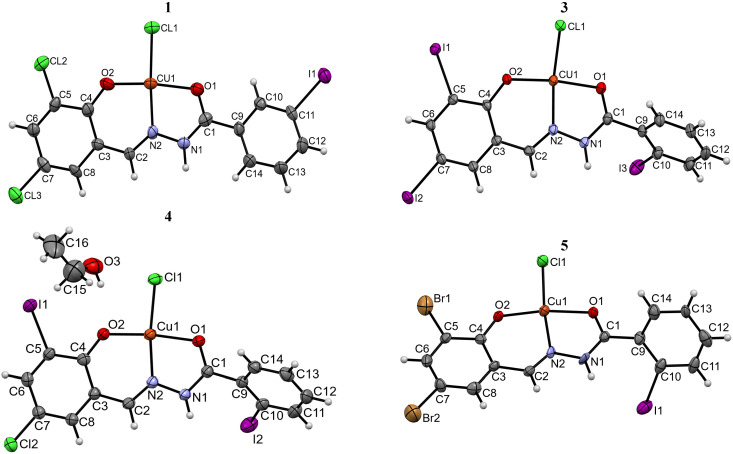
Perspective view of the asymmetric unit in Cu(II) complexes **1**, **3**, **4** and **5** with an atom-numbering scheme. Thermal ellipsoids are drawn at the 30% probability level.

The molecular plots for complexes **1**, **3**, **4** and **5** with the atom-labeling schemes are presented in Figure 3. The selected bond lengths, angles and torsion angles are given in Tables S1–S3 in Supplementary Materials.

The single-crystal X-ray diffraction analysis revealed that all studied complexes **1**–**5** are mononuclear species of the general formula [Cu(L)Cl]. Compounds **1**, **2**, **3** and **5** exist in their unsolvated forms, whereas **4** is a stoichiometric (2:1) ethanol solvate. Complexes **3**–**5** crystallize in the centrosymmetric space groups *P*2_1_/*c*, *I*2/*a*, whereas compounds **1** and **2** in the non-centrosymmetric space group *Pc* (Table 1 and Section S1 in Supplementary Materials), in each case with one molecule of the complex in the asymmetric part of the unit cell. Significant similarity of the unit cell parameters of crystals **1**, **2, 3** and **5** suggests their isostructurality.

The central Cu(II) ion is tetra-coordinated in all complexes. The aroylhydrazone ligand is coordinated as a mono-anion via the hydrazide-O1, imine-N2 and phenoxo-O2 atoms, playing the role of tridentate chelating agent. The coordination sphere of the metal center is completed by one chloride anion. The analysis of the bond lengths and bond angles around the Cu(II) ion (Tables S1 and S2 in Supplementary Materials) reveals slightly distorted geometry. As a result, the Cu(II) ion is shifted by 0.018 Å (**5**)–0.111 Å (**1**) from the best plane of O1, O2, N2, Cl1 atoms. The coordination of the Cu(II) ion to the deprotonated ligand results in the formation of five- and six-membered chelate rings, with the dihedral angle between the best planes of the respective atoms in the range of 2.11° (**4**)–4.14° (**1**). Moreover, the latter ring is almost coplanar with the phenoxy system. The shortest interatomic distances in the coordination environment are those to the deprotonated hydroxyl O2 atom [1.85(2)–1.904(4) Å], whereas the longest ones are Cu1–Cl1 bonds, being in the range of 2.210(5)–2.248(7) Å (Table S1 in Supplementary Materials). The Cu1–O1 [1.977(4)–1.990(11) Å] and Cu1–N2 [1.929(4)–1.943(15) Å] distances are comparable with the values observed in other Cu(II) complexes with closely related aroylhydrazone ligands [CSD Refcodes: AMUWUC [27], BOMXEG10 [28], JICVID [29], LARNEZ [30], TOMGIN [31], UMERIP, UMESAI [32], ZOTCAO [33] and TAMDUK [34]]. The bond angles around the Cu(II) center (Table S2 in Supplementary Materials) do not deviate by more than 10° from 90° or 180°, which confirms the distorted flat-square geometry of the coordination environment. The ligands adopt the *trans* configuration around the imine C=N bond with *syn* conformation of the hydrazone function. It is noteworthy that the same geometry is observed in the crystals of pure hydrazones [6]. The central hydrazone–amide unit is almost flat, as evidenced by the torsion angles around the C1–N1, N1–N2 and N2–C2 bonds (Table S3 in Supplementary Materials). Comparing the respective torsion angle values in crystals of free ligands [6] and those in complexes **1**–**5**, the most significant rotation is observed around the amide C1–N1 bond. It is noteworthy that the hydrazone ligands **HL^1^**–**HL^5^** in the complexes are not flat—the dihedral angles between the best planes of phenyl rings are in the range of 17° (**1**)–65° (**3**). The essential conformational dissimilarity between the neutral molecules and the ligands in the complexes is due to the twist of the C9 << C14 phenyl ring. The conformational change can be described by the O1–C1–C9–C10 torsion angle values, which are −131.6(7)°, −135.2(6)° and −131.1(18)°, respectively, in complexes **3**, **4** and **5** and −58.8(12)°, 39.6(11)° and 65.1(10)° in the crystals of starting hydrazones [6]. Furthermore, coordination with the metal center imposes a slight modification of the bond angles around the C1 and C9 atoms. In particular, there is an increase in the N1–C1–C9 angle from 114.0(7)° (**HL^3^**) − 115.5(7)° (**HL^4^**) in free ligands [6] to 117.4(6)° (**3**) − 121.7(15)° (**5**) in the complexes.

The crystal structures of the studied complexes **1**–**5** are stabilized by combination of strong hydrazone···chloride N1–H1···Cl1 hydrogen bonds (Table S4 in Supplementary Materials) supported by weak C–H∙∙∙Hal (Hal = Cl, Br, I) and Hal···Hal [35,36] interactions. More details concerning the intermolecular interaction patterns and crystal packing features can be found in Supplementary Materials.

### 2.2. Microbiology

The antibacterial activity of the new complexes **1**–**5** was tested towards reference Gram-positive (nine strains) and Gram-negative (five strains) bacteria. Additionally, antifungal effect against yeasts belonging to *Candida* spp. (six strains) was investigated. Data regarding the results of microbiological tests are presented in Table 2. The heatmap of antimicrobial activity of synthesized complexes is presented in Supplementary Materials (Table S7). The activity data of the tested complexes and positive controls expressed as MIC [µg/mL] of triplicate in vitro screening with mean and standard deviation (+/− SD) against the reference strains of bacteria and fungi is presented in Supplementary Materials (Table S8).

The research results revealed excellent antimicrobial activity of the tested complexes, especially towards Gram-positive bacteria (Table 2). The range of minimal inhibitory concentrations (MIC) which inhibited growth of reference strains of *Staphylococcus* spp. *Enterococcus faecalis* ATCC 29212, *Micrococcus luteus* ATCC 10240 and *Bacillus* spp. was from 0.98 to 15.62 µg/mL. This antibacterial effect was very strong (MIC < 10 µg/mL) or strong (MIC = 10–25 µg/mL). It was observed that *M. luteus* ATCC 10240 was the most sensitive to all tested complexes **1**–**5** (MIC = 0.98–7.81 µg/mL). Additionally, the MBC values ranged from 3.91 to 62.5 µg/mL and were the same or 2- or 4-fold higher than the MIC (MBC/MIC = 1–4), indicating the bactericidal effect of tested complexes **1**–**5** (Table 2).

In the case of reference Gram-negative bacteria from *Enterobacterales* family and *Pseudomonas aeruginosa* ATCC 9027, no activity was found. Only the rods belonging to *Klebsiella pneumoniae* ATCC 13883, *Proteus mirabilis* ATCC 12453 and *Salmonella typhimurium* ATCC 14028 were susceptible to complexes **1** and/or **2** at MIC = 1000 µg/mL and MBC > 1000 µg/mL (Table 2).

The obtained data presented in Table 2 indicated also some antifungal bioactivity of tested complexes **1**–**5** towards yeasts. The minimal concentrations of these substances that inhibited the growth of *Candida* spp. or killed them ranged from 125 to 1000 µg/mL and from 250 to > 1000 µg/mL, respectively. Almost all complexes showed moderate anticandidal bioactivity with MIC = 250–500 µg/mL, MFC = 250–1000 µg/mL and fungicidal effect. Some of them indicated good fungicidal activity, namely complex **1** towards one strain of *Candida albicans* ATCC 2091 and *Candida glabrata* ATCC 90030 (MIC = 125 µg/mL; MFC = 250–500 µg/mL) and complex **4** against *C. glabrata* ATCC 90030 (MIC = 125 µg/mL; MFC = 250 µg/mL). Only in the case of complex **3** was mild activity (MIC = 1000 µg/mL; MFC ≥ 1000 µg/mL) towards *C. glabrata* ATCC 90030, *C. auris* CDC 311903 and *C. krusei* ATCC 14243 observed (Table 2).

In the case of activity against Gram-positive bacteria, it can be noticed that complexes **1**–**5** showed very strong activity with MIC < 10 µg/mL and strong activity with MIC was equal to 10–25 µg/mL. Complexes **1**–**5** showed worse activity towards fungi where the activity was considered good and the MIC was in the range of 26–125 µg/mL and moderate bioactivity where the MIC was in the range of 126–500 µg/mL. The weakest activity can be observed for Gram-negative bacteria. Complexes in this group of bacteria showed mild bioactivity and the MIC was in the range of 501–1000 µg/mL or they showed no activity (Table 2).

Complex **1** showed the best activity against Gram-positive bacteria. In the case of all tested bacteria except *Staphylococcus aureus* ATCC 29213, it showed very strong activity in the range MIC of 0.98–7.81 µg/mL. It showed strong activity (MIC = 15.62 µg/mL) against *Staphylococcus aureus* ATCC 29213. Towards *Micrococcus luteus* ATCC 10240, the activity of this compound was the same as for cefuroxime (MIC = 0.98 µg/mL), whereas, against *Bacillus subtilis* ATCC 6633, the activity (MIC = 3.91 µg/mL) was the most beneficial, at the same level as for nitrofurantoin (MIC = 3.91 µg/mL), i.e., four times greater than that of cefuroxime (MIC = 15.62 µg/mL) and sixteen times greater than that of ampicillin (MIC = 62.5 µg/mL) (Table 2).

Complex **4** also showed very strong activity against *Staphylococcus aureus* ATCC 6538 and *Bacillus subtilis* ATCC 6633 (MIC = 15.62 µg/mL). Moreover, antibacterial activity of this complex against *Staphylococcus aureus* ATCC 25923 (MIC = 3.91 µg/mL) was four times higher than for nitrofurantoin (MIC = 15.62 µg/mL). The same activity as for nitrofurantoin (MIC = 7.81 µg/mL) was found for compound **4** against *Staphylococcus aureus* ATCC 43300, *Staphylococcus epidermidis* ATCC 12228 and *Bacillus cereus* ATCC 10876. Additionally, towards *Bacillus cereus* ATCC 10876, the MIC values for this complex were four times lower (MIC = 7.81 µg/mL) than for cefuroxime (MIC = 31.25 µg/mL) (Table 2).

Complexes **2** and **5** showed similar antibacterial activity. For most of the strains of Gram-positive bacteria, they showed very strong activity (MIC = 0.98–7.81 µg/mL) and, for the others, strong activity (MIC = 15.62 µg/mL) (Table 2).

Complex **2** showed a very low MIC value (0.98 µg/mL) against *Micrococcus luteus* ATCC 10240. Towards *Bacillus subtilis* ATCC 6633, it displayed activity two times greater (MIC = 7.81 µg/mL) than that of cefuroxime (MIC = 15.62 µg/mL) and, against *Bacillus cereus* ATCC 10876, four times greater (MIC = 7.81 µg/mL) than cefuroxime (MIC = 31.25 µg/mL). Complex **2** against *Staphylococcus aureus* ATCC 25923 showed the same antibacterial activity as nitrofurantoin (MIC = 15.62 µg/mL) (Table 2).

The MIC value of complex **5** against *Staphylococcus aureus* ATCC 25923 (MIC = 7.81 µg/mL) was two times lower than for nitrofurantoin (MIC = 15.62 µg/mL). The activity of this complex towards *Micrococcus luteus* ATCC 10240 (MIC = 7.81 µg/mL) was eight times higher than the activity of nitrofurantoin (MIC = 62.5 µg/mL). Additionally, the MIC values of this compound were four time lower (MIC = 7.81 µg/mL) than for cefuroxime (MIC = 31.25 µg/mL) (Table 2) towards *Bacillus cereus* ATCC 10876.

Complex **3** showed 16 times higher activity (MIC = 3.91 µg/mL) than nitrofurantoin (MIC = 62.5 µg/mL) against *Micrococcus luteus* ATCC 10240 and activity (MIC = 3.91 µg/mL) equal to nitrofurantoin (MIC = 3.91 µg/mL) towards *Staphylococcus epidermidis* ATCC 12228.

All tested complexes **1**–**5** showed very low MIC values ranging from 0.98 to 7.81 µg/mL against *Micrococcus luteus* ATCC 10240. The impressive results were also shown by the tested compounds against *Staphylococcus epidermidis* ATCC 12228 (MIC = 1.95–7.81 µg/mL) (Table 2).

It is noteworthy that all complexes **1**–**5** showed better antimicrobial properties in comparison with the ligands **HL^1^**–**HL^5^**, whose activity was reported by our research group earlier [6].

Complex **1** showed greater activity than the starting ligand for most Gram-positive bacterial strains. The best improvement in activity, up to 16-fold, was observed for *Micrococcus luteus* ATCC 10240. Moreover, ligand **HL^1^** showed no activity against fungi and Gram-negative bacteria, while complex **1** showed weak activity against all tested fungi and selected Gram-negative bacterial strains such as *Klebsiella pneumoniae* ATCC 13883, *Proteus mirabilis* ATCC 12453 and *Salmonella typhimurium* ATCC 14028 [6].

In the case of complex **2**, antibacterial activity was equal or better for Gram-positive bacteria than for ligand **HL^2^**. An improved activity in complex **2** can be seen against bacteria *Staphylococcus epidermidis* ATCC 12228, *Micrococcus luteus* ATCC 10240 and *Bacillus cereus* ATCC 10876. The ligand **HL^2^** showed no activity against Gram-negative bacteria, but the complex possessed weak activity against some of them [6].

In the case of complexes **3** and **4**, a significant improvement in activity against Gram-positive bacteria was observed. Ligands **HL^3^** and **HL^4^** showed weak activity against these bacterial strains, and the complexes had activity higher than or equal to nitrofurantoin.

On the other hand, the **HL^5^** ligand did not show any effect on the tested microorganisms, whereas its Cu(II) complex (**5**) had high antibacterial activity, similar to or even better than nitrofurantoin [6].

In summary, the improved activity can be explained by the fact that the complexation reaction increases the lipophilicity of the entire compound, allowing it to better penetrate through bacterial biological membrane and, consequently, to exert a stronger effect on it. Copper, which in itself has antibacterial properties, is also important. Comparing the results of the studies on ligands and complexes, it can be clearly concluded that the complexation reaction and the metal used improve the antimicrobial activity of a compound.

### 2.3. Cytotoxicity Study

During 24 h as well as 48 h culture of the L929 line it was shown that the tested complexes **1**–**5** did not cause a significant decrease in cell viability. Cell viability remained at 72–106% (Table S9 and Figures S10–S21 in Supplementary Materials). Similar results were also obtained for A549 cells at both incubation times. In this case, cell viability was maintained at 65–104% (Table S11 and Figures S34–S45 in Supplementary Materials). In turn, the T47D and HeLa cell lines were slightly more sensitive to the tested complexes **1**–**5**—especially at their higher concentrations (Tables S10 and S12 and Figures S22–S33 and S46–S57 in Supplementary Materials).

In the case of the T47D cell line, complexes **4** and **5** caused a decrease in viability by about 45% at concentrations from 100 to 200 µM during both 24 h and 48 h cultures. The most sensitive line to the tested complexes was the cervical cancer line (HeLa). In this case, a decrease in viability was observed for complex **1** at a concentration of 100–200 µM and for complexes **3** and **4** at concentrations of 50–100 µM during 24 and 48 h of culture (Tables S10 and S12 and Figures S22–S33 and S46–S57 in Supplementary Materials).

Since none of the tested compounds caused a 50% reduction in the viability of any cell line, the IC_50_ dose could not be determined in this study. However, the studies conducted by Alam and Lee [37] showed the efficacy of the new salicylic acid hydrazone derivatives against the A549 line at doses ranging from 2.35 µM to over 100 µM, depending on the compound. Nevertheless, we did not observe such antitumor activity in the case of our complexes [37]. The anticancer properties of new hydrazide-hydrazones of tetracaine were also observed by Han and İmamoğlu [38] but on other cell lines—human HCC cell line HepG2 and colon cancer cell line Colo-205 [38]. Similarly, Horchani et al. [39] showed the antiproliferative activity of synthetic pyrazolo-pyrimidinones combined with hydrazide-hydrazones against MCF-7 breast cancer cells [39]. Our complexes **1**–**5** did not show antiproliferative properties, perhaps for several reasons:

(1) High molecular weight, which makes it difficult for them to penetrate the cell membrane into the cell;
(2) Different chemical structure compared with other compounds, e.g., a different type of substituents in the rings and general number of rings;
(3) The lack of sensitivity of the tested cell lines to complexes **1**–**5**, perhaps other cancer lines should be tested.

Nevertheless, the lack of cytotoxicity towards normal cells qualifies them as complexes whose action can be considered at other levels of medical research.

## 3. Materials and Methods

### 3.1. Chemistry

The chemicals and solvents were purchased from commercial sources Sigma-Aldrich Co. (St. Louis, MO, USA), Merck Co. (Darmstadt, Germany), Polish Chemical Reagents (Gliwice, Poland)) and used without further purification. The IR spectra of the obtained complexes and the samples after recrystallization experiments were recorded in the range 4000–600 cm^−1^ on a Nicolet 6700 FT-IR spectrophotometer in ATR mode. Elemental analysis was performed on EuroEA Elemental Analyser. Conductivity was determined by multifunction meter CX-701 (Elmetron, Zabrze, Poland) equipped with CP-60 electrode immersed in complex solutions at concentrations of 2 mg/mL in DMSO. The UV-VIS measurements were carried out on the HALO DB 20S UV-VIS Spectrometer (Dynamica, Geneve, Switzerland) in the spectral range 200–1100 nm, 400 nm/min, path length: 10 mm. Ligands **HL^1^**–**HL^5^** and complexes **1**–**5** were dissolved in DMSO (1 mg/mL) and thoroughly mixed every time using a laboratory vortex mixer before UV-VIS measurements. UV-VIS spectra were collected for fresh solutions and after 20, 30, 50 and 150 days. Between measurements of 150 days, the solutions were exposed to natural sunlight.

### 3.2. Synthesis and Chemical Characterization

**Scheme 1:**
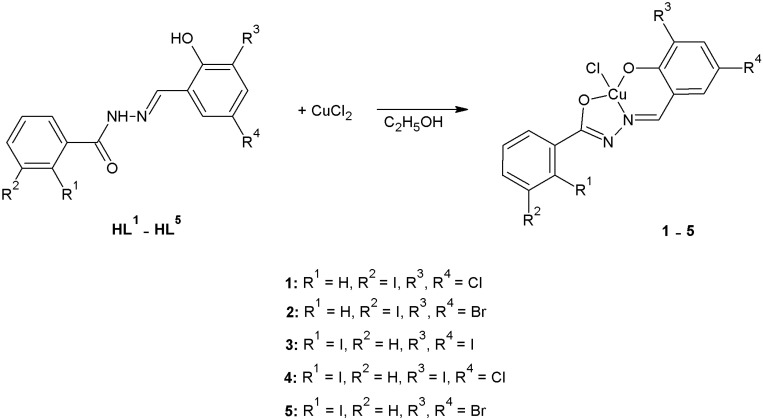
Synthesis of acylhydrazone complexes.

The organic ligands (**HL^1^**–**HL^5^**) used for the synthesis of complexes **1**–**5** were obtained following the general procedure reported in our previous paper [6]. All complexes **1**–**5** were prepared using the same method as described. In general, a stoichiometric amount of appropriate acylhydrazone (1.0 mmol) was dissolved in 20 mL of 99.8% ethanol by stirring at 60 °C for 15 min. A stoichiometric amount of CuCl_2_ (170.48 mg, 1.0 mmol) was dissolved in 20 mL of 99.8% ethanol and was added dropwise to cool solution of acylhydrazone **HL^1^**–**HL^5^** upon mixing. The green solution was further stirred at room temperature for 15 min and filtered. The final solution was left to evaporate for a few days to form crystals used for X-ray diffraction measurements and spectral characterization. The crystals were isolated, washed with dry ethanol and dried in the air at room temperature (Scheme 1). The data from UV-Vis and IR spectra is presented in the form of tables in Supplementary Materials (Table S5 and S6).

**Complex 1:** Colour: green crystals; Elemental Analysis (C_14_H_8_N_2_O_2_ICl_3_Cu): Calculated: C, 31.55; H, 1.51; N, 5.25%, Found: C, 31.87; H, 1.48; N, 5.24%. IR (cm^−1^): 3162 (NH), 3067, 3046 (CH, arom.), 1611 (C=N), 1592, 1582, 1542, 1469 (C_ar_=C_ar_), 1515 (C–N, amide), 1064 (N–N);

**Complex 2:** Colour: green crystals; Elemental Analysis (C_14_H_8_N_2_O_2_IBr_2_ClCu): Calculated: C, 27.03; H, 1.29; N, 4.50%, Found: C, 26.95; H, 1.27; N, 4.43%. IR (cm^−1^): 3166 (NH), 3058, 3013 (CH, arom.), 1608 (C=N), 1589, 1566, 1540, 1469 (C_ar_=C_ar_), 1502 (C–N, amide), 1063 (N–N);

**Complex 3:** Colour: green crystals; Elemental Analysis (C_14_H_8_N_2_O_2_I_3_ClCu): Calculated: C, 23.49; H, 1.12; N, 3.91%, Found: C, 23.81; H, 1.13; N, 3.93%. IR (cm^−1^): 3151 (NH), 3046, 3022 (CH, arom.), 1612 (C=N), 1574, 1558, 1545, 1486 (C_ar_=C_ar_), 1517 (C–N, amide), 1046 (N–N);

**Complex 4:** Colour: green crystals; Elemental Analysis (C_15_H_11_N_2_O_2.5_I_2_Cl_2_Cu): Calculated: C, 27.82; H, 1.71; N, 4.33%, Found: C, 27.74; H, 1.69; N, 4.35%. IR (cm^−1^): 3364 (OH), 3166 (NH), 3044 (CH, arom.), 1607 (C=N), 1584, 1570, 1521, 1489 (C_ar_=C_ar_), 1521 (C–N, amide), 1055 (N–N);

**Complex 5:** Colour: green crystals; Elemental Analysis (C_14_H_8_N_2_O_2_IBr_2_ClCu): Calculated: C, 27.03; H, 1.29; N, 4.50%, Found: C, 27.45; H, 1.25; N, 4.52%. IR (cm^−1^): 3153 (NH), 3058, 3026 (CH, arom.), 1614 (C=N), 1586, 1570, 1560, 1545, 1468 (C_ar_=C_ar_), 1499 (C–N, amide), 1047 (N–N).

### 3.3. Crystal Structure Determination

Single-crystal X-ray diffraction measurements for crystals **1**–**4** were performed using the Rigaku XtaLAB MM7HFMR diffractometer equipped with the “quarter-chi single” goniometer, CuKα radiation and the Pilatus 200K detector. For crystal **5** the data collection measurements were conducted applying the Agilent Technologies Xcalibur CCD diffractometer with graphite-monochromated MoKα radiation. The CrysAlisPro software (versions: 1.171.39.46, 1.171.40.45a, 1.171.41.123a) [40] was used for data collection, cell refinement, data reduction and analysis in all experiments. The multi-scan absorption correction was applied. The structures were solved using the direct methods implemented in the SHELXT [41] and were refined with the SHELXL-18/3 program [42]. All non-hydrogen atoms were refined with anisotropic displacement parameters using full-matrix least-square techniques on *F*^2^. The atoms of the disordered solvent molecule in **4**, located around the 2-fold axis, were refined with s.o.f. = 0.5. Hydrogen atoms from the amide group in crystals **1** and **3** as well as that from the hydroxyl group of ethanol molecule in structure **4** were located in the different Fourier maps and, where possible, they were refined using the isotropic displacement parameters. All remaining H atoms were placed geometrically and refined using the “riding model” with *U*_iso_(H) = 1.2–1.5 *U*_eq_(C, N). Mercury 2023.3.0 [43] was used to prepare molecular plots. The final data collection parameters and refinement statistics for structures **1**, **3**, **4** and **5** are summarized in Table 1. The crystal data for complex **2** are given in Supplementary Materials.

### 3.4. Microbiology

#### In Vitro Antimicrobial Assay

The examined complexes **1**–**5** were screened in vitro for antibacterial and antifungal activities using the broth microdilution method according to European Committee on Antimicrobial Susceptibility Testing (EUCAST) [44] and Clinical and Laboratory Standards Institute guidelines [45] against a panel of reference strains of microorganisms. Detailed procedures are presented in Supplementary Materials and reported in earlier articles [44,45,46,47,48].

### 3.5. Cytotoxicity Study

#### 3.5.1. Description of Cell Lines

The HeLa cell line (ATCC^®^ CCL-2™) (cervical cancer) and L929 cell line (clone NCTC 929, ATCC^®^ CCL-1™) (mouse fibroblasts) were from the ATCC collection. Both cell lines were suspended in Eagle Minimal Essential Medium supplemented with antibiotics (1% 100 U/L penicillin and 100 mg/mL streptomycin) and 10% and 5% FBS, respectively. The A549 cell line (ECACC 86012804) (non-small cell lung cancer) and T47D cell line (ECACC 85102201) (breast cancer) were from ECACC collection. Cells of these lines were suspended in Dulbecco’s Minimal Essential Medium supplemented with 2 mM glutamine, antibiotics (100 mg/mL streptomycin and 1% 100 U/L penicillin) and 10% fetal bovine serum. All cell lines were routinely cultured in 75 cm^2^ tissue culture flasks and stored in an incubator (humidified atmosphere of 5% CO_2_ at 37 °C). These cell lines were tested for mycoplasma contamination using microbiological tests.

#### 3.5.2. MTT Analysis

The cell viability of the tested complexes **1**–**5** was assessed using the MTT assay. For this purpose, cells of the appropriate cell lines were seeded in an amount of 100 μL/well on plates. Then, after the 24 h needed for cell attachment to the plate, the cells were treated with different concentrations of compounds (range 10–100 μM). After another 24 and 48 h of cells’ incubation with the tested complexes **1**–**5**, cytotoxicity was determined using the MTT assay. For this purpose, 10 μL of MTT at a concentration of 5 mg/mL was added to each well. The plates were incubated at 37 °C for another 4 h. Then, the medium was removed, and the resulting purple formazan precipitates were dissolved in 100 μL of DMSO. Absorbance (*Abs*) was read at 570 nm using a BioTek model EPOCH ELISA plate reader. All experiments were performed in triplicate. Absorbance values were within the linear range of the Lambert–Beer law. 

The percentage cell viability was calculated with respect to the solvent control as follows:

$$\%\;Cell\;viability=\;\frac{{Abs}_{test}}{{Abs}_{solvent\;control}}\times 100$$

## 4. Conclusions

This article presents a method known in the scientific literature for the synthesis of acylhydrazone complexes, but the described complexes have not yet been reported and tested for biological activity. In this research the synthesis and characterization of the new mononuclear copper(II) complexes derived from acylhydrazones of 2- or 3-iodobenzoic acid are reported. The reaction of respective **HL^1^**–**HL^5^** ligands with an equivalent amount of CuCl_2_ in anhydrous ethanol yields to complexes [M(L^1−5^)Cl], where the monobasic hydrazone ligand acts in the O,N,O-tridentate fashion.

The molecular and crystal structure of the newly obtained copper(II) complexes **1**–**5** was studied by single-crystal X-ray analysis and IR spectroscopy. Additionally, chemical stability of the complexes in the solvent used for biological assays was confirmed using UV-Vis spectrophotometry and conductivity measurements. The crystallographic studies showed tetracoordinated complexes with distorted square-planar geometry around the metal ion. The crystal structure of the complexes is mainly stabilized by strong N–H∙∙∙Cl^−^ hydrogen bonds supported by weak C–H∙∙∙Hal intermolecular interactions; however, halogen bonds are also important in the overall stabilization of those structures. Hence, it can be assumed that halogen bonds can be important in receptor–ligand interactions in the cells.

The results of our research revealed significant antibacterial activity of the complexes **1**–**5** towards reference Gram-positive bacterial strains, with very strong or strong bactericidal effect (MIC = 0.98–15.62 µg/mL; MBC/MIC = 1–4). Additionally, moderate activity against yeasts from *Candida* spp. and mild or no activity towards Gram-negative rods was observed. The most active complex **1** turned out to be the compound containing two Cl substituents in its structure. Impressive antimicrobial activity was also demonstrated by compound **4**, which had I and Cl atoms in its structure. The remaining compounds also possessed high antimicrobial activity. On the basis of the obtained results, it can be concluded that substitution with halogen atoms had high impact on antibacterial activity. In the comparison with the ligands described in the previous publication [6], it can be stated that introduction of Cu(II) ion improved antimicrobial activity.

In addition to the above, the lack of toxicity to normal lines also proves that described complexes **1**–**5** may be considered as antibacterial agents in the future.

## Figures and Tables

**Table 1:** Crystal data and final refinement details for studied complexes.

Crystal Structure	1	3	4	5
Chemical formula	C_14_H_8_N_2_O_2_ICl_3_Cu	C_14_H_8_N_2_O_2_I_3_ClCu	C_14_H_8_N_2_O_2_I_2_Cl_2_Cu ·0.5C_2_H_5_OH	C_14_H_8_N_2_O_2_IBr_2_ClCu
Formula weight	533.01	715.91	647.50	621.93
T/K	293(2)	293(2)	293(2)	293(2)
λ/Å	1.54184	1.54184	1.54184	0.71073
Crystal system	monoclinic	monoclinic	monoclinic	monoclinic
Space group	*Pc*	*P*2_1_/*c*	*I*2/*a*	*P*2_1_/*c*
*a*/Å	15.690(2)	10.1414(1)	12.2206(5)	9.913(1)
*b*/Å	4.1591(6)	14.4435(1)	15.5845(5)	14.177(1)
*c*/Å	12.466(1)	12.5537(2)	20.2814(7)	12.556(1)
*β*/°	92.26(1)	107.375(1)	97.883(3)	108.20(1)
*V*/Å^3^	812.9(2)	1754.93(4)	3826.1(2)	1676.4(3)
*Z*/*Z’*	2/1	4/1	8/1	4/1
*d*_calc/_g·cm^−3^	2.178	2.71	2.248	2.464
Θ range/°	5.64–68.53	4.57–68.03	4.40–68.11	2.60–25.03
*µ*/mm^−1^	21.433	44.681	29.651	8.079
Crystal size/mm^3^	0.18 × 0.07 × 0.02	0.32 × 0.14 × 0.03	0.36 × 0.17 × 0.08	0.35 × 0.14 × 0.02
Crystal color and form	green plate	green plate	green block	green plate
*R*_int_	0.092	0.088	0.033	0.084
Refl. coll./unique	8400/2596	19250/3172	12498/3461	10652/2948
Refl. with *I* > 2*σ*(*I*)	1767	3050	3045	1730
Param./restraints	208/2	212/0	235/0	208/0
*R*_1_; *wR*_2_ [*I* > 2*σ*(*I*)]	0.0678; 0.1671	0.0458; 0.1248	0.0396; 0.1132	0.0955; 0.2502
*R*_1_; *wR*_2_ [all data]	0.1027; 0.1964	0.0467; 0.1257	0.0442; 0.1164	0.1494; 0.2957
GooF on *F*^2^	1.066	1.059	1.04	1.039
ρ_min;_ ρ_max_/e·Å^−3^	−1.20/1.14	−1.60/1.53	−1.28/0.78	−2.12/1.00
CCDC No.	2446890	2446891	2446892	2446893

**Table 2:** The activity data of the tested complexes **1**–**5** expressed as MIC (MBC or MFC) [µg/mL] and {MBC/MIC or MFC/MIC} values against the reference strains of bacteria and fungi.

Species		MIC (MBC or MFC) [µg/mL] and {MBC/MIC or MFC/MIC} Values of the Studied Complexes and Positive Controls								
		1	2	3	4	5	CIP/VA * NY **	NIT	CFX	APC
**Gram-positive bacteria**	*Staphylococcus* *aureus* ATCC 25923	7.81 (7.81) {1}	15.62 (15.62) {1}	15.62 (31.25) {2}	3.91 (3.91) {1}	7.81 (15.62) {2}	0.48 (0.48) {1}	15.62 (15.62)	0.49	nd
	*Staphylococcus* *aureus* ATCC 29213	15.62 (15.62) {1}	15.62 (31.25) {2}	15.62 (31.25) {2}	7.81 (15.62) {2}	15.62 (15.62) {1}	0.48 (0.48) {1}	nd	nd	nd
	*Staphylococcus* *aureus* ATCC 6538	7.81 (7.81) {1}	15.62 (31.25) {2}	15.62 (31.25) {2}	15.62 (15.62) {1}	15.62 (31.25) {2}	0.24 (0.24) {1}	nd	nd	nd
	*Staphylococcus* *aureus* ATCC 43300	7.81 (15.62) {2}	15.62 (62.5) {4}	15.62 (62.5) {4}	7.81 (7.81) {1}	15.62 (15.62) {1}	0.24 (0.24) {1}	7.81	nd	nd
	*Staphylococcus* *epidermidis* ATCC 12228	1.95 (3.91) {2}	1.95 (3.91) {2}	3.91 (15.62) {4}	3.91 (7.81) {2}	7.81 (15.62) {2}	0.12 (0.12) {1}	3.91 (7.81)	0.24	nd
	*Enterococcus* *faecalis* ATCC 29212	3.91 (7.81) {2}	3.91 (15.62) {4}	15.62 (62.5) {4}	7.81 (15.62) {2}	15.62 (31.25) {2}	0.98 * (1.95) {2}	nd	nd	nd
	*Micrococcus* *luteus* ATCC 10240	0.98 (1.95) {2}	0.98 (1.95) {2}	3.91 (15.62) {4}	3.91 (3.91) {1}	7.81 (7.81) {1}	0.98 (1.95) {2}	62.5 (62.5)	0.98	nd
	*Bacillus* *subtilis* ATCC 6633	3.91 (3.91) {1}	7.81 (7.81) {1}	15.62 (15.62) {1}	15.62 (15.62) {1}	15.62 (15.62) {1}	0.03 (0.03) {1}	3.91 (3.91)	15.62	62.5
	*Bacillus* *cereus* ATCC 10876	3.91 (15.62) {4}	7.81 (31.25) {4}	15.62 (62.5) {4}	7.81 (31.25) {4}	7.81 (15.62) {2}	0.06 (0.12) {2}	7.81 (15.62)	31.25	nd
**Gram-negative bacteria**	*Klebsiella* *pneumoniae* ATCC 13883	1000 (>1000) {>1}	-	-	-	-	0.12 (0.24) {2}	15.62	nd	nd
	*Proteus* *mirabilis* ATCC 12453	1000 (>1000) {>1}	1000 (>1000) {>1}	-	-	-	0.03 (0.03) {1)	62.5	nd	nd
	*Escherichia* *coli* ATCC 25922	-	-	-	-	-	0.06 (0.06) {1}	7.81	nd	nd
	*Salmonella* *typhimurium* ATCC 14028	1000 (>1000) {>1}	1000 (>1000) {>1}	-	-	-	0.004 (0.008) {2}	31.25	nd	nd
	*Pseudomonas* *aeruginosa* ATCC 9027	-	-	-	-	-	0.48 (0.98) {2}	nd	nd	nd
**Fungi**	*Candida* *albicans* ATCC 10231	500 (500) {1}	500 (500) {1}	500 (500) {1}	250 (250) {1}	250 (500) {1}	0.48 * (0.48) {1}	na	na	na
	*Candida* *albicans* ATCC 2091	125 (500) {4}	250 (1000) {4}	500 (1000) {2}	500 (500) {1}	500 (500) {1}	0.24 * (0.24) {1}	na	na	na
	*Candida* *parapsilosis* ATCC 22019	250 (1000) {4}	500 (1000) {2}	500 (500) {1}	250 (250) {1}	500 (500) {1}	0.24 * (0.48) {2}	na	na	na
	*Candida* *glabrata* ATCC 90030	125 (250) {2}	250 (250) {1}	1000 (>1000) {>1}	125 (250) {2}	250 (500) {2}	0.24 * (0.48) {2}	na	na	na
	*Candida* *krusei* ATCC 14243	500 (>1000) {>2}	500 (>1000) {>2}	1000 (1000) {1}	250 (500) {2}	500 (1000) {2}	0.24 * (0.24) {1}	na	na	na
	*Candida* *auris* CDC 311903	500 (1000) {2}	500 (1000) {2}	1000 (1000) {1}	500 (500) {1}	500 (500) {1}	0.48 ** (0.48) {1}	na	na	na

The standard antimicrobial agents used as positive controls: ciprofloxacin (CIP), nitrofurantoin (NIT), cefuroxime (CFX) and ampicillin (APC) for bacteria (except enterococci), vancomycin (VA *) for enterococci and nystatin (NY **) for fungi; “-”, no activity; nd, not determined, na, not applicable.

## Data Availability

Data is contained within the article or Supplementary Material. CCDC Nos 2446890−2446893 contain the supplementary crystallographic data for this paper. The copies of these data can be obtained free of charge via http://www.ccdc.cam.ac.uk/data_request/cif (accessed on 20 July 2025) or on application to CCDC, 12 Union Road, Cambridge CB2 1EZ, UK; fax: (+44) 1223-336-033; or e-mail: deposit@ccdc.cam.ac.uk.

## Supplementary Materials

The following supporting information can be downloaded at: https://www.mdpi.com/article/10.3390/ijms262210980/s1.
