# Supplementary material for: Copper(II) Complexes of Selected Acylhydrazones as Potential Biological Agents
Source: Int J Mol Sci. 2025 Nov 13;26(22):10980. doi: 10.3390/ijms262210980 (PMC12652143; doi:10.3390/ijms262210980)
Supplement: Supplementary file 1 [file ijms-26-10980-s001.zip › ijms-3803064-supplementary.pdf]

International Journal of Molecular Sciences

MDPI

Article

Copper(II) complexes of selected acylhydrazones as potential biological agents

Izabela Czyżewska [1], Liliana Mazur [2], Robert Mroczka [3], Anna Biernasiuk [4], Anna Hordyjewska [5], Łukasz Popiołek [1]*

[1] Chair and Department of Organic Chemistry, Faculty of Pharmacy, Medical University of Lublin, 4A Chodźki Street, 20-093 Lublin, Poland;
[2] Institute of Chemical Sciences, Faculty of Chemistry, Maria Curie-Skłodowska University, Maria Curie-Skłodowska Sq. 2, 20-031 Lublin, Poland;
[3] Laboratory of X-ray Optics, Department of Biomedical and Analytical Chemistry, Faculty of Medicine, The John Paul II Catholic University of Lublin, 1J Konstantynów Street, 20-708 Lublin, Poland;
[4] Chair and Department of Pharmaceutical Microbiology, Faculty of Pharmacy, Medical University of Lublin, 1 Chodźki Street, 20-093 Lublin, Poland;
[5] Department of Medicinal Chemistry, Faculty of Medical Sciences, Medical University of Lublin, 4A Chodźki Street, 20-093 Lublin, Poland;
* corresponding author e-mail: lukasz.popiolek@umlub.pl

Supplementary Materials

1. X-ray crystallography

Crystal data for complex **2**: $C_{14}H_8N_2O_2IBr_2ClCu$, M = 621.93 g·mol^{-1}, monoclinic, space group *Pc*, a = 15.7584(9) Å, b = 4.2321(3) Å, c = 12.7133(7) Å, β = 92.784(5)°, V = 846.9(1) Å^3, Z = 2.

Due to very small size of crystal **2** used for X-ray diffraction studies and, as a result, low data-to-parameter ratio the crystal structure refinement was unstable, which made it impossible to fully refine the model and to obtaining sufficient quality data for this structure.

A. Molecular structure

Table S1. Selected bond distances (Å) in complexes

Bond	1	3	4	5
**Cu–O1**	1.99(2)	1.986(5)	1.977(4)	1.990(11)
**Cu–N2**	1.94(2)	1.939(5)	1.929(4)	1.939(14)
**Cu–O2**	1.85(2)	1.904(4)	1.890(4)	1.878(11)
**Cu–Cl1**	2.248(7)	2.215(2)	2.229(2)	2.210(5)
**C1–O1**	1.26(3)	1.237(8)	1.270(7)	1.238(19)
**C1–N1**	1.32(3)	1.332(9)	1.322(7)	1.30(2)
**N1–N2**	1.39(2)	1.382(7)	1.390(6)	1.380(18)
**N2–C2**	1.30(3)	1.289(8)	1.289(7)	1.30(2)
**C2–C3**	1.40(3)	1.419(9)	1.429(7)	1.44(2)
**C4–O2**	1.31(3)	1.307(7)	1.307(7)	1.298(18)
**C5–I1**	-	2.099(6)	2.091(5)	-
**C5–Cl2**	1.73(2)	-	-	-
**C5–Br1**	-	-	-	1.916(17)
**C7–Cl2/Cl3**	1.70(2)	-	1.735(6)	-
**C7–Br**	-	-	-	1.974(16)
**C7–I2**	-	2.105(6)	-	-
**C10–I1/I2/I3**	-	2.085(8)	2.080(6)	2.11(2)
**C11–I1**	2.12(2)	-	-	-

Table S2. Selected bond angles (°) in crystals of studied complexes

Angle	1	3	4	5
**O2–Cu–O1**	171.6(7)	172.01(19)	172.65(16)	170.9(5)
**O2–Cu–N2**	92.2(8)	91.56(19)	91.89(17)	91.2(5)
**N2–Cu–O1**	80.8(7)	80.8(2)	80.90(16)	80.2(5)
**O2–Cu–Cl1**	91.5(5)	95.51(14)	95.10(12)	95.3(3)
**N2–Cu–Cl1**	171.4(6)	171.55(16)	171.51(15)	172.2(4)
**O1–Cu–Cl1**	94.9(5)	92.3(15)	92.2(12)	93.5(3)
**O1–C1–N1**	119.0(2)	121.1(6)	119.7(5)	118.5(15)
**O1–C1–C9**	121.0(2)	121.1(6)	120.5(5)	119.6(15)
**N1–C1–C9**	120.0(2)	117.4(6)	119.8(5)	121.7(15)
**C1–N1–N2**	116.0(2)	114.1(5)	114.6(4)	117.4(14)
**C2–N2–N1**	121.2(18)	118.8(5)	118.0(4)	120.6(15)
**N2–C2–C3**	124(2)	123.3(6)	122.5(5)	123.0(17)
**C2–C3–C4**	123.0(2)	122.6(6)	122.8(5)	122.3(15)
**C1–C9–C10**	117.0(2)	126.4(6)	125.4(5)	125.6(16)

Table S3. Selected torsion angles (°) in studied crystals.

Torsion angle	1	3	4	5
**C9–C1–N1–N2**	179.0(18)	169.5(5)	175.3(5)	-170.2(15)
**O1–C1–N1–N2**	-2(3)	-3.9(9)	-2.5(8)	-4(2)
**C1–N1–N2–C2**	-174(2)	-170.9(6)	-175.6(5)	-171.7(16)
**N1–N2–C2–C3**	-178(2)	177.3 (6)	178.8(5)	-178.6(15)
**N2–C2–C3–C4**	-4(4)	3.0(10)	-0.4(9)	2(3)
**C2–C3–C4–O2**	6(4)	-5.7(10)	-1.7(9)	-3(3)
**N1–C1–C9–C10**	158(2)	55.1(9)	47.0(8)	55(2)
**O1–C1–C9–C10**	-21(3)	-131.6(7)	-135.2(6)	-130.1(18)

B. Crystal structure and intermolecular interactions

In isostructural crystals **3** and **5** the hydrazone N1 atom serves as a hydrogen bond donor to the chloride Cl1 anion of the adjacent *c*-glide plane related monomer, forming a molecular chain propagated along the *c* axis (Fig. S1-a). The inversion related chains are linked by combination of weak hydrogen bonds C8–H8···I3$^{(vi)}$, C11–H11···I2$^{(vi)}$, C12–H12···I3$^{(viii)}$, C2–H2···Cl1$^{(v)}$ (symmetry codes as in Table S4). These interactions extend the alternately arranged zig-zag chains into (100) supramolecular layers (Fig. S1-b,c). The forces responsible for stabilization of folded layers into 3D architecture (Fig. S1-d) are I2···I1 [$d_{I\cdots I}$ = 3.987 Å; <C–I···I = 147°] halogen bonds, supported by Hal…π interactions, involving C10–I3 (**3**) or C10–I1 (**5**) as a donor and the aromatic C3<<C8 moiety as an acceptor.

Similarly, in complex **1** the main 1D motifs are supramolecular chains composed of *c*-glide plane related molecules, combined by strong N1–H1n···Cl1$^{(i)}$ hydrogen bonds, supported by weak C8–H8···Cl2$^{(i)}$, C14–H14···Cl1$^{(i)}$ interactions (Fig. S2-a). The chains constitute building blocks of folded (100) layers (Fig. S2-b,c) with the Hal···π interactions and π-stacking contacts between overlapping aromatic rings from the adjacent chains (Fig. S2-b). Among the interactions responsible for linking the double layers into the stable 3D net the I1···Cl3 [$d_{I\cdots Cl}$ = 3.505 Å; <C–I···Cl = 159°] (Fig. S2-c) halogen bonds seem to be noticeable.

In spite of inclusion of ethanol molecules into crystal **4**, the main supramolecular motifs observed in unsolvated crystals, *i.e.* the supramolecular zig-zag chains, generated by strong N1–H1n···Cl1$^{(vii)}$, C2–H2···Cl1$^{(vii)}$ hydrogen bonds are preserved (Fig. S3-a). However, in this case the chains propagate along the crystallographic *a* axis. The neighboring chains, related to one another by inversion and hence running antiparallel, are connected by a combination of weak C8–H8···I1$^{(viii)}$, C11–H11···Cl2$^{(viii)}$, C13–H13···Cl1$^{(viii)}$ hydrogen bonds into folded layers, parallel to the (001) plane (Fig. S3-b,c). The solvent molecules, which are disordered over the 2-fold axis, fill in the gaps between molecules from

adjacent layers. The interactions between intercalated ethanol molecules and the 'host' moieties are realized by O3–H3···Cl1, C16–H162···I2$^{(ix)}$, C16–H161···Cl2$^{(x)}$ hydrogen-bond type contacts.

Table S4. Hydrogen bonds lengths (Å) and angles (°) in studied crystals.

D–H···A	d(D–H) / Å	d(H···A) / Å	d(D···A) / Å	<DHA / °
**1**				
N1–H1n···Cl1$^{(i)}$	0.98	2.31	3.28(3)	174
C2–H2···Cl1$^{(i)}$	0.93	3.06	3.83(3)	142
C8–H8···Cl2$^{(i)}$	0.93	2.73	3.59(3)	155
C14–H14···Cl1$^{(i)}$	0.93	2.85	3.66(4)	149
C12–H12···Cl3$^{(ii)}$	0.93	3.08	3.74(4)	130
C13–H13···I1$^{(iii)}$	0.93	3.34	4.11(4)	141
**3**				
N1–H1n···Cl1$^{(iv)}$	0.94(8)	2.23(8)	3.162(6)	171(6)
C2–H2···Cl1$^{(v)}$	0.93	2.96	3.590(6)	126
C8–H8···I3$^{(vi)}$	0.93	3.18	4.108(7)	173
C11–H11···I2$^{(vi)}$	0.93	3.26	4.181(8)	170
C12–H12···I3$^{(iv)}$	0.93	3.25	4.042(8)	145
**4**				
N1–H1n···Cl1$^{(vii)}$	0.86	2.40	3.154(6)	146
O3–H3···Cl1	0.82	2.81	3.241(5)	115
C2–H2···Cl1$^{(vii)}$	0.93	2.99	3.709(7)	135
C8–H8···I1$^{(viii)}$	0.93	3.26	4.099(8)	151
C11–H11···Cl2$^{(viii)}$	0.93	3.04	3.750(9)	134
C13–H13···Cl1$^{(viii)}$	0.93	2.94	3.726(9)	143
C16–H162···I2$^{(ix)}$	0.96	2.30	3.113(5)	142
C16–H161···Cl2$^{(x)}$	0.96	2.78	3.401(5)	123
**5**				
N1–H1n···Cl1$^{(iv)}$	0.86	2.41	3.21(1)	155
C2–H2···Cl1$^{(v)}$	0.93	2.98	3.53(2)	119
C8–H8···I1$^{(vi)}$	0.93	3.15	4.07(2)	172
C11–H11···Br2$^{(vi)}$	0.93	3.17	4.09(3)	172
C12–H12···I1$^{(iv)}$	0.93	3.18	3.96(3)	145
**Symmetry codes:** (i) *x*, -*y*+1, *z*-1/2; (ii) *x*+1, *y*, *z*; (iii) *x*, -*y*+2, *z*-1/2; (iv) *x*, -*y*+1/2, *z*-1/2; (v) -*x*+1, *y*+1/2, -*z*+3/2; (vi) -*x*+1,-*y*+1, -*z*+1; (vii) *x*-1/2, -*y*+1, *z*; (viii) -*x*+1/2, -*y*+3/2, -*z*+1/2; (ix) *x*+1/2, *y*-1/2, *z*+1/2; (x) -*x*+3/2, *y*, -*z*+1				

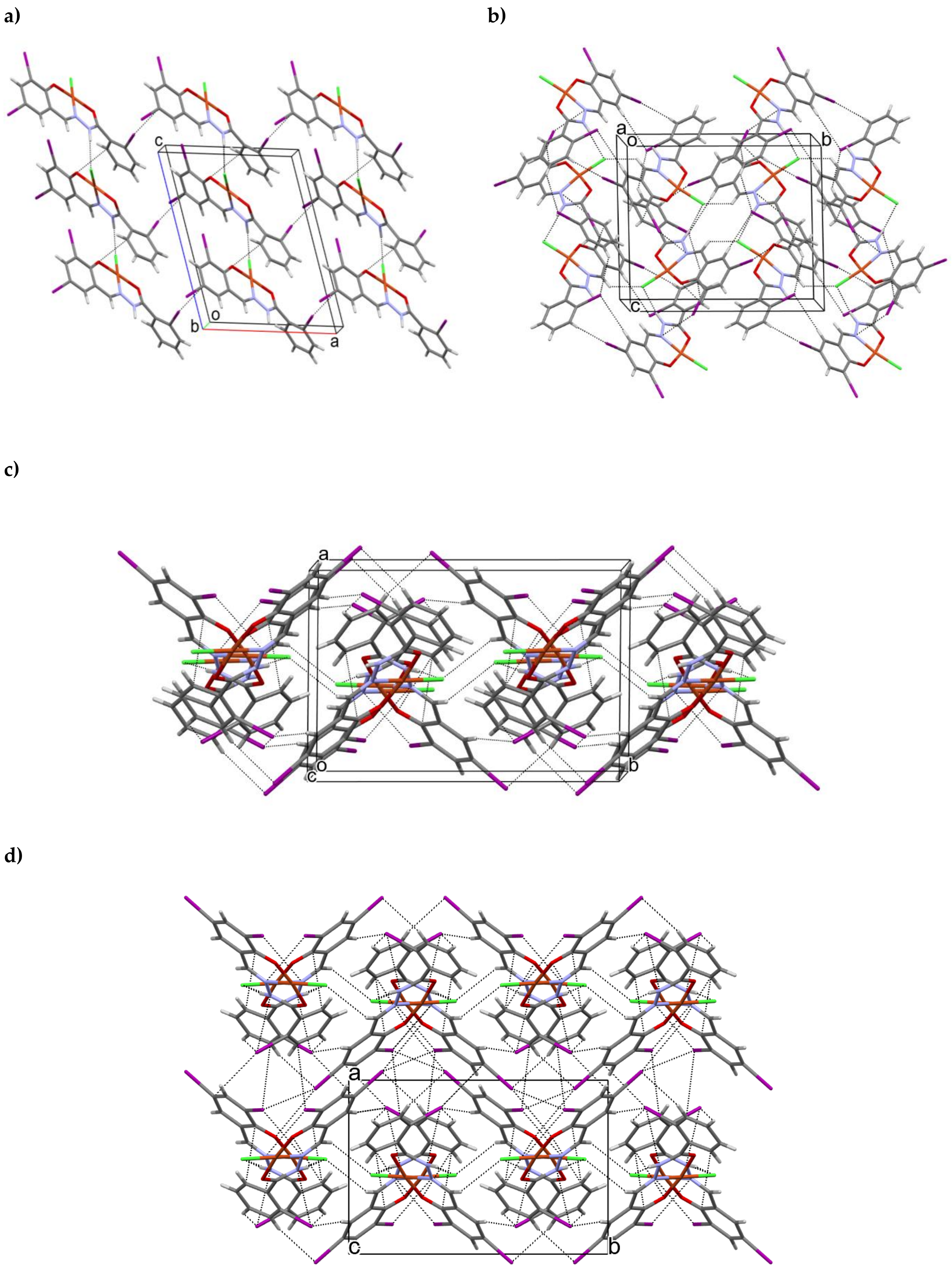

Figure S1. Part of crystal structure **3** visualizing: a) [001] supramolecular chains stabilized by strong N1–H1n···Cl1 hydrogen bonds; b) (100) supramolecular layer stabilized by weak C–H···Hal hydrogen bonds; c) (100) folded layer in view along the *c* axis; d) crystal packing in view along the *c* axis, with interlayer halogen bonds. Dashed lines indicate hydrogen or halogen bonds.

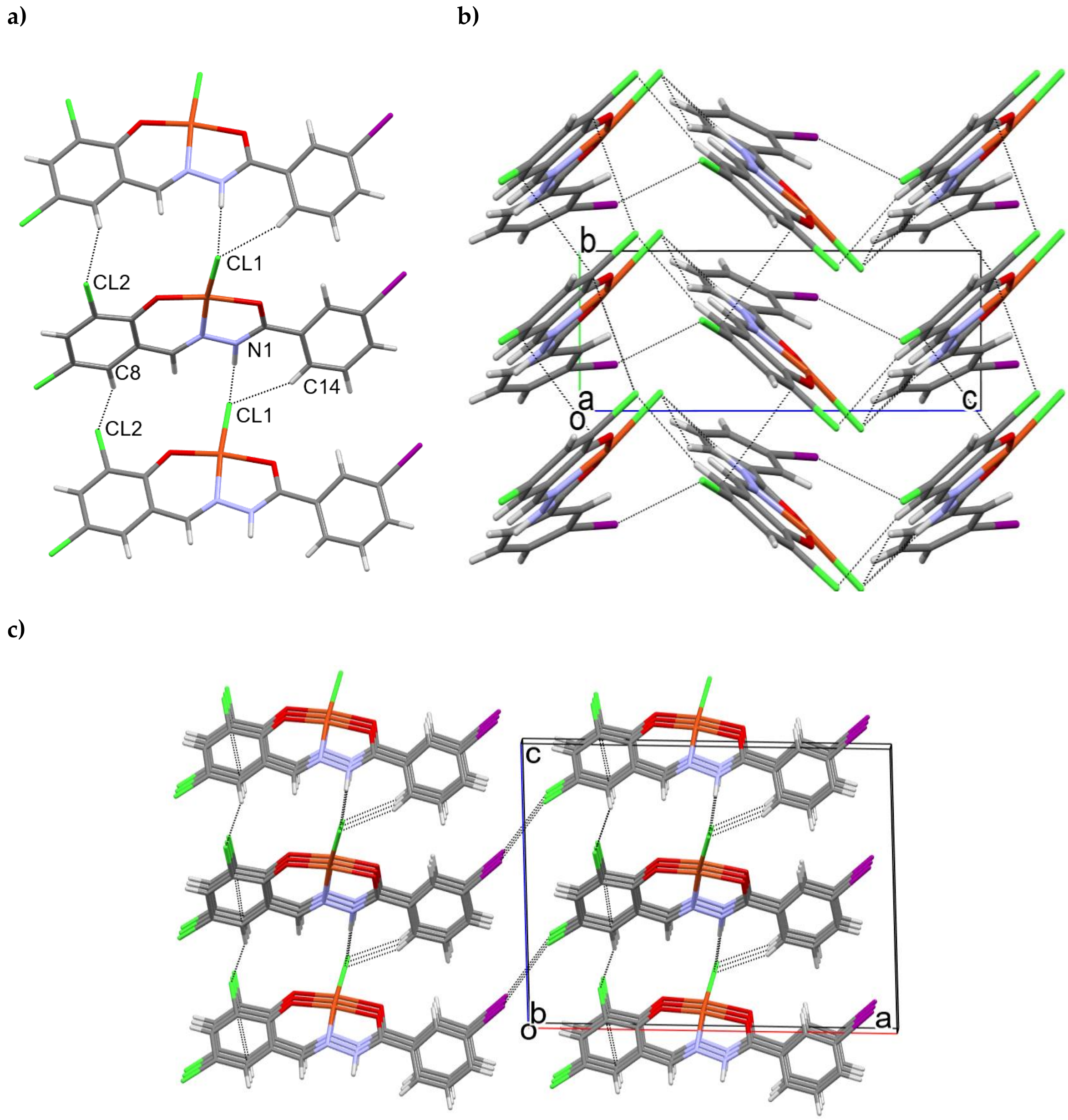

Figure S2. Crystal structure **1**: a) molecular chains *via* strong N1–H1n···Cl1 hydrogen bonds; b) part of (100) folded layer composed of stacking chains; c) packing of molecules in view along the *b* axis, with Cl3···I1 halogen bonds between (100) layers.

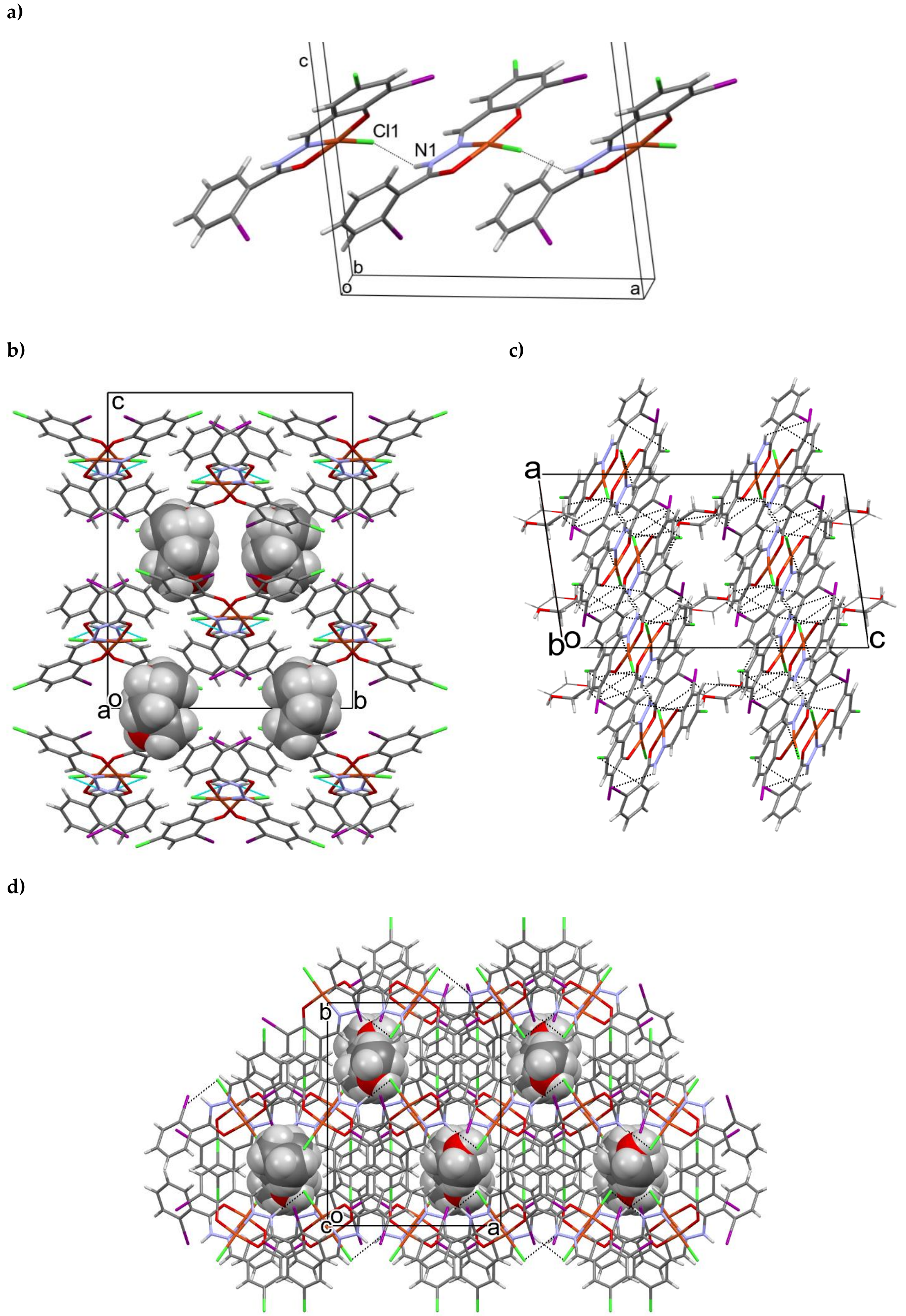

Figure S3. Crystal structure **4**: a) molecular chain sustained by N1–H1n···Cl1 hydrogen bonds; b) crystal packing in view along the *a* axis, with space fill representation of the 'guest' molecules; c) (001) molecular layers intercalated by ethanol molecules, in view along the *b* axis; d) crystal packing in view along the *c* axis. Dashed lines indicate hydrogen and halogen bonds.

2. UV-Vis spectroscopy

a)

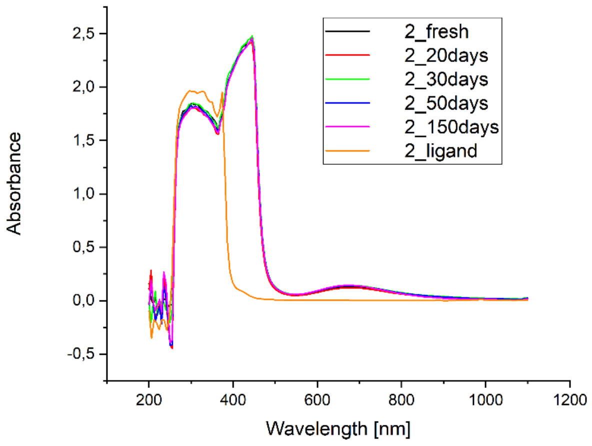

b)

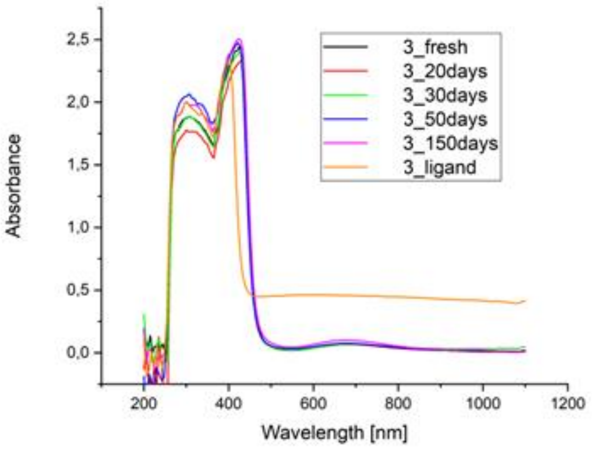

c)

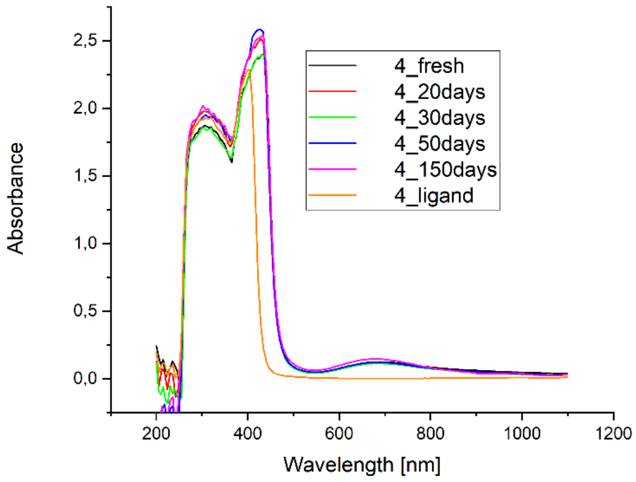

Figure S4. UV-Vis spectra of copper(II) complexes **2 - 4** and the respective ligands in DMSO, recorded for the fresh solutions as well as after 20, 30, 50 and 150 days.

Table S5. Selected UV-VIS bands of copper(II) complexes **1 - 5** and the respective ligands in DMSO

No of Cu(II) complex	Band 1, wavelength [nm]	Band 2, wavelength [nm]	Band 3, wavelength [nm]
**1**	310	440	670
**2**	310	440	675
**3**	310	425	680
**4**	310	425	685
**5**	310	425	685

3. IR spectroscopy

Table S6. Absorption ranges of chemical groups of complexes

**IR** (cm^{-1})	**1**	**2**	**3**	**4**	**5**
OH	-	-	-	3364	-
NH	3162	3166	3151	3166	3153
CH, arom.	3046	3058, 3013	3046, 3022	3044	3058, 3026
C=N	1611	1608	1612	1607	1614
C_{ar}=C_{ar}	1592, 1582, 1542, 1469	1589, 1566, 1540, 1469	1574, 1558, 1545, 1486	1584, 1570, 1521, 1489	1586, 1570, 1560, 1545, 1468
C–N, amide	1515	1502	1517	1521	1499
N–N	1064	1063	1046	1055	1047

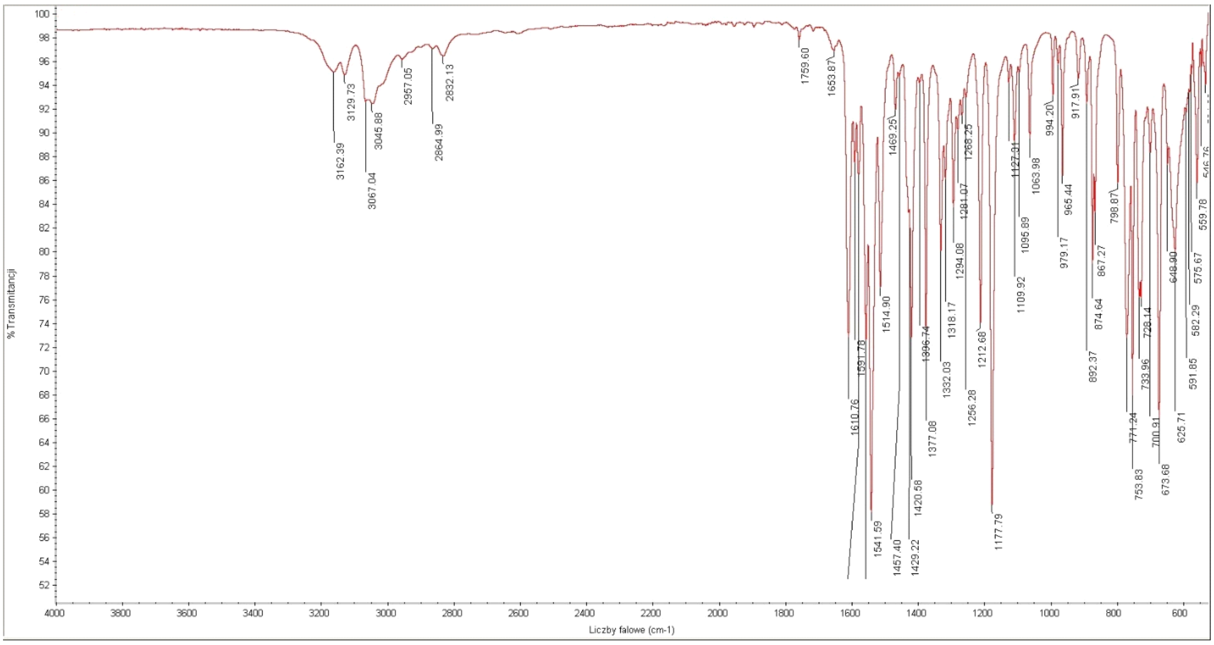

Figure S5. FT-IR spectrum of complex **1**

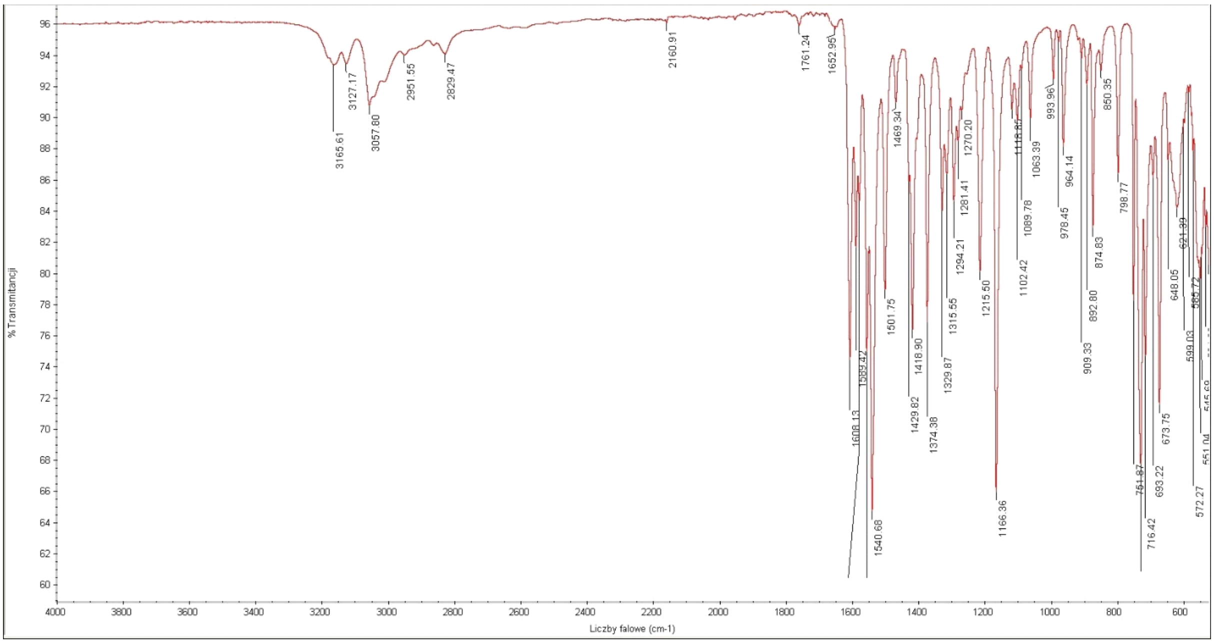

Figure S6. FT-IR spectrum of complex **2**

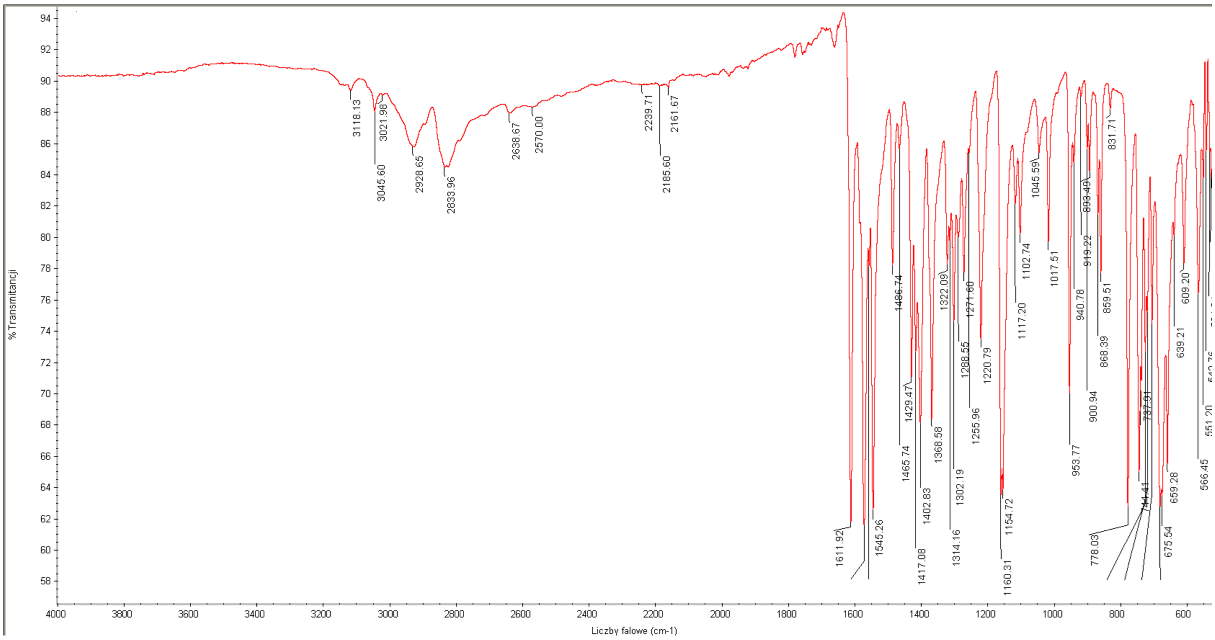

Figure S7. FT-IR spectrum of complex **3**

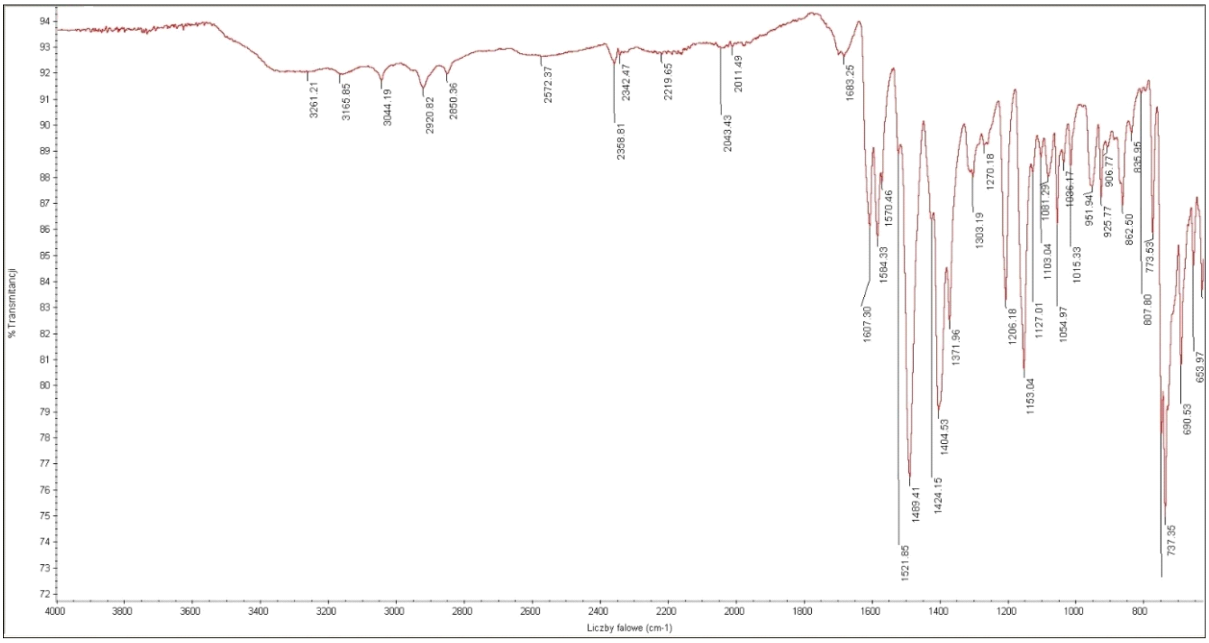

Figure S8. FT-IR spectrum of complex **4**

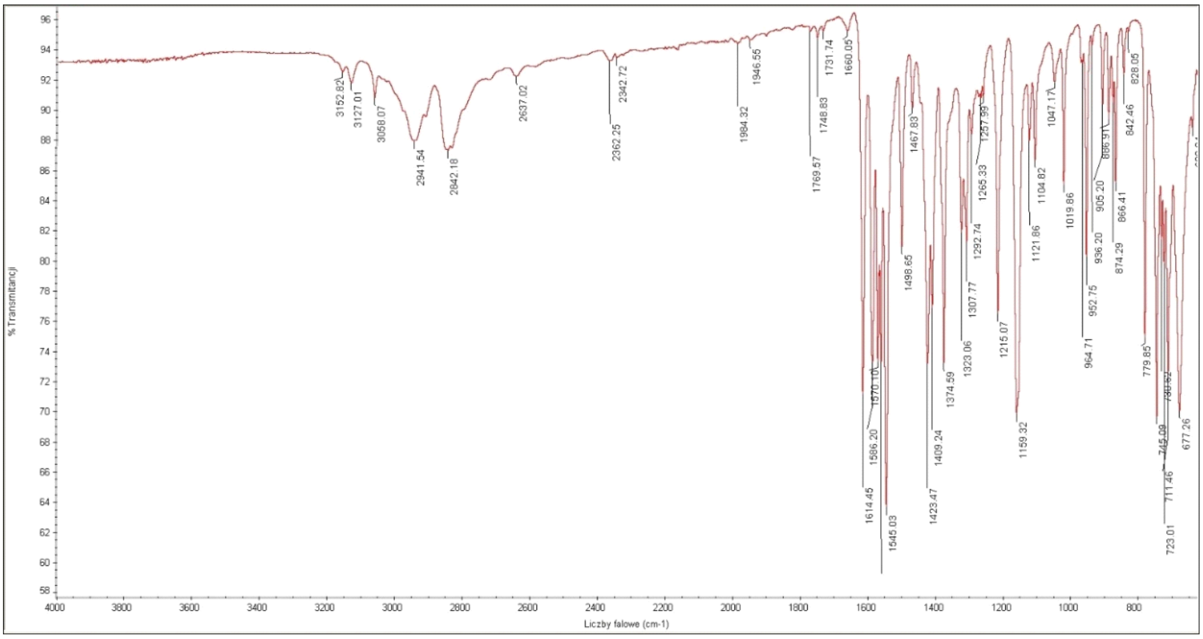

Figure S9. FT-IR spectrum of complex **5**

4. Microbiology

In vitro antimicrobial assay procedures

The examined complexes **1 – 5** were screened *in vitro* for antibacterial and antifungal activities using the broth microdilution method according to European Committee on Antimicrobial Susceptibility Testing (EUCAST) [1] and

Clinical and Laboratory Standards Institute guidelines [2] against a panel of reference strains of microorganisms, including Gram-positive bacteria (*Staphylococcus aureus* ATCC 43300 (*Methicillin Resistant S. aureus* – MRSA), *Staphylococcus aureus* ATCC 29213 (*Methicillin Susceptible S. aureus* – MSSA), *Staphylococcus aureus* ATCC 25923 (*Methicillin Susceptible S. aureus* – MSSA), *Staphylococcus aureus* ATCC 6538 (*Methicillin Susceptible S. aureus* – MSSA), *Staphylococcus epidermidis* ATCC 12228, *Enterococcus faecalis* ATCC 29212, *Micrococcus luteus* ATCC 10240, *Bacillus subtilis* ATCC 6633 and *Bacillus cereus* ATCC 10876), Gram-negative bacteria (*Escherichia coli* ATCC 25922, *Klebsiella pneumoniae* ATCC 13883, *Proteus mirabilis* ATCC 12453, *Salmonella typhimurium* ATCC 14028 and *Pseudomonas aeruginosa* ATCC 9027) and fungi belonging to yeasts (*Candida albicans* ATCC 10231, *Candida albicans* ATCC 2091, *Candida parapsilosis* ATCC 22019, *Candida glabrata* ATCC 90030, *Candida krusei* ATCC 14243 and *Candida auris* CDC 311903). The microorganisms came from American Type Culture Collection (ATCC) or Centers for Disease Control (CDC), routinely used for the evaluation of antimicrobials. All the used microbial cultures were first subcultured on nutrient agar or Sabouraud agar at 35°C for 18-24 h or 30°C for 24-48 h for bacteria and fungi, respectively.

The surface of Mueller-Hinton agar (for bacteria) and RPMI 1640 with MOPS (for fungi) were inoculated with the suspensions of bacterial or fungal species. Microbial suspensions were prepared in sterile saline with an optical density of McFarland standard scale 0.5.

Samples containing examined complexes **1 – 5** were first dissolved in sterile water at a concentration of 20 mg/mL. The tested substances were then diluted 20-fold in an appropriate liquid medium and used for further testing. Subsequently MIC (Minimal Inhibitory Concentration) of the compounds was examined by the microdilution broth method, using their two-fold dilutions in Mueller-Hinton broth (for bacteria) and RPMI 1640 broth with MOPS (for fungi) prepared in 96-well polystyrene plates. Final concentrations of the compounds ranged from 1000 to 0.488 μg/mL. Microbial suspensions were prepared in 0.85% NaCl with an optical density of 0.5 McFarland standard, which corresponds to 1.5 x 10^8 CFU/mL (Colony Forming Units/ml) – bacterial colony forming units/mL or 5 x 10^6 CFU/mL – fungal colony forming units/m, respectively. Next each bacterial or fungal suspension was added per each well containing broth and various concentrations of the examined compounds. After incubation, the MIC was assessed spectrophotometrically as the lowest concentration of the samples showing complete bacterial or fungal growth inhibition. Appropriate growth and sterile controls were carried out. The media with and without tested substances or sterile water were also used as controls. The inhibition of microbial growth was judged by comparison with a control culture prepared without any sample tested. Ciprofloxacin, vancomycin, nitrofurantoin, cefuroxime, ampicillin or nystatin (Sigma-Aldrich Chemicals, St. Louis, MO, USA) were used as a reference antibacterial or antifungal compounds, respectively.

The MBC (Minimal Bactericidal Concentration) or MFC (Minimal Fungicidal Concentration) are defined as the lowest concentration of the compounds that is required to kill a particular bacterial or fungal species. MBC or MFC was determined by removing the culture using for MIC determinations from each well and spotting onto appropriate agar medium. The plates were incubated under appropriate conditions for bacteria and fungi. The lowest complexes concentrations with no visible growth observed were assessed as a bactericidal or fungicidal concentration. All the experiments were repeated three times and representative data is presented [3, 4].

In this study, no bioactivity was defined as a MIC > 1000 μg/mL, mild bioactivity as a MIC in the range 501 – 1000 μg/mL, moderate bioactivity with MIC from 126 to 500 μg/mL, good bioactivity as a MIC in the range 26 – 125 μg/mL, strong bioactivity with MIC between 10 and 25 μg/mL and very strong bioactivity as a MIC < 10 μg/mL [5]. The MBC/MIC or MFC/MIC ratios were calculated to determine bactericidal/fungicidal (MBC/MIC ≤ 4, MFC/MIC ≤ 4) or bacteriostatic/fungistatic (MBC/MIC > 4, MFC/MIC > 4) effect of the tested compounds.

Results

The obtained results were also summarized in the form of a heatmap presented below (Table S7).

Table S7. The MIC heatmap of the antimicrobial spectrum of the tested complexes 1 – 5. (In the matrix, the MIC values (μg/mL) are color-coded, and the color key is shown on the right in this figure)

Species		MIC (μg/mL) values of the studied complexes				
		**1**	**2**	**3**	**4**	**5**
	Staphylococcus aureus ATCC 25923	7.81	15.62	15.62	3.91	7.81
**Gram-positive bacteria**	*Staphylococcus aureus* ATCC 29213	15.62	15.62	15.62	7.81	15.62
	Staphylococcus aureus ATCC 6538	7.81	15.62	15.62	15.62	15.62
	Staphylococcus aureus ATCC 43300	7.81	15.62	15.62	7.81	15.62
	Staphylococcus epidermidis ATCC 12228	1.95	1.95	3.91	3.91	7.81
	Enterococcus faecalis ATCC 29212	3.91	3.91	15.62	7.81	15.62
	Micrococcus luteus ATCC 10240	0.98	0.98	3.91	3.91	7.81
	Bacillus subtilis ATCC 6633	3.91	7.81	15.62	15.62	15.62
	Bacillus cereus ATCC 10876	3.91	7.81	15.62	7.81	7.81
**Gram-negative bacteria**	*Klebsiella pneumoniae* ATCC 13883	1000	>1000	>1000	>1000	>1000
	Proteus mirabilis ATCC 12453	1000	1000	>1000	>1000	>1000
	Escherichia coli ATCC 25922	>1000	>1000	>1000	>1000	>1000
	Salmonella typhimurium ATCC 14028	1000	1000	>1000	>1000	>1000
	Pseudomonas aeruginosa ATCC 9027	>1000	>1000	>1000	>1000	>1000
**Fungi**	*Candida albicans* ATCC 10231	500	500	500	250	250
	Candida albicans ATCC 2091	125	250	500	500	500

	MIC (μg/mL) values	
	>1000	no bioactivity
	1000	mild bioactivity
	500	moderate activity
	250	
	125	good bioactivity
	62.5	
	31.25	
	15.62	strong bioactivity
	7.81	very strong bioactivity
	3.91	
	1.95	
	0.98	

	Candida parapsilosis ATCC 22019	250	500	500	250	500
	Candida glabrata ATCC 90030	125	250	1000	125	250
	Candida krusei ATCC 14243	500	500	1000	250	500
	Candida auris CDC 311903	500	500	1000	500	500

International Journal of *Molecular Sciences*

MDPI

Table S8. The activity data of the tested complexes and positive controls expressed as MIC [µg/mL] of triplicate *in vitro* screening with mean and standard deviation (+/- SD) against the reference strains of bacteria and fungi

Species		MIC [µg/mL] values of the studied complexes and positive controls of triplicate *in vitro* screening with mean and standard deviation (+/- SD)								
		**1**	**2**	**3**	**4**	**5**	**CIP/VA*<br>NY****	**NIT**	**CFX**	**APC**
**Gram-positive bacteria**	*Staphylococcus aureus* ATCC 25923	7.81<br>7.81<br>15.62<br>**10.41**<br>**(+/- 4.51)**	15.62<br>15.62<br>15.62<br>**15.62**<br>**(+/- 0)**	15.62<br>31.25<br>15.62<br>**20.83**<br>**(+/- 9.02)**	3.91<br>7.81<br>1.95<br>**4.56**<br>**(+/- 2.98)**	7.81<br>3.91<br>7.81<br>**6.51**<br>**(+/- 2.25)**	0.48<br>0.24<br>0.98<br>**0.57**<br>**(+/- 0.38)**	15.62	0.49	nd
	Staphylococcus aureus ATCC 29213	15.62<br>15.62<br>7.81<br>**13.02**<br>**(+/- 4.51)**	15.62<br>31.25<br>7.81<br>**18.23**<br>**(+/- 11.94)**	15.62<br>15.62<br>31.25<br>**20.83**<br>**(+/- 9.02)**	7.81<br>7.81<br>15.62<br>**10.41**<br>**(+/- 4.51)**	15.62<br>15.62<br>7.81<br>**13.02**<br>**(+/- 4.51)**	0.48<br>0.24<br>0.98<br>**0.57**<br>**(+/- 0.38)**	nd	nd	nd
	Staphylococcus aureus ATCC 6538	7.81<br>7.81<br>15.62<br>**10.41**<br>**(+/- 4.51)**	15.62<br>15.62<br>7.81<br>**13,02**<br>**(+/- 4.51)**	15.62<br>31.25<br>15.62<br>**20.83**<br>**(+/- 9.02)**	15.62<br>15.62<br>7.81<br>**13.02**<br>**(+/- 4.51)**	15.62<br>31.25<br>15.62<br>**20.83**<br>**(+/- 9.02)**	0.24<br>0.24<br>0.48<br>**0.32**<br>**(+/- 0.14)**	nd	nd	nd
	Staphylococcus aureus ATCC 43300	7.81<br>15.62<br>7.81<br>**15.62**	15.62<br>15.62<br>7.81<br>**13.02**	15.62<br>7.81<br>15.62<br>**13.02**	7.81<br>7.81<br>15.62<br>**10.41**	15.62<br>7.81<br>31.25<br>**18.23**	0.24<br>0.24<br>0.12<br>**0.20**	7.81	nd	nd

		**(+/- 13.53)**	**(+/- 4.51)**	**(+/- 4.51)**	**(+/- 4.51)**	**(+/- 11.94)**	**(+/- 0.07)**			
	Staphylococcus epidermidis ATCC 12228	1.95<br>3.91<br>1.95<br>**2.60**<br>**(+/- 1.13)**	1.95<br>1.95<br>0.98<br>**1.63**<br>**(+/- 0.56)**	3.91<br>3.91<br>1.95<br>**3.26**<br>**(+/- 1.13)**	3.91<br>1.95<br>7.81<br>**4.56**<br>**(+/- 2.98)**	7.81<br>15.62<br>7.81<br>**10.41**<br>**(+/- 4.51)**	0.12<br>0.12<br>0.24<br>**0.16**<br>**(+/- 0.07)**	3.91	0.24	nd
	Enterococcus faecalis ATCC 29212	3.91<br>3.91<br>1.95<br>**3.26**<br>**(+/- 1.13)**	3.91<br>7.81<br>1.95<br>**4.56**<br>**(+/- 2.98)**	15.62<br>15.62<br>31.25<br>**20.83**<br>**(+/- 9.02)**	7.81<br>7.81<br>7.81<br>**7.81**<br>**(+/- 0)**	15.62<br>15.62<br>31.25<br>**20.83**<br>**(+/- 9.02)**	0.98*<br>1.95*<br>0.98*<br>**1.30***<br>**(+/- 0.56)**	nd	nd	nd
	Micrococcus luteus ATCC 10240	0.98<br>1.95<br>0.98<br>**1.30**<br>**(+/- 0.56)**	0.98<br>0.98<br>0.98<br>**0.98**<br>**(+/- 0)**	3.91<br>7.81<br>3.91<br>**5.21**<br>**(+/- 2.25)**	3.91<br>3.91<br>1.95<br>**3.26**<br>**(+/- 1.13)**	7.81<br>15.62<br>3.91<br>**9.11**<br>**(+/- 5.96)**	0.98<br>0.98<br>1.95<br>**1.30**<br>**(+/- 0.56)**	62.5	0.98	nd
	Bacillus subtilis ATCC 6633	3.91<br>3.91<br>7.81<br>**5.21**<br>**(+/- 2.25)**	7.81<br>3.91<br>3.91<br>**5.21**<br>**(+/- 2.25)**	15.62<br>15.62<br>15.62<br>**15.62**<br>**(+/- 0)**	15.62<br>15.62<br>7.81<br>**13.02**<br>**(+/- 4.51)**	15.62<br>7.81<br>31.25<br>**18.23**<br>**(+/- 11.94)**	0.03<br>0.06<br>0.03<br>**0.04**<br>**(+/- 0.02)**	3.91	15.62	62.5
	Bacillus cereus ATCC 10876	3.91<br>3.91<br>7.81<br>**5.21**<br>**(+/- 2.25)**	7.81<br>15.62<br>3.91<br>**9.11**<br>**(+/- 5.96)**	15.62<br>15.62<br>15.62<br>**15.62**<br>**(+/- 0)**	7.81<br>15.62<br>7.81<br>**10.41**<br>**(+/- 4.51)**	7.81<br>7.81<br>15.62<br>**10.41**<br>**(+/- 4.51)**	0.06<br>0.03<br>0.12<br>**0.07**<br>**(+/- 0.05)**	7.81	31.25	nd
**Gra**	*Klebsiella pneumoniae*	1000<br>1000	-	-	-	-	0.12<br>0.24	15.62	nd	nd

	ATCC 13883	1000<br>**1000**<br>**(+/- 0)**					0.12<br>**0.16**<br>**(+/- 0.07)**			
	Proteus mirabilis ATCC 12453	1000<br>1000<br>500<br>**833.33**<br>**(+/- 288.68)**	1000<br>1000<br>500<br>**833.33**<br>**(+/- 288.68)**	-	-	-	0.03<br>0.03<br>0.06<br>**0.04**<br>**(+/- 0.02)**	62.5	nd	nd
	Escherichia coli ATCC 25922	-	-	-	-	-	0.06<br>0.03<br>0.06<br>**0.05**<br>**(+/- 0.02)**	7.81	nd	nd
	Salmonella typhimurium ATCC 14028	1000<br>1000<br>1000<br>**1000**<br>**(+/- 0)**	1000<br>1000<br>500<br>**833.33**<br>**(+/- 288.68)**	-	-	-	0.004<br>0.004<br>0.008<br>**0.005**<br>**(+/- 0.002)**	31.25	nd	nd
	Pseudomonas aeruginosa ATCC 9027	-	-	-	-	-	0.48<br>0.12<br>0.98<br>**0.53**<br>**(+/- 0.43)**	nd	nd	nd
**Fungi**	*Candida albicans* ATCC 10231	500<br>1000<br>500<br>**666.67**<br>**(+/- 288.68)**	500<br>500<br>500<br>**500**<br>**(+/- 0)**	500<br>500<br>1000<br>**666.67**<br>**(+/- 288.68)**	250<br>250<br>500<br>**333.33**<br>**(+/- 144.34)**	250<br>125<br>250<br>**208.33**<br>**(+/- 72.17)**	0.48**<br>0.24**<br>0.48**<br>**0.40****<br>**(+/- 0.14)**	na	na	na

	Candida albicans ATCC 2091	125<br>125<br>250<br>**166.67**<br>**(+/- 72.17)**	250<br>250<br>125<br>**208.33**<br>**(+/- 72.17)**	500<br>500<br>250<br>**416.67**<br>**(+/- 144.34)**	500<br>500<br>500<br>**500**<br>**(+/- 0)**	500<br>500<br>250<br>**416.67**<br>**(+/- 144.34)**	0.24**<br>0.48**<br>0.24**<br>**0.32****<br>**(+/- 0.14)**	na	na	na
	Candida parapsilosis ATCC 22019	250<br>250<br>500<br>**333.33**<br>**(+/- 144.34)**	500<br>1000<br>500<br>**666.67**<br>**(+/- 288.68)**	500<br>500<br>500<br>**500**<br>**(+/- 0)**	250<br>250<br>500<br>**333.33**<br>**(+/- 144.34)**	500<br>250<br>500<br>**416.67**<br>**(+/- 144.34)**	0.24**<br>0.12**<br>0.24**<br>**0.20****<br>**(+/- 0.07)**	na	na	na
	Candida glabrata ATCC 90030	125<br>125<br>250<br>**166.67**<br>**(+/- 72.17)**	250<br>250<br>500<br>**333.33**<br>**(+/- 144.34)**	1000<br>1000<br>500<br>**833.33**<br>**(+/- 288.68)**	125<br>125<br>125<br>**125**<br>**(+/- 0)**	250<br>250<br>500<br>**333.33**<br>**(+/- 144.34)**	0.24**<br>0.12**<br>0.24**<br>**0.20****<br>**(+/- 0.07)**	na	na	na
	Candida krusei ATCC 14243	500<br>500<br>250<br>**416.67**<br>**(+/- 144.34)**	500<br>500<br>500<br>**500**<br>**(+/- 0)**	1000<br>1000<br>500<br>**833.33**<br>**(+/- 288.68)**	250<br>250<br>500<br>**333.33**<br>**(+/- 144.34)**	500<br>500<br>1000<br>**666.67**<br>**(+/- 288.68)**	0.24**<br>0.12**<br>0.24**<br>**0.20****<br>**(+/- 0.07)**	na	na	na
	Candida auris CDC 311903	500<br>500<br>1000<br>**666.67**<br>**(+/- 288.68)**	500<br>500<br>250<br>**416.67**<br>**(+/- 144.34)**	1000<br>1000<br>1000<br>**1000**<br>**(+/- 0)**	500<br>500<br>250<br>**416.67**<br>**(+/- 144.34)**	500<br>500<br>500<br>**500**<br>**(+/- 0)**	0.48**<br>0.24**<br>0.98**<br>**0.57****<br>**(+/- 0.38)**	na	na	na

The standard antimicrobial agents used as positive controls: ciprofloxacin (CIP), nitrofurantoin (NIT), cefuroxime (CFX), ampicillin (APC) for bacteria (except enterococci), vancomycin (VA*) for enterococci and nystatin (NY**) for fungi; "-", no activity; nd, not determined, na, not applicable. The mean values with standard deviation (+/- SD) are marked with bolded font.

References:

1. European Committee for Antimicrobial Susceptibility Testing (EUCAST) Determination of minimum inhibitory concentrations (MICs) of antibacterial agents by broth dilution. EUCAST discussion document E. Dis 5.1, *Clin. Microbiol. Infect.* **2003**, 9 ,1–7
2. Clinical and Laboratory Standards Institute. Reference method for broth dilution antifungal susceptibility testing of yeasts. M27-S4. Clinical and Laboratory Standards Institute, Wayne, PA, USA, **2012**
3. Biernasiuk, A.; Kawczyńska, M.; Berecka-Rycerz, A.; Rosada, B.; Gumieniczek, A.; Malm, A.; Dzitko, K.; Łączkowski, K.Z. Synthesis, antimicrobial activity, and determination of the lipophilicity of ((cyclohex-3-enylmethylene)hydrazinyl)thiazole derivatives, *Med. Chem. Res.* **2019**, 28(11), 2023–2036
4. Wiegand, I.; Hilpert, K.; Hancock, R.E.W. Agar and broth dilution methods to determine the minimal inhibitory concentration (MIC) of antimicrobial substances. *Nat. Protoc.* **2008**, 3(2), 163–175
5. O'Donnell, F.; Smyth, T.J.; Ramachandran, V.N.; Smyth, W.F. A study of the antimicrobial activity of selected synthetic and naturally occurring quinolines. *Int. J. Antimicrob. Agents* **2010**, 35, 30–38

5. Cytotoxicity

Table S9. The cell proliferation in % (mean and standard deviation ± SD) after 24h and 48h exposition on the complexes **1** – **5** in L929 cell line (results are presented as % of viable cells)

**Complex No / Concentration (µM)**	**24h**					**48h**				
	**1**	**2**	**3**	**4**	**5**	**1**	**2**	**3**	**4**	**5**
**Control**	89±0.66	97±0.25	88±0.33	92±0.42	89±0.82	101±0.41	86±0.38	92±0.35	101±0.88	94±0.28
**5**	95±0.42	102±0.16	99±0.52	95±0.62	96±0.46	97±0.78	106±0.92	109±0.48	98±0.75	96±0.77
**10**	95±0.78	93±0.78	96±0.69	89±0.55	99±0.73	106±0.56	107±0.44	103±0.57	91±0.32	96±0.38
**25**	88±0.65	93±0.89	89±0.21	61±0.68	89±0.59	101±0.35	97±0.70	81±0.25	92±0.66	94±0.17
**50**	89±0.25	83±0.24	69±0.36	72±0.42	89±0.12	86±0.52	78±0.25	89±0.65	67±0.71	85±0.89
**100**	72±0.15	69±0.45	78±0.70	79±0.45	67±0.37	78±0.65	81±0.42	86±0.42	75±0.45	77±0.28
**150**	81±0.18	84±1.07	98±0.50	91±0.32	88±0.78	79±0.80	94±0.67	92±0.36	86±0.62	88±0.60
**200**	76±0.78	79±0.56	102±0.83	89±0.66	85±0.14	73±0.22	78±0.34	84±0.88	80±0.34	97±0.55

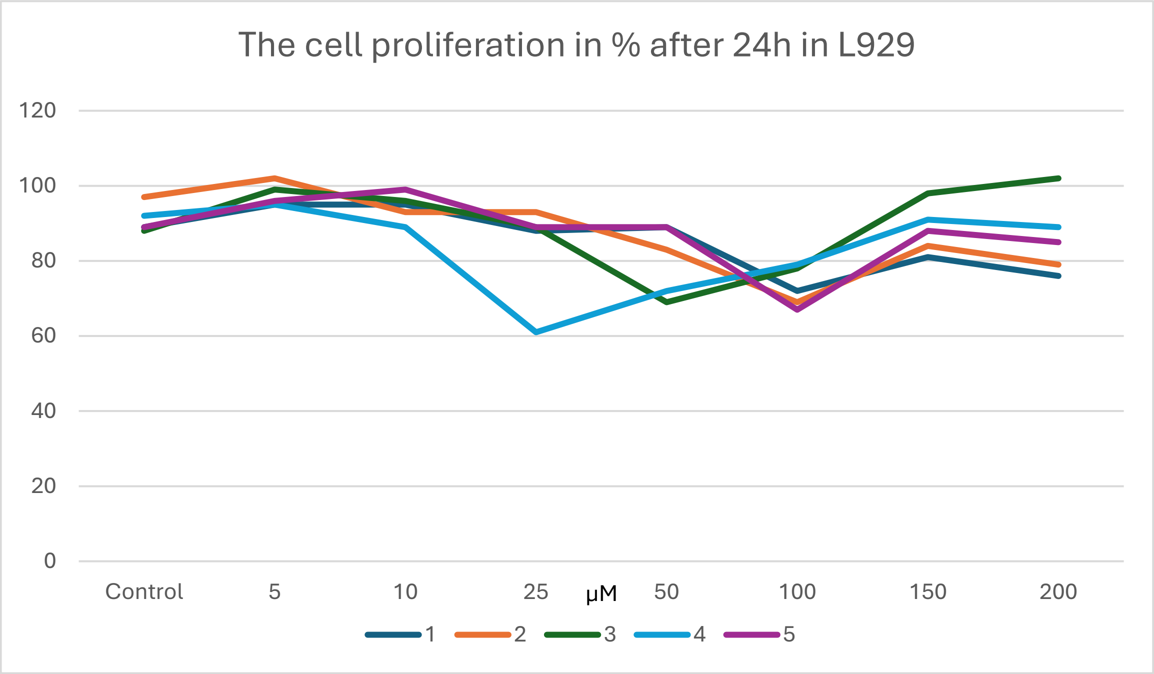

Figure S10. The cell proliferation in % after 24h exposition on the complexes **1** – **5** in L929 cell line

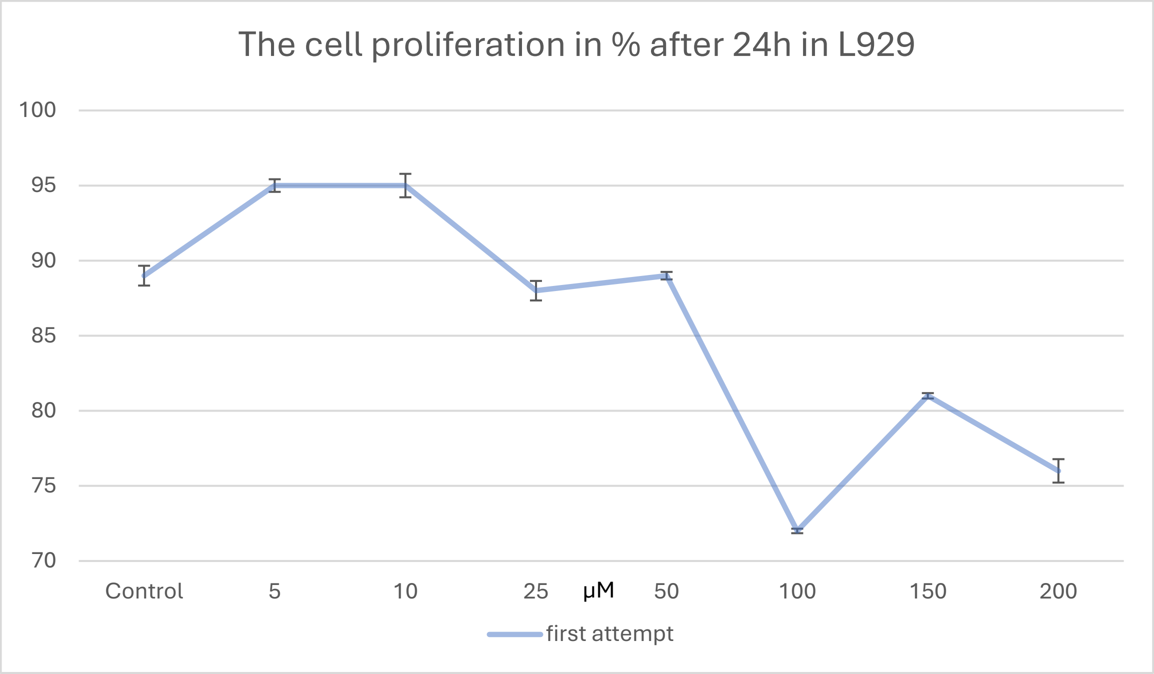

Figure S11. The cell proliferation in % after 24h exposition on the complex **1** in L929 cell line

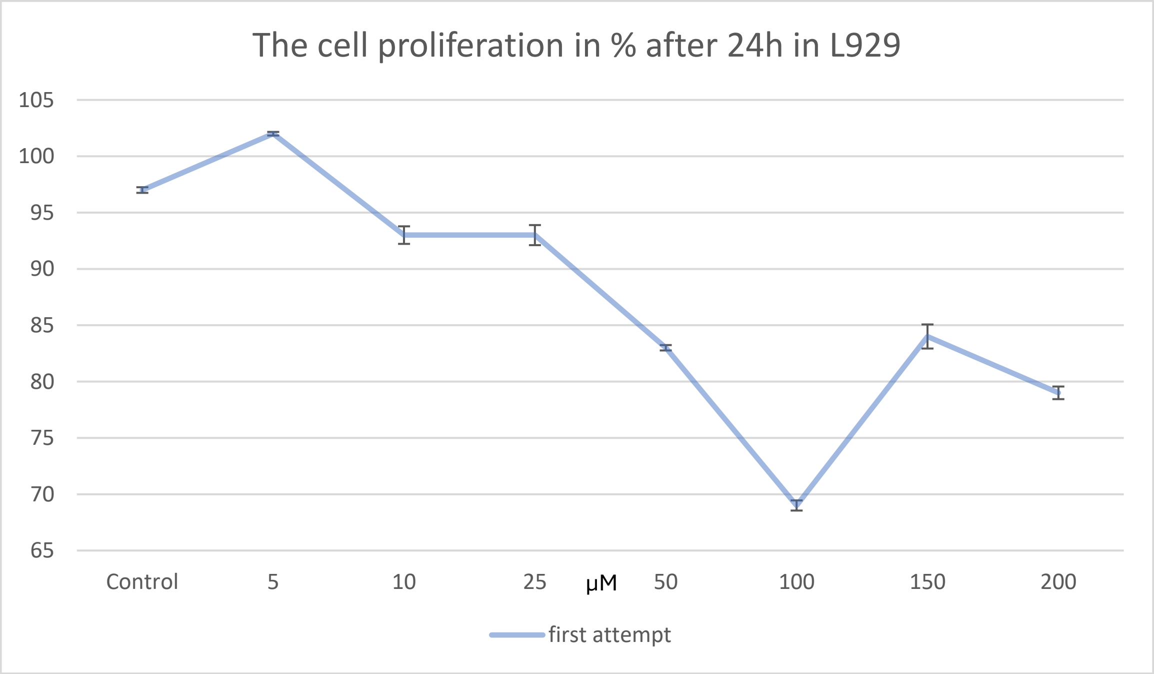

Figure S12. The cell proliferation in % after 24h exposition on the complex **2** in L929 cell line

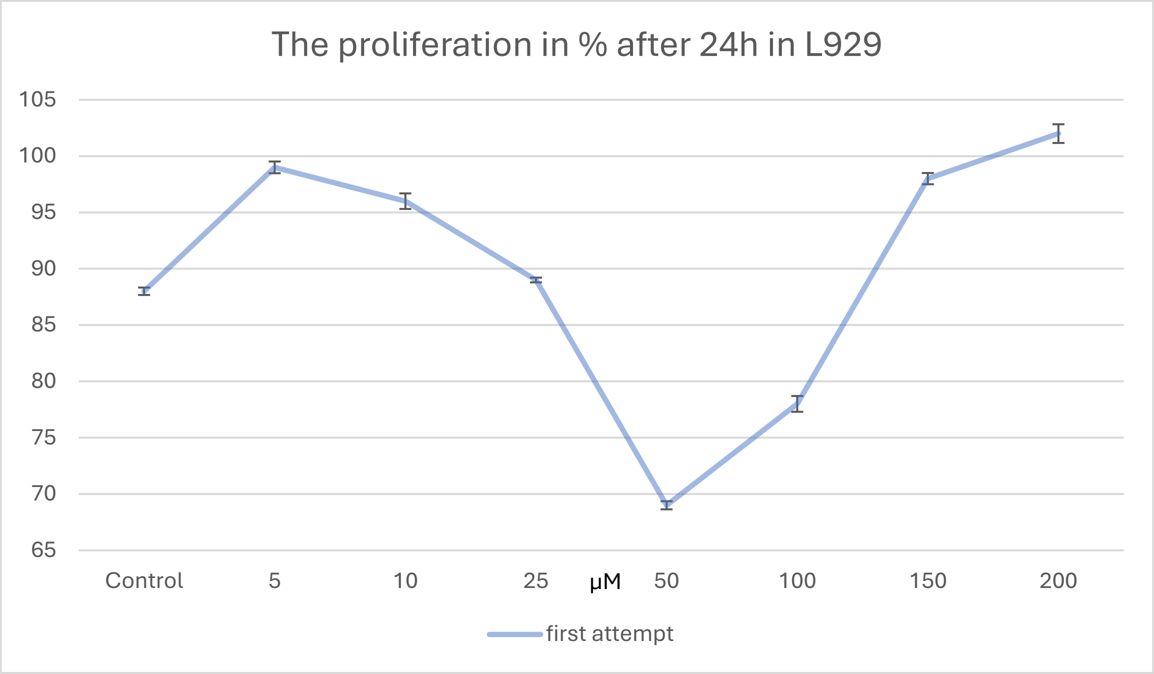

Figure S13. The cell proliferation in % after 24h exposition on the complex **3** in L929 cell line

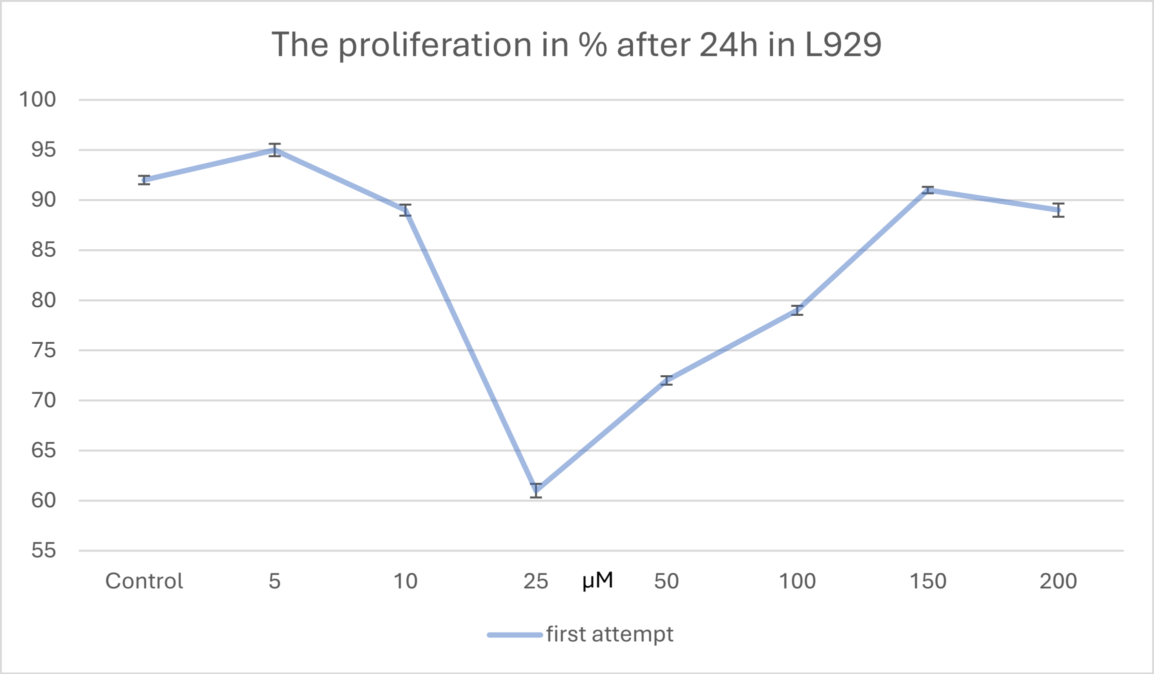

Figure S14. The cell proliferation in % after 24h exposition on the complex **4** in L929 cell line

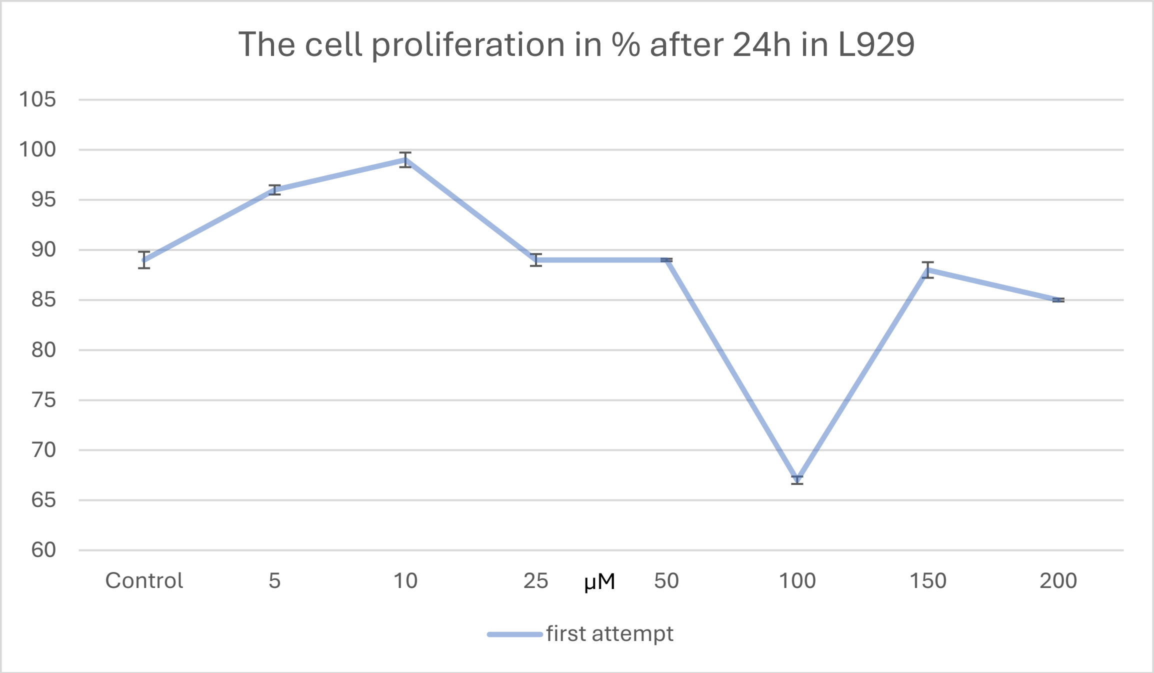

Figure S15. The cell proliferation in % after 24h exposition on the complex **5** in L929 cell line

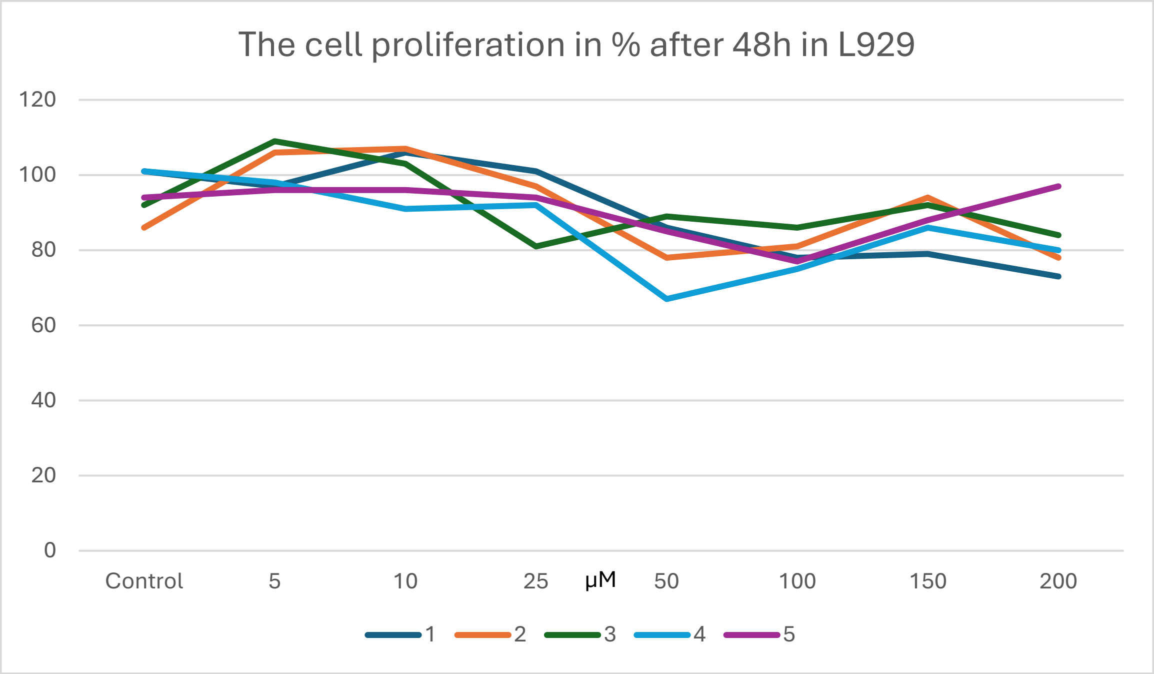

Figure S16. The cell proliferation in % after 48h exposition on the complexes **1** – **5** in L929 cell line

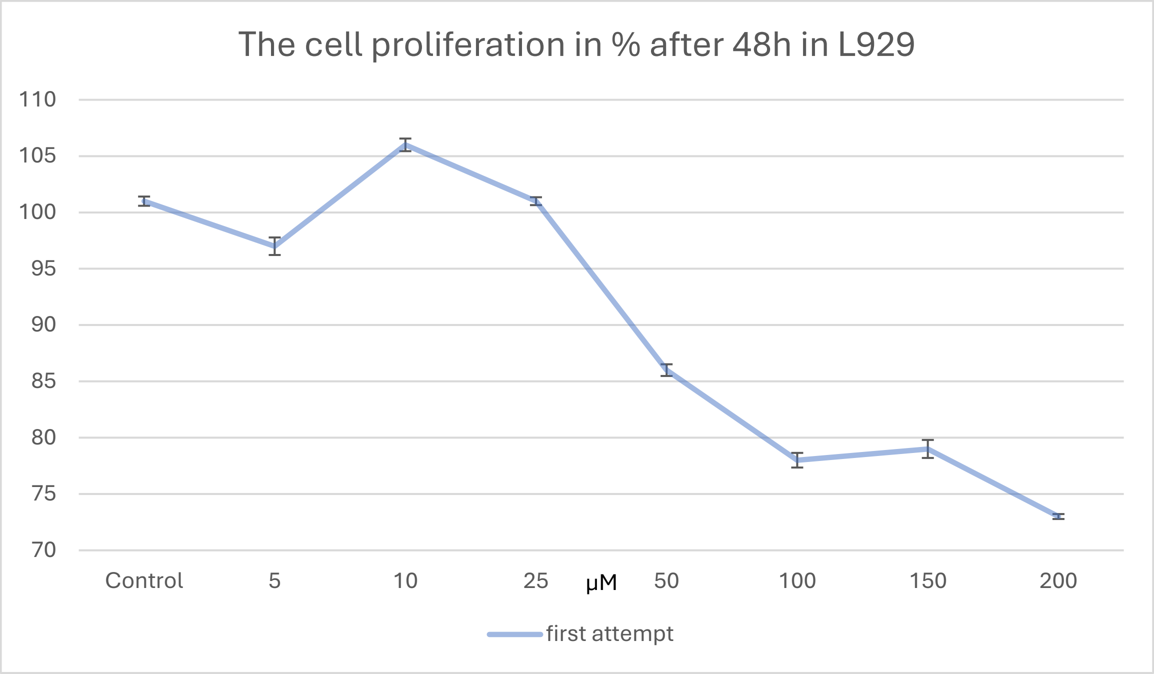

Figure S17. The cell proliferation in % after 48h exposition on the complex **1** in L929 cell line

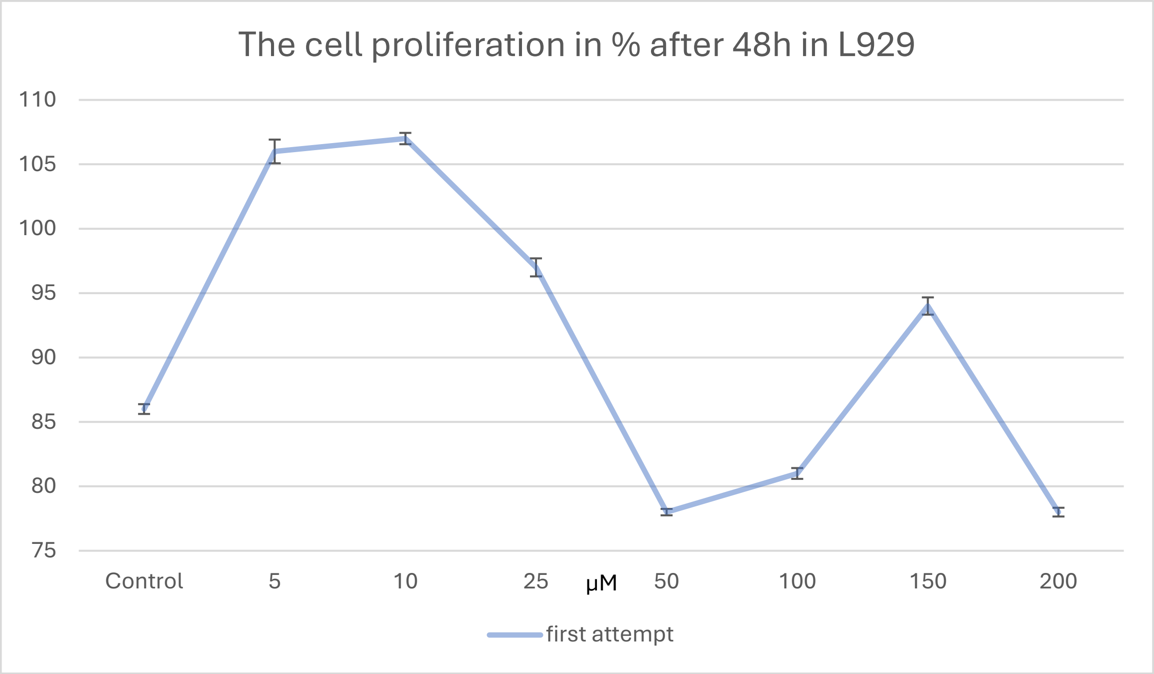

Figure S18. The cell proliferation in % after 48h exposition on the complex **2** in L929 cell line

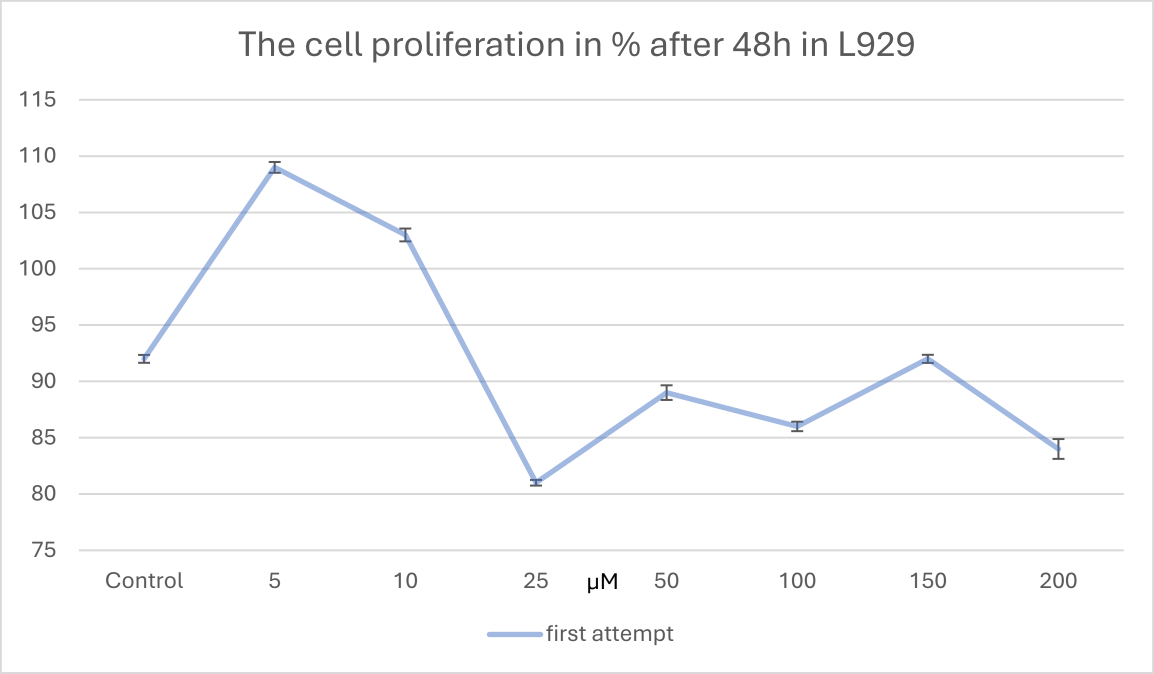

Figure S19. The cell proliferation in % after 48h exposition on the complex **3** in L929 cell line

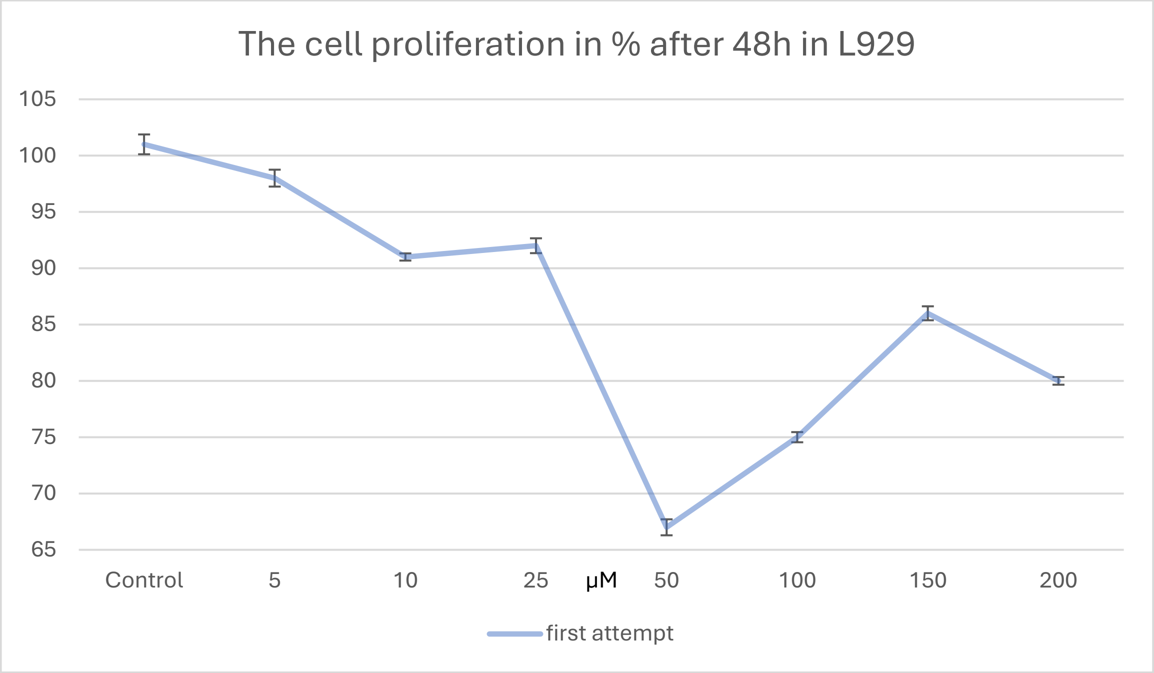

Figure S20. The cell proliferation in % after 48h exposition on the complex **4** in L929 cell line

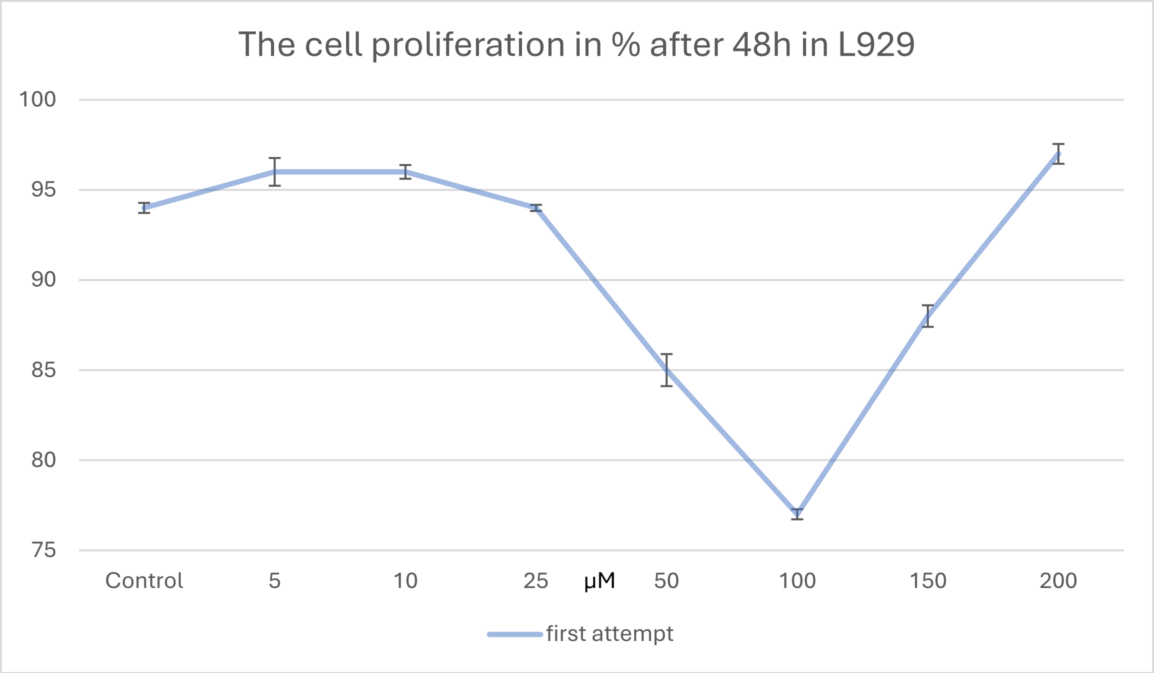

Figure S21. The cell proliferation in % after 48h exposition on the complex **5** in L929 cell line

Table S10. The cell proliferation in % (mean and standard deviation ± SD) after 24h and 48h exposition on the all complexes **1** – **5** in T47D cell line (results are presented as % of viable cells)

Complex No / Concentration (µM)	24h					48h				
	1	2	3	4	5	1	2	3	4	5
**Control**	101±0.68	99±0.28	96±0.18	104±0.64	97±0.66	95±0.73	88±0.9	107±0.12	99±0.44	103±0.34
**5**	93±0.72	95±0.68	99±0.29	97±0.90	89±0.28	87±0.81	89±0.21	97±0.32	96±0.38	98±0.29
**10**	95±0.54	88±0.29	87±0.35	95±0.54	102±0.33	93±0.32	85±0.27	89±0.68	91±0.78	93±0.47
**25**	98±0.58	97±0.30	77±0.78	87±0.82	81±0.91	87±0.45	81±0.76	77±0.54	76±1.03	86±0.75
**50**	78±0.15	89±0.28	72±0.65	76±0.42	84±0.32	86±0.67	79±0.48	71±0.24	78±0.88	88±0.50
**100**	76±0.28	87±0.45	77±0.28	66±0.53	69±0.59	61±0.34	72±0.61	70±0.11	63±0.62	64±0.28
**150**	74±1.02	64±0.66	72±0.15	64±1.06	65±0.52	79±0.58	75±0.53	72±0.46	69±0.55	67±0.13
**200**	63±0.69	69±0.35	74±0.32	67±0.26	59±0.43	78±0.60	67±0.75	69±0.18	64±0.28	65±0.45

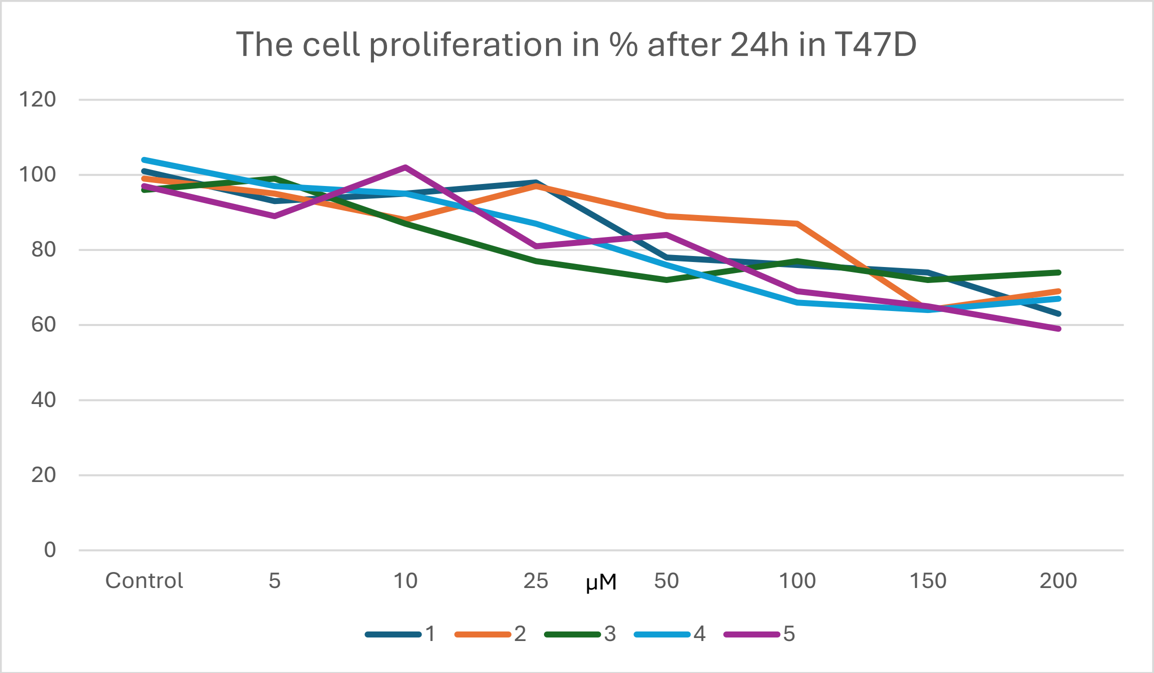

Figure S22. The cell proliferation in % after 24h exposition on the all complexes **1** – **5** in T47D cell line

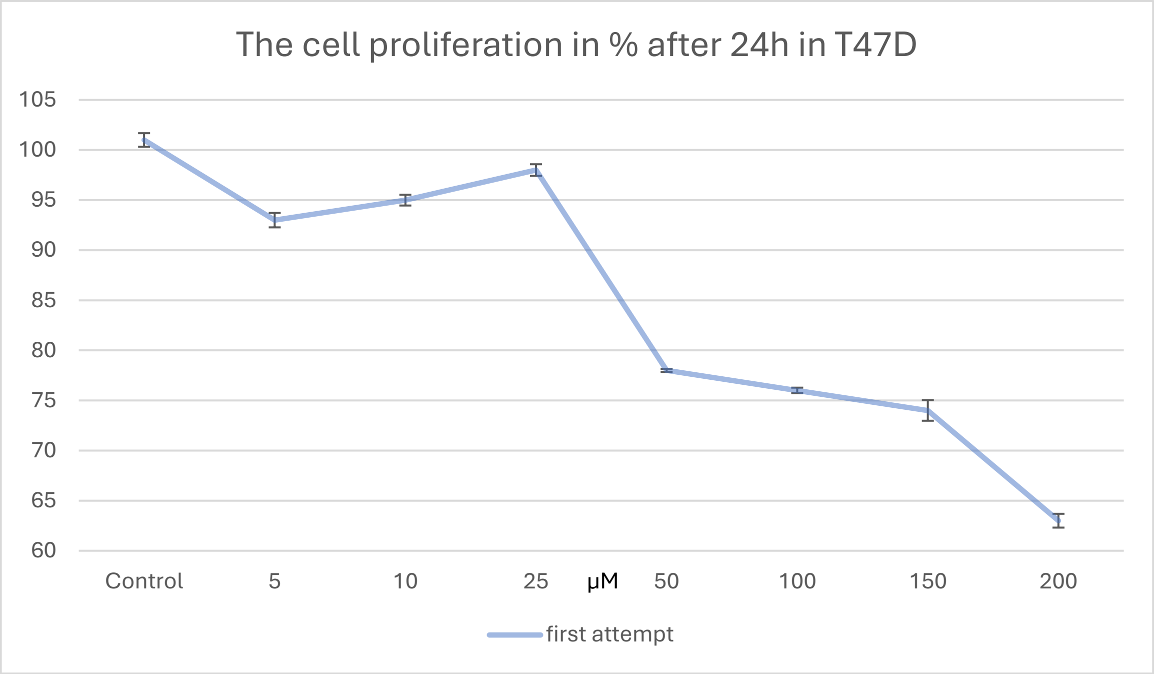

Figure S23. The cell proliferation in % after 24h exposition on the complex **1** in T47D cell line

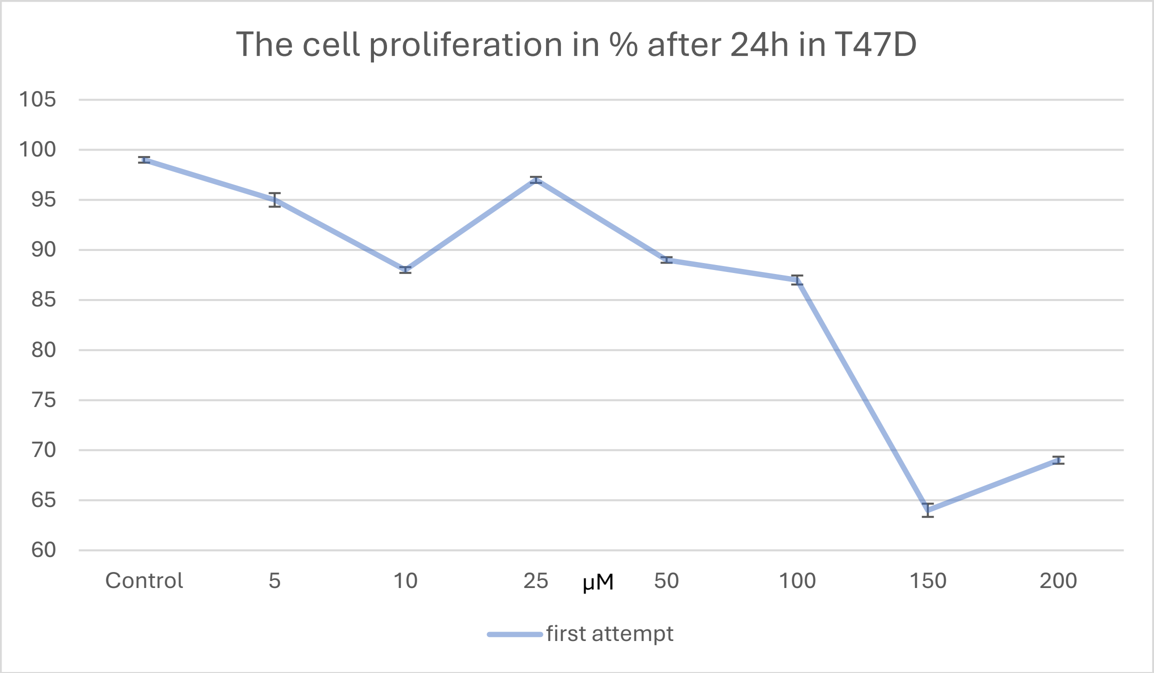

Figure S24. The cell proliferation in % after 24h exposition on the complex **2** in T47D cell line

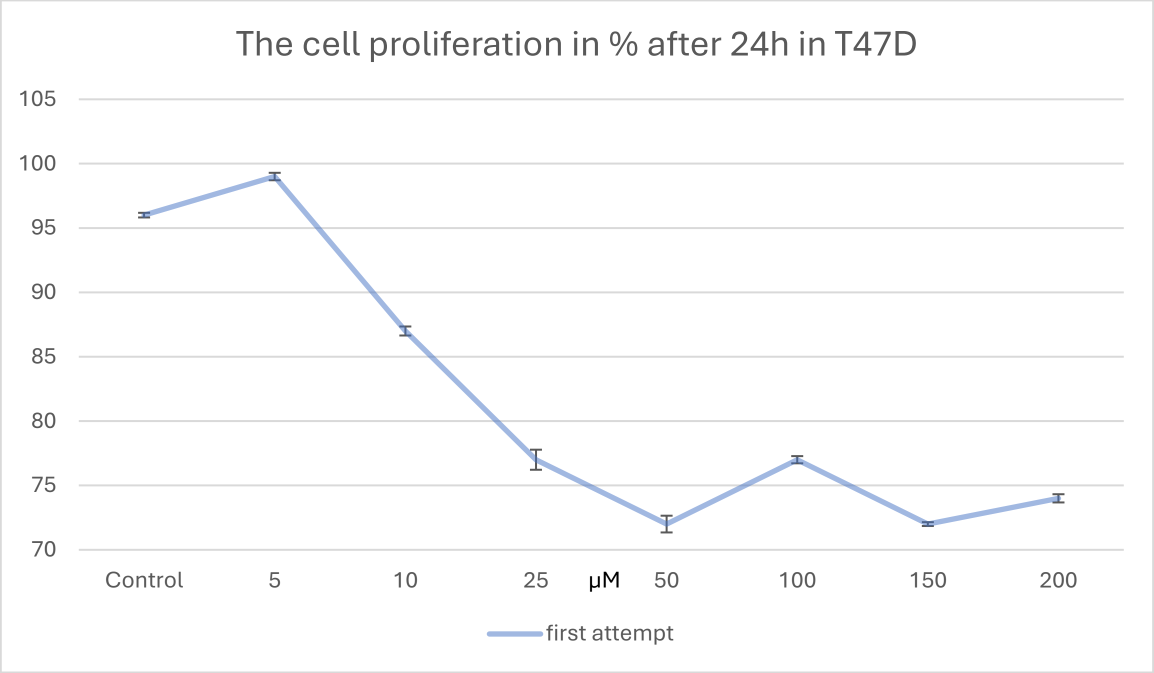

Figure S25. The cell proliferation in % after 24h exposition on the complex **3** in T47D cell line

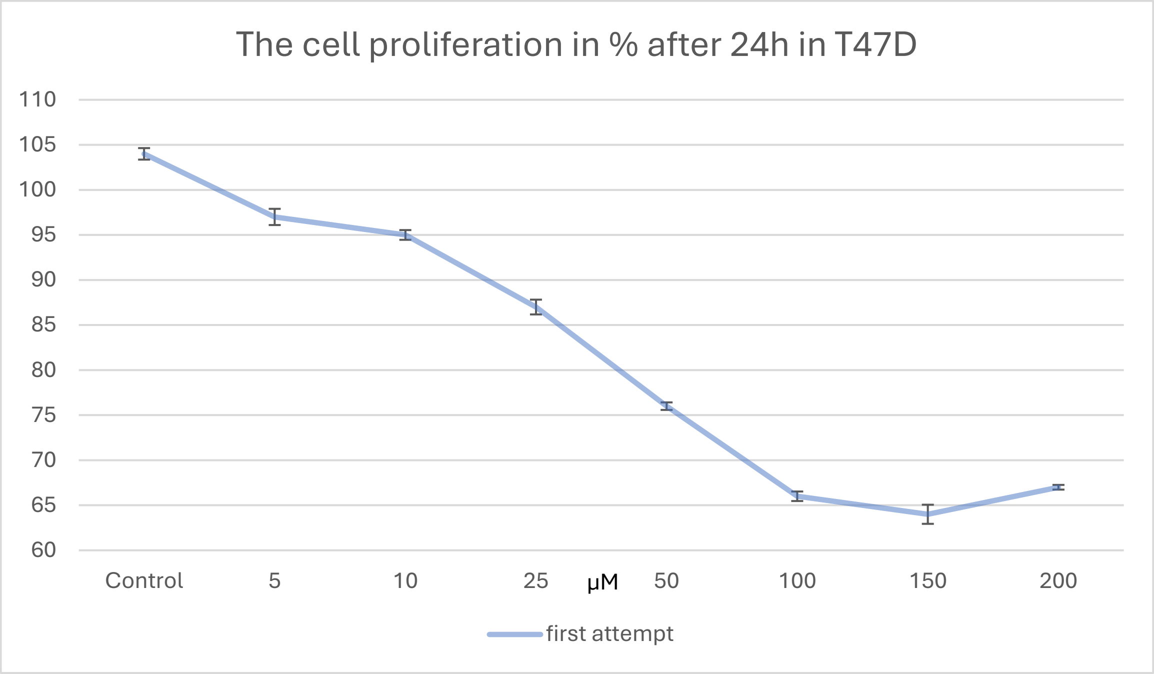

Figure S26. The cell proliferation in % after 24h exposition on the complex **4** in T47D cell line

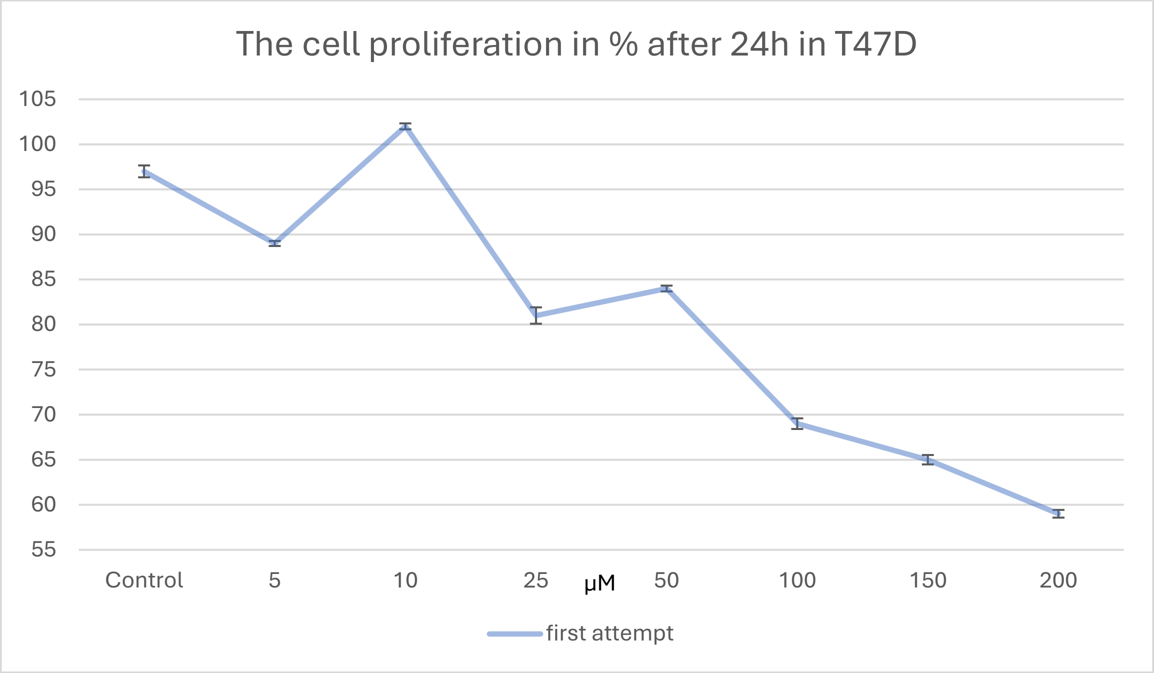

Figure S27. The cell proliferation in % after 24h exposition on the complex **5** in T47D cell line

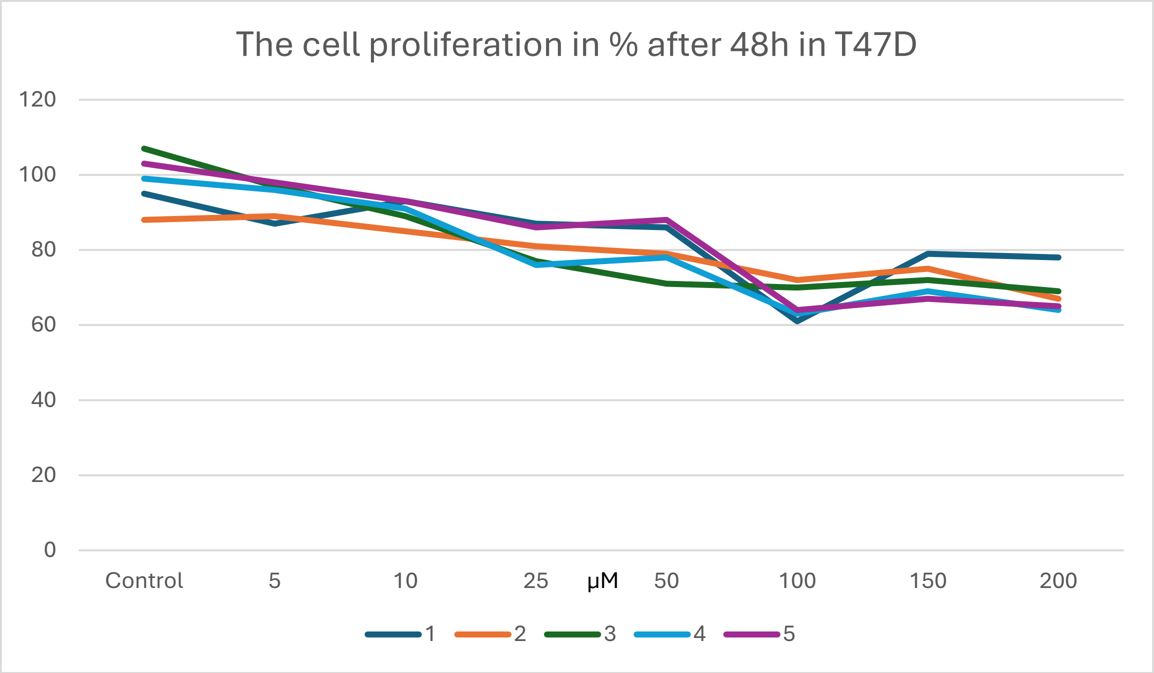

Figure S28. The cell proliferation in % after 48h exposition on the all complexes **1** – **5** in T47D cell line

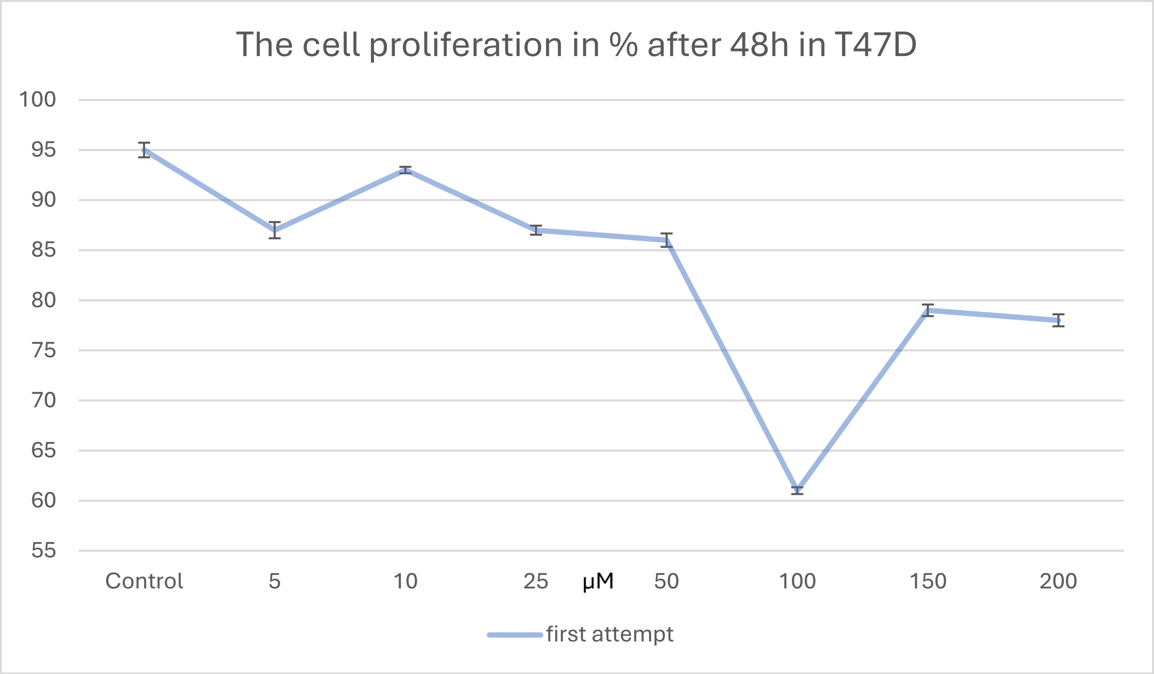

Figure S29. The cell proliferation in % after 48h exposition on the complex **1** in T47D cell line

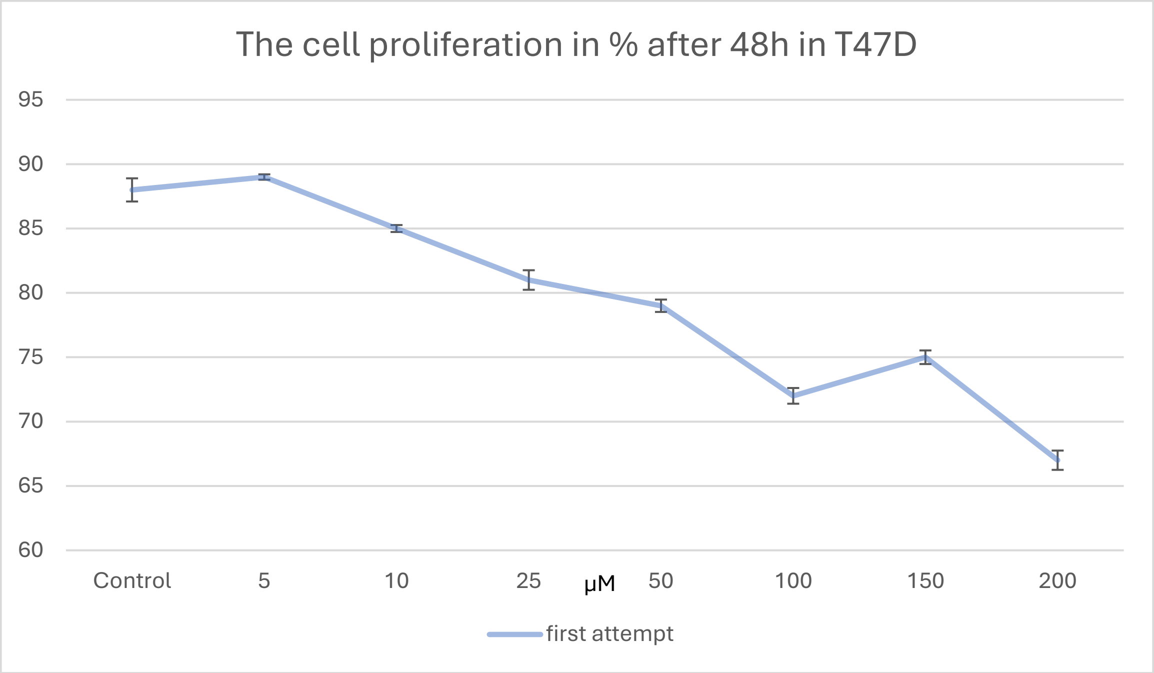

Figure S30. The cell proliferation in % after 48h exposition on the complex **2** in T47D cell line

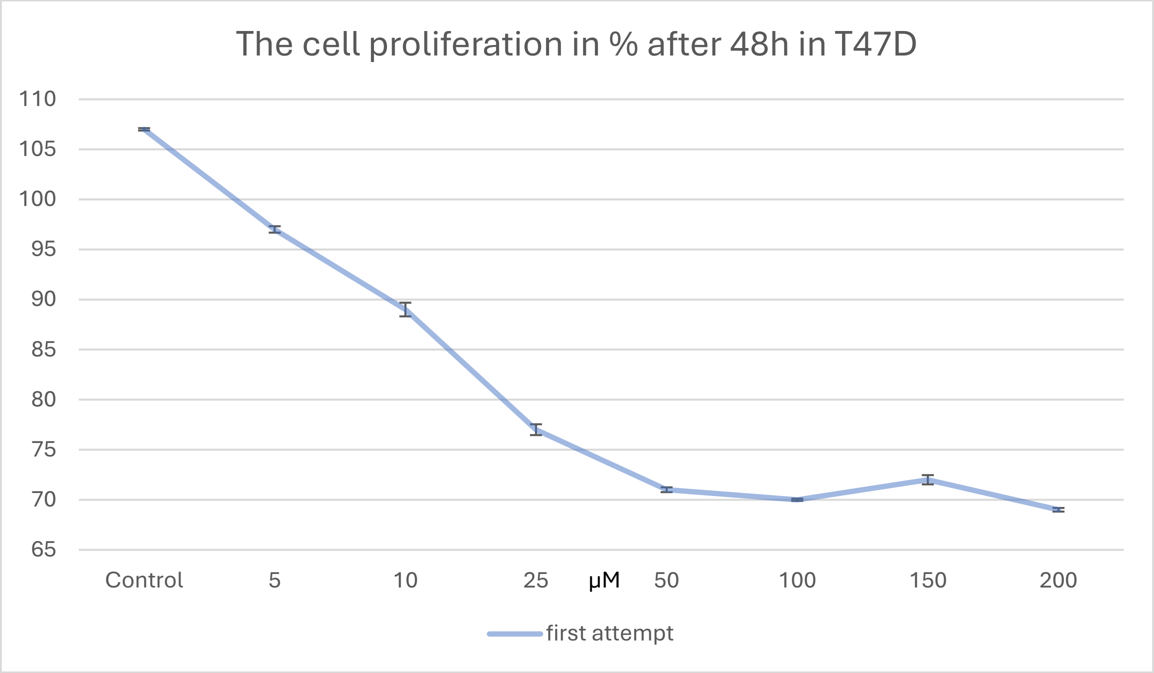

Figure S31. The cell proliferation in % after 48h exposition on the complex **3** in T47D cell line

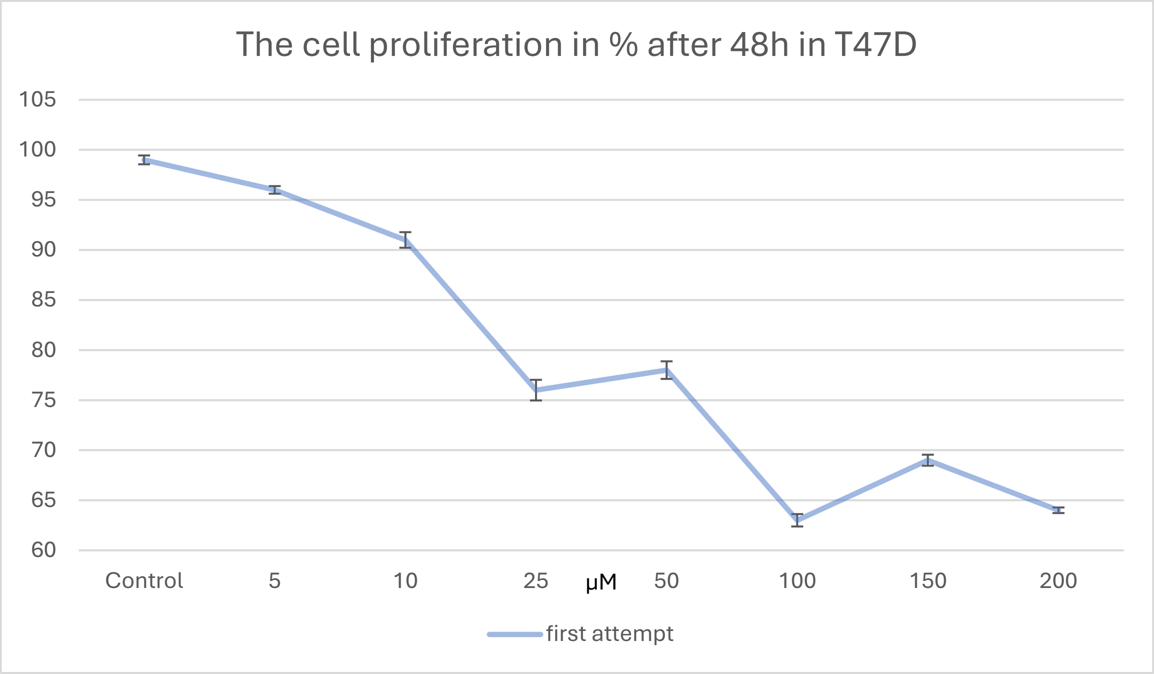

Figure S32. The cell proliferation in % after 48h exposition on the complex **4** in T47D cell line

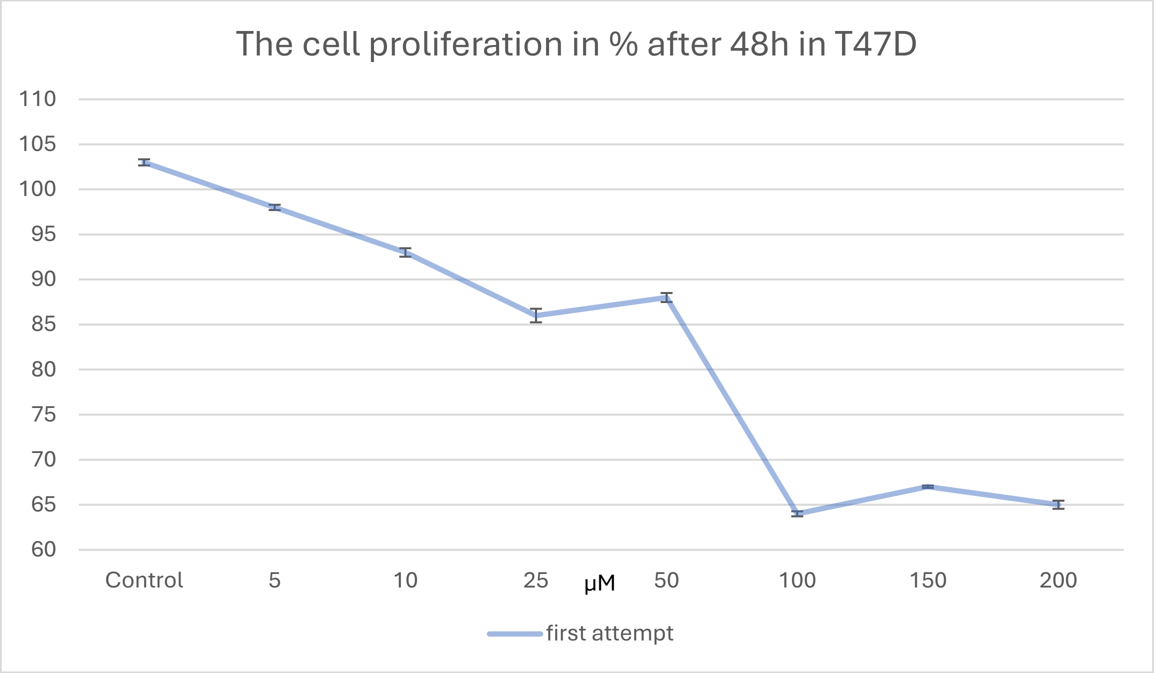

Figure S33. The cell proliferation in % after 48h exposition on the complex **5** in T47D cell line

Table S11. The cell proliferation in % (mean and standard deviation ± SD) after 24h and 48h exposition on the all complexes **1** – **5** in A549 cell line (results are presented as % of viable cells)

Complex No/ Concentration (μM)	24h					48h				
	1	2	3	4	5	1	2	3	4	5
**Control**	99±0.68	104±0.39	92±0.45	94±0.18	95±0.37	96±0.56	108±0.25	99±0.34	104±0.77	96±0.55
**5**	101±0.56	98±0.40	87±0.22	88±0.60	98±0.26	96±0.40	101±0.58	102±0.72	112±0.67	90±0.81
**10**	78±0.28	77±0.94	101±0.80	96±0.12	88±0.77	87±0.95	89±0.83	90±0.94	91±0.15	100±0.74
**25**	89±0.45	78±1.05	93±0.28	89±0.35	81±0.84	89±1.01	78±0.90	91±0.28	96±0.28	101±0.43
**50**	89±0.68	91±0.35	96±0.66	74±0.69	83±0.79	89±0.39	92±0.48	95±0.37	71±0.30	86±0.67
**100**	76±0.92	69±0.78	72±0.35	77±0.62	66±0.24	65±0.68	81±0.78	77±0.20	76±0.87	83±0.19
**150**	78±0.33	84±0.64	79±0.38	76±0.57	83±0.54	74±0.55	85±0.40	81±0.78	70±0.25	81±0.32
**200**	76±0.27	81±0.44	67±0.15	69±0.30	71±0.52	76±0.32	72±0.25	79±0.30	62±0.60	78±0.91

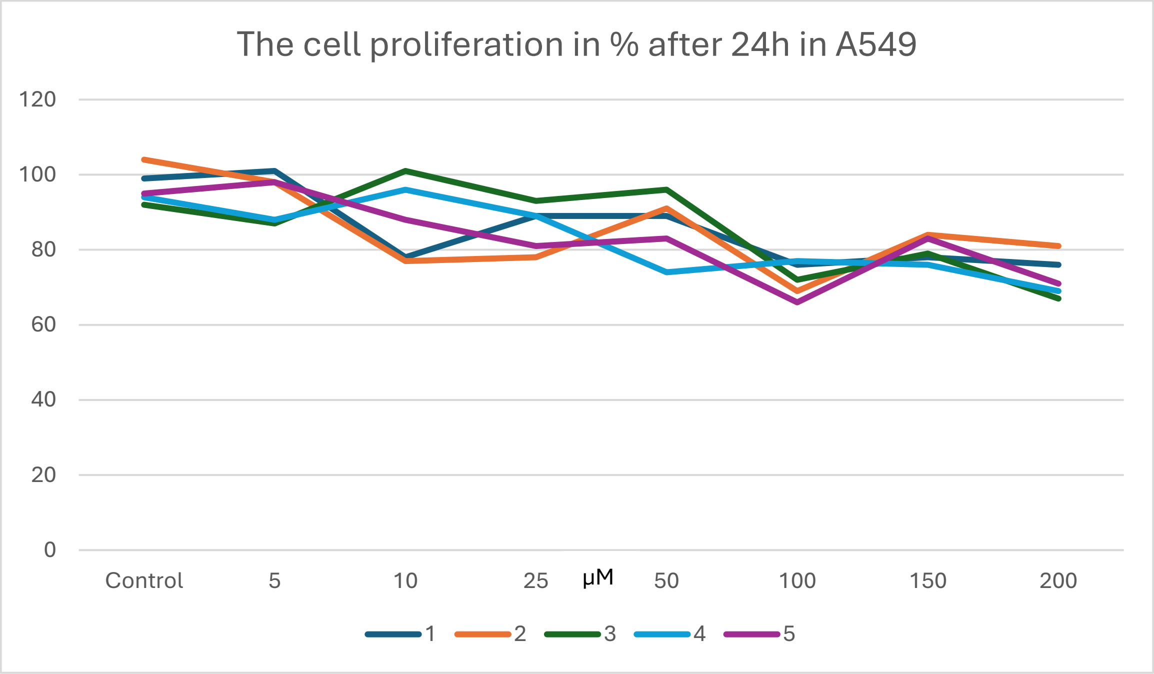

Figure S34. The cell proliferation in % after 24h exposition on the all complexes **1** – **5** in A549 cell line

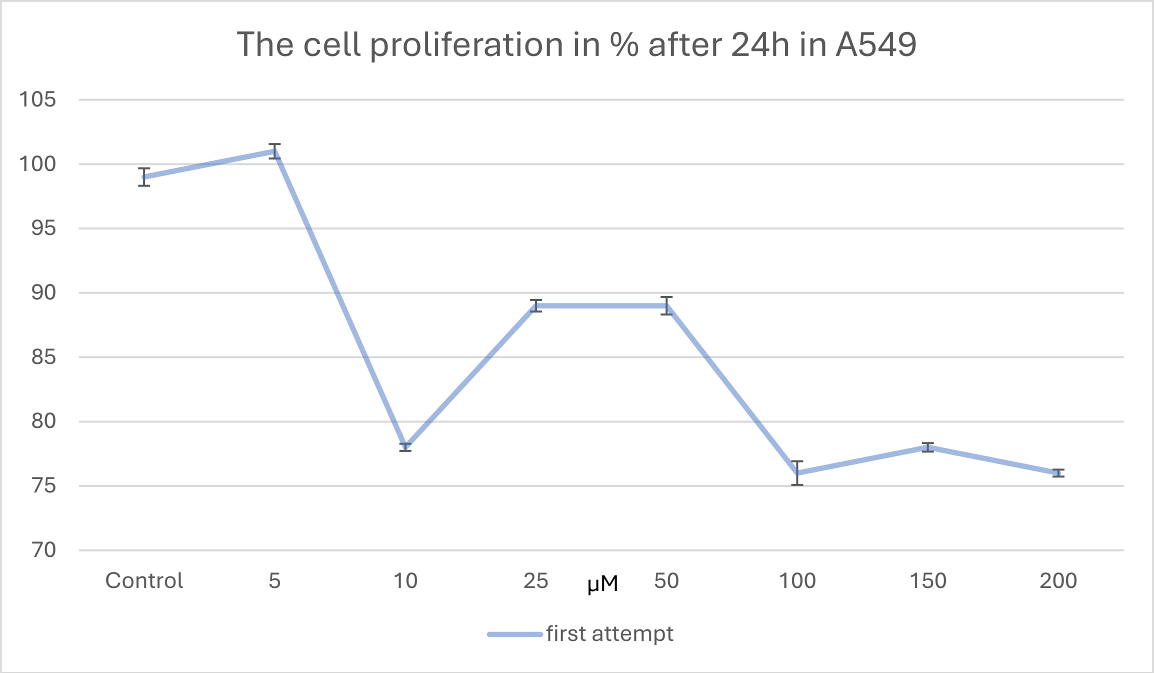

Figure S35. The cell proliferation in % after 24h exposition on the complex **1** in A549 cell line

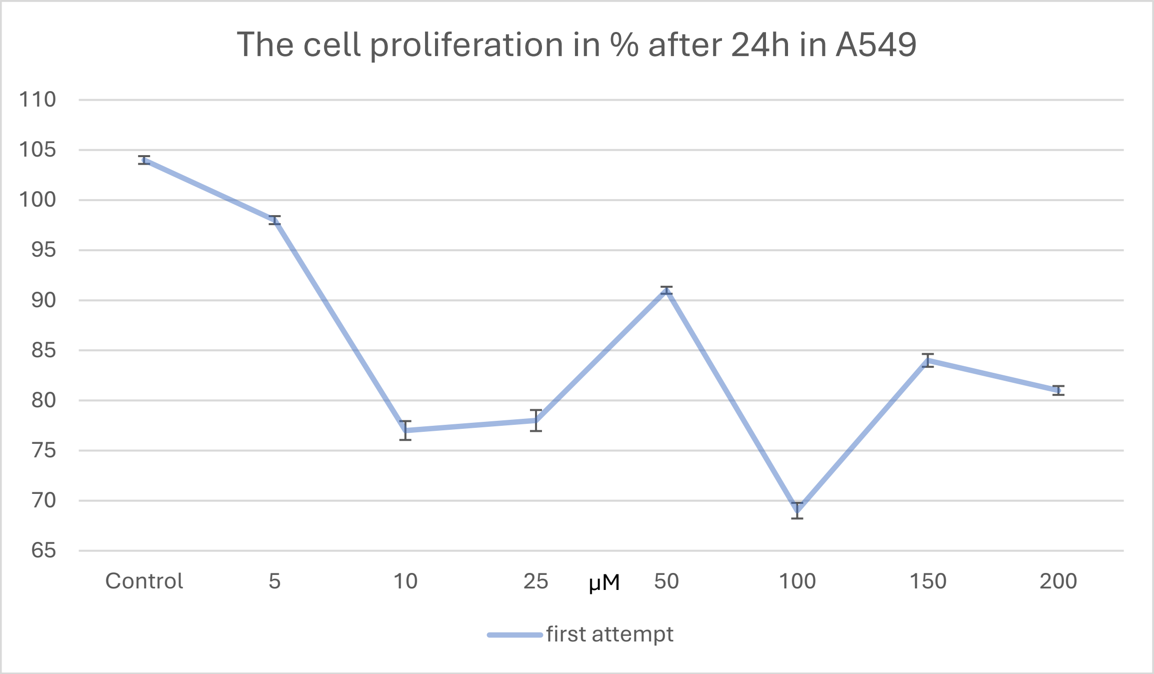

Figure S36. The cell proliferation in % after 24h exposition on the complex **2** in A549 cell line

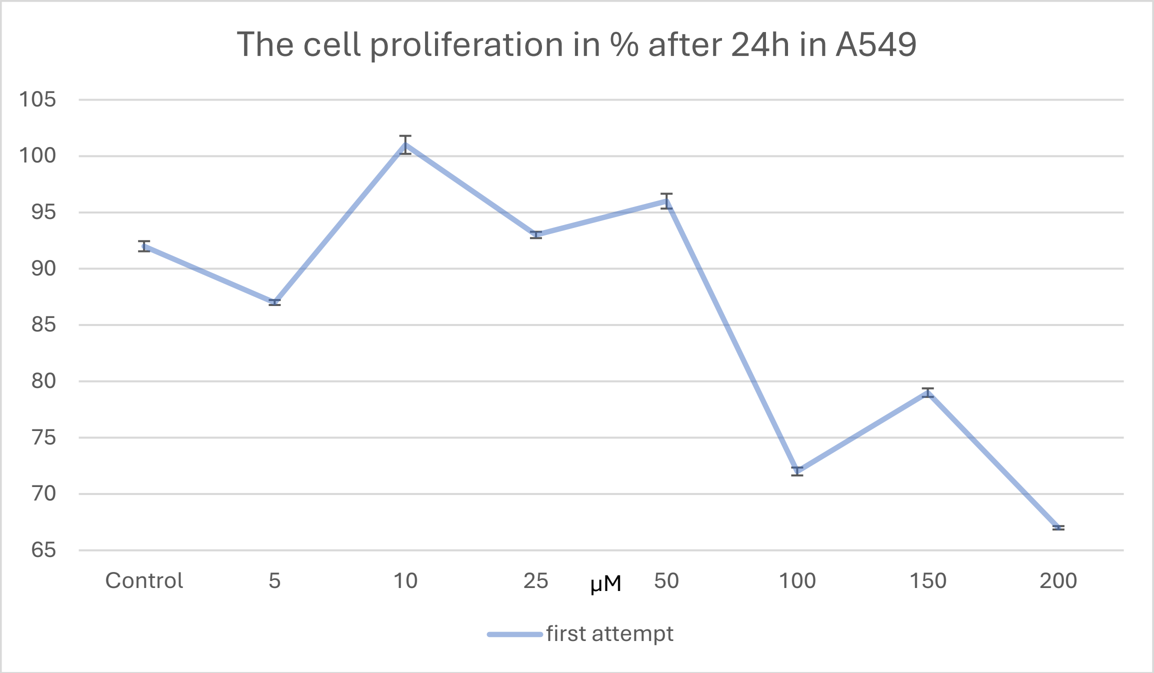

Figure S37. The cell proliferation in % after 24h exposition on the complex **3** in A549 cell line

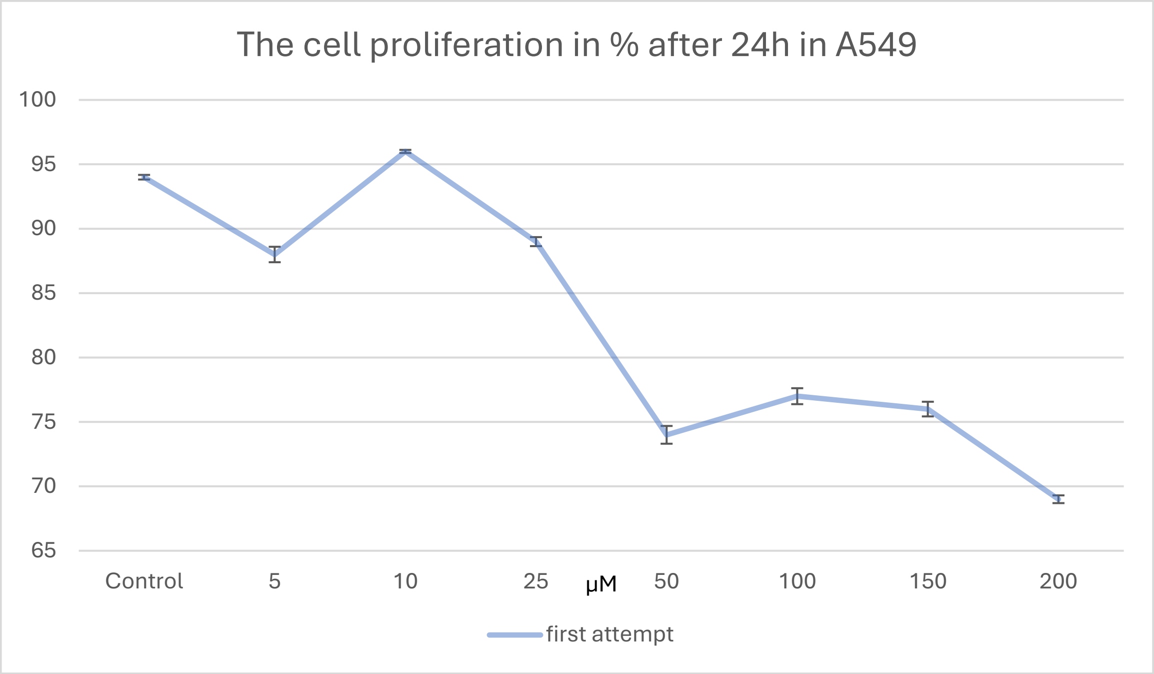

Figure S38. The cell proliferation in % after 24h exposition on the complex **4** in A549 cell line

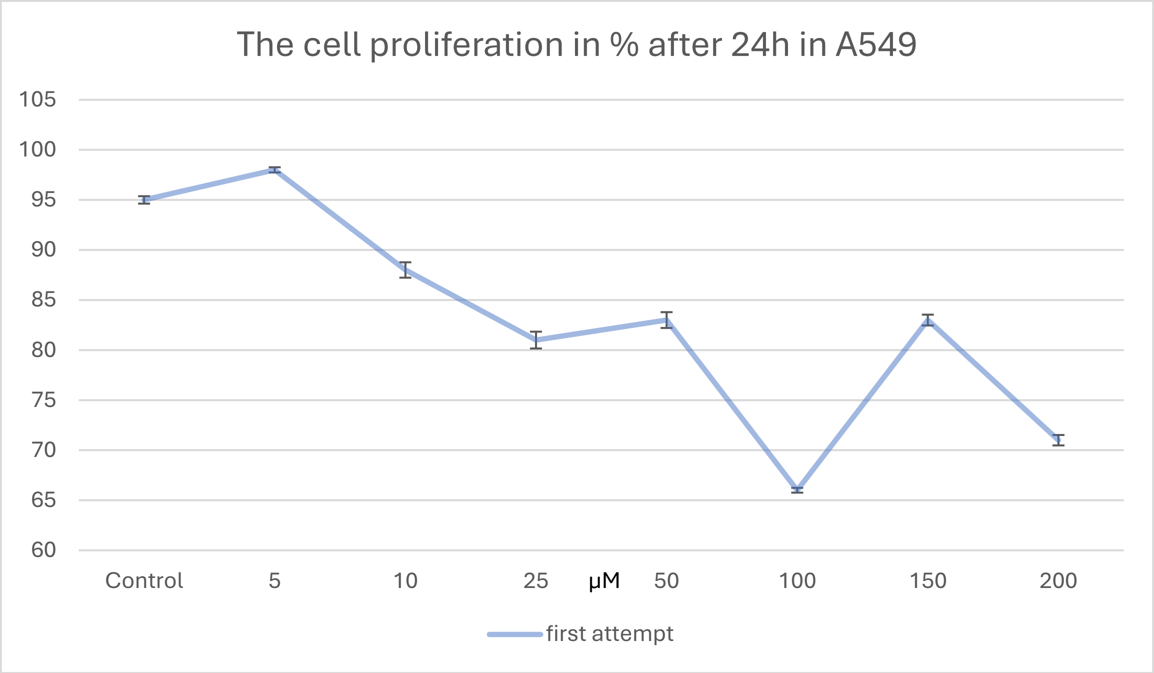

Figure S39. The cell proliferation in % after 24h exposition on the complex **5** in A549 cell line

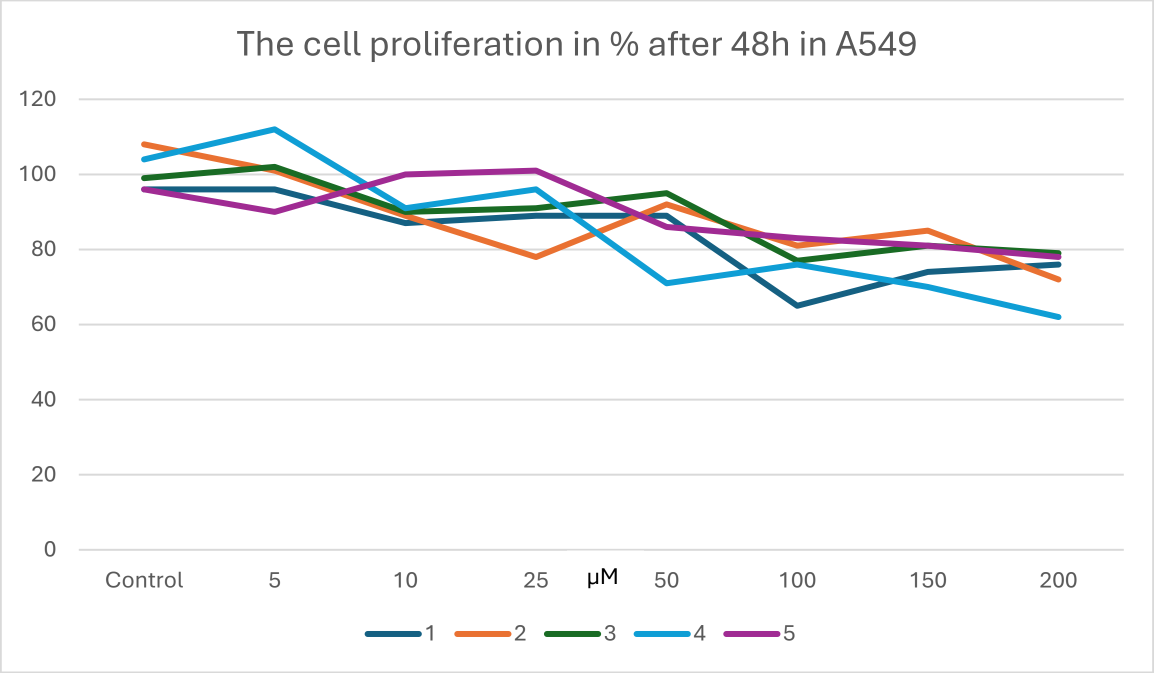

Figure S40. The cell proliferation in % after 48h exposition on the all complexes **1** – **5** in A549 cell line

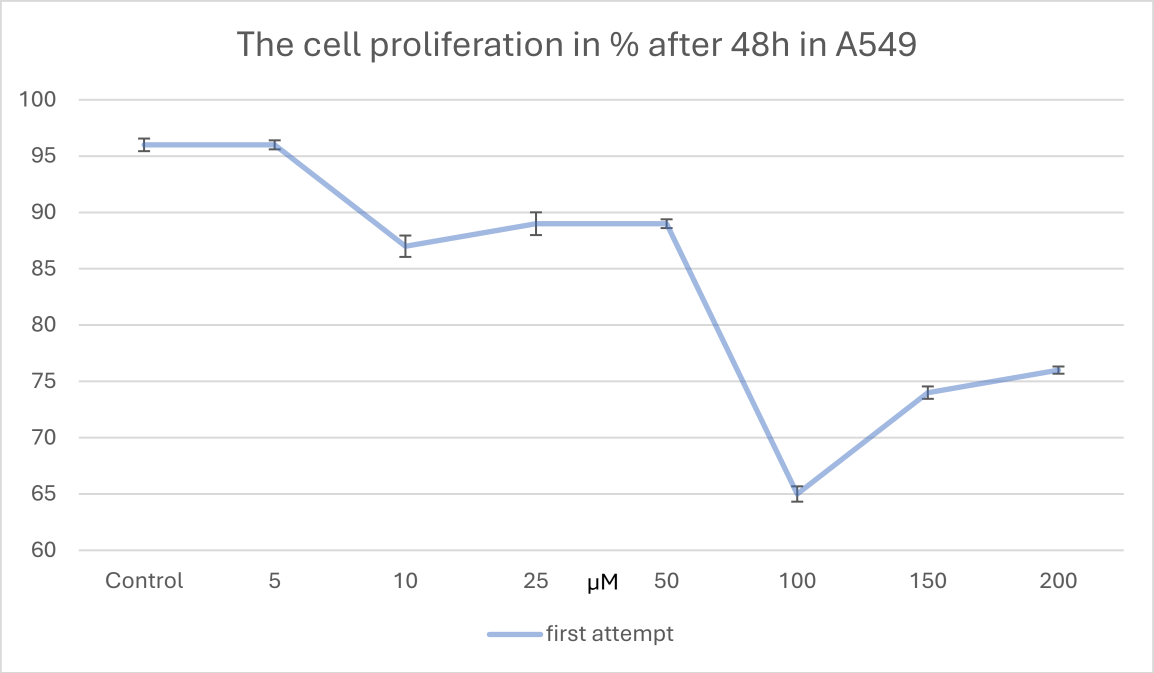

Figure S41. The cell proliferation in % after 48h exposition on the complex **1** in A549 cell line

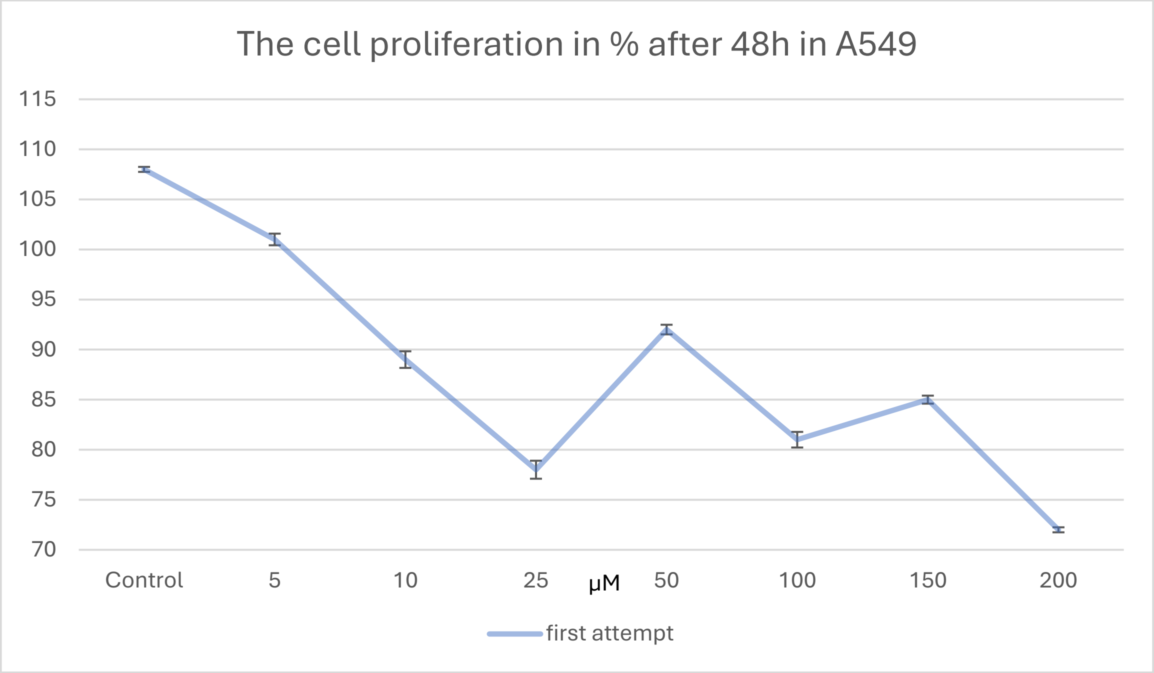

Figure S42. The cell proliferation in % after 48h exposition on the complex **2** in A549 cell line

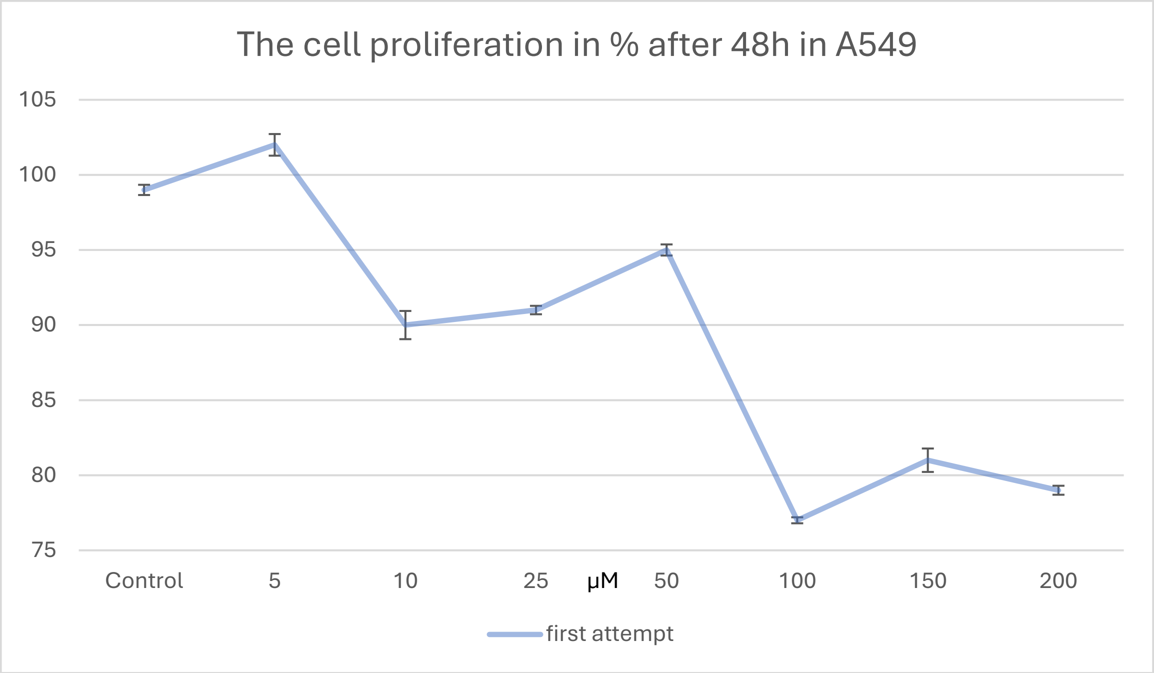

Figure S43. The cell proliferation in % after 48h exposition on the complex **3** in A549 cell line

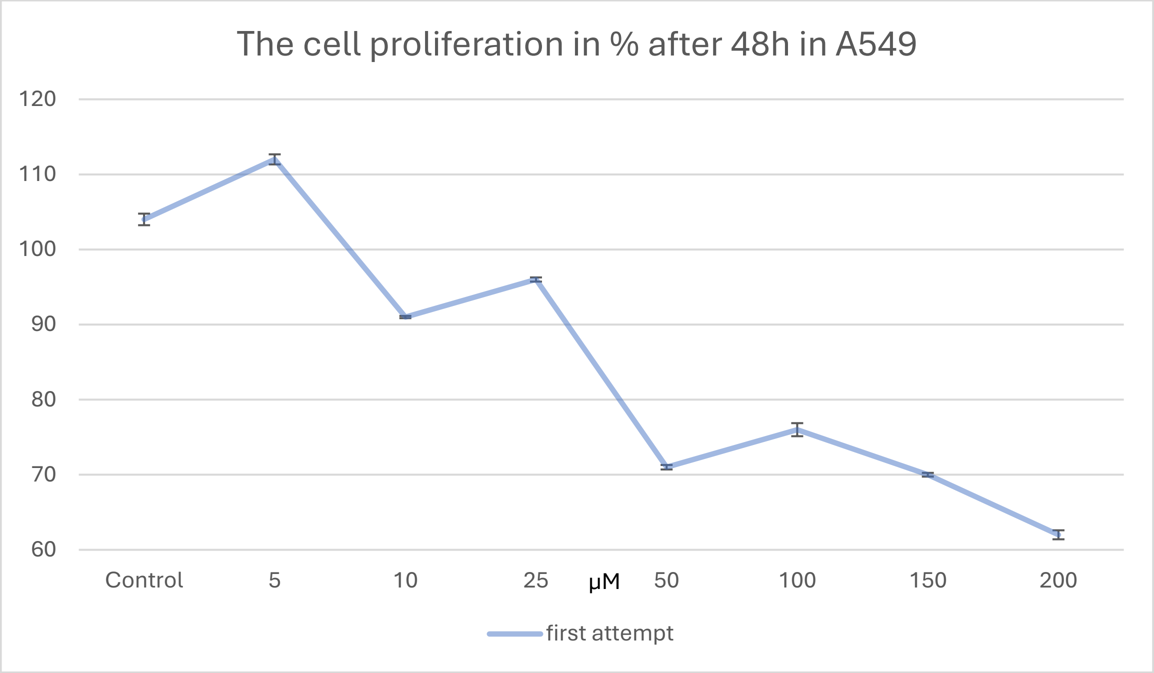

Figure S44. The cell proliferation in % after 48h exposition on the complex **4** in A549 cell line

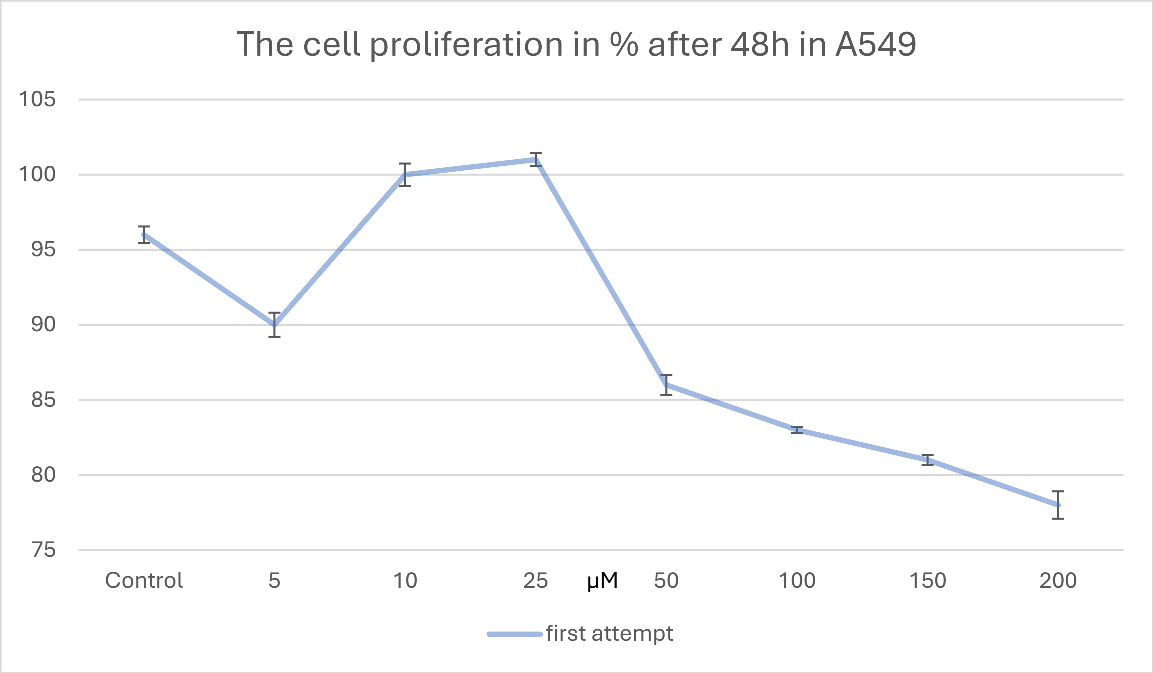

Figure S45. The cell proliferation in % after 48h exposition on the complex **5** in A549 cell line

Table S12. The cell proliferation in % (mean and standard deviation +/- SD) after 24h and 48h exposition on the all complexes **1** – **5** in HeLa cell line (results are presented as % of viable cells)

Complex No/ Concentration (µM)	24h					48h				
	1	2	3	4	5	1	2	3	4	5
**Control**	103±0.94	99±0.68	92±0.89	87±0.90	104±0.47	98±0.56	91±0.26	88±0.45	93±0.66	89±0.50
**5**	101±0.62	89±0.55	78±0.33	85±0.36	89±0.87	104±0.48	85±0.47	71±0.27	87±0.78	84±0.22
**10**	88±0.78	79±0.27	72±0.23	81±0.65	84±0.70	90±0.69	81±0.35	84±0.40	81±0.55	89±0.71
**25**	91±0.47	84±0.69	69±0.79	72±0.48	79±0.34	76±0.78	87±0.23	72±0.38	79±0.35	87±0.56
**50**	78±0.26	74±0.45	63±0.51	62±0.21	81±0.27	70±0.35	73±0.48	61±0.73	64±0.71	76±0.29
**100**	62±0.77	60±0.58	56±0.62	68±0.43	64±0.67	58±0.89	76±0.58	58±0.50	59±0.53	63±0.73
**150**	64±0.15	82±1.02	80±0.44	91±0.73	84±0.45	66±0.25	87±0.57	75±0.66	86±0.68	80±0.49
**200**	63±0.20	78±0.44	71±0.56	69±0.24	85±0.42	69±0.14	64±0.30	79±0.32	80±0.45	66±0.52

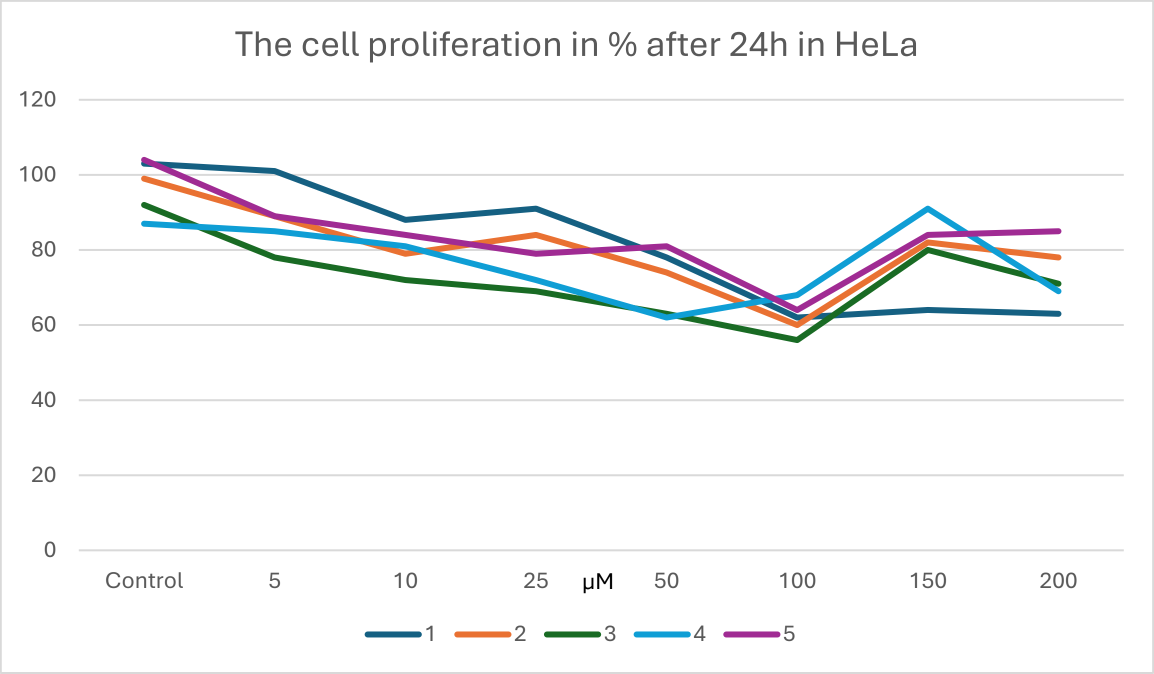

Figure S46. The cell proliferation in % after 24h exposition on the all complexes **1** – **5** in HeLa cell line

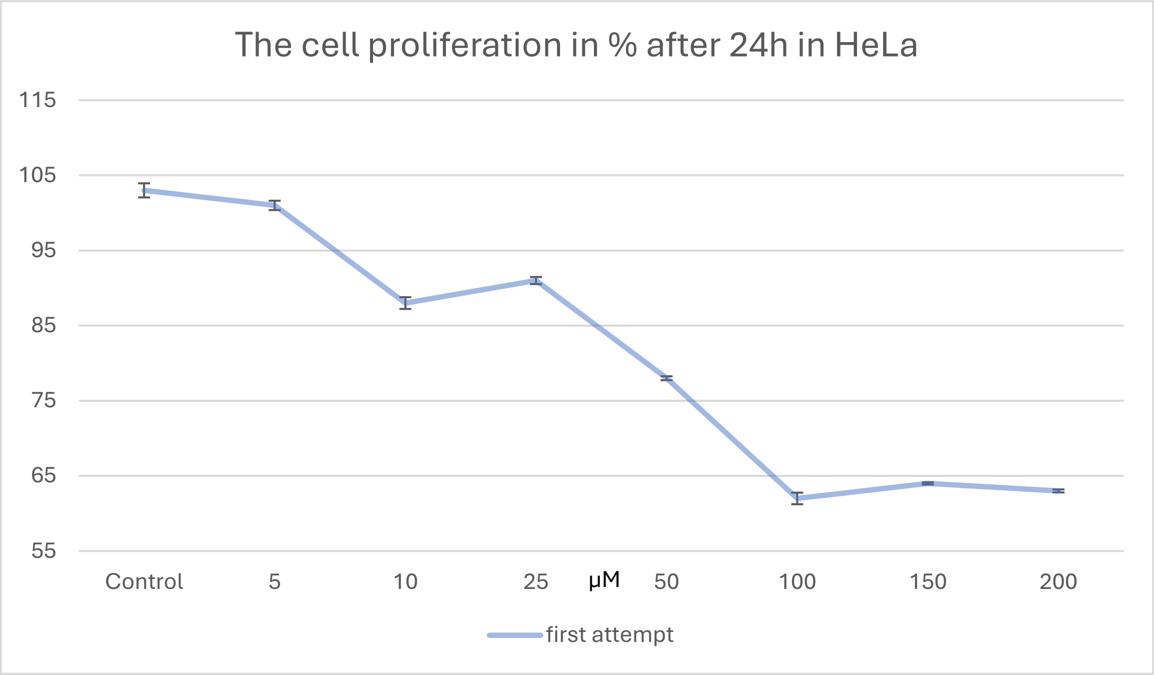

Figure S47. The cell proliferation in % after 24h exposition on the complex **1** in HeLa cell line

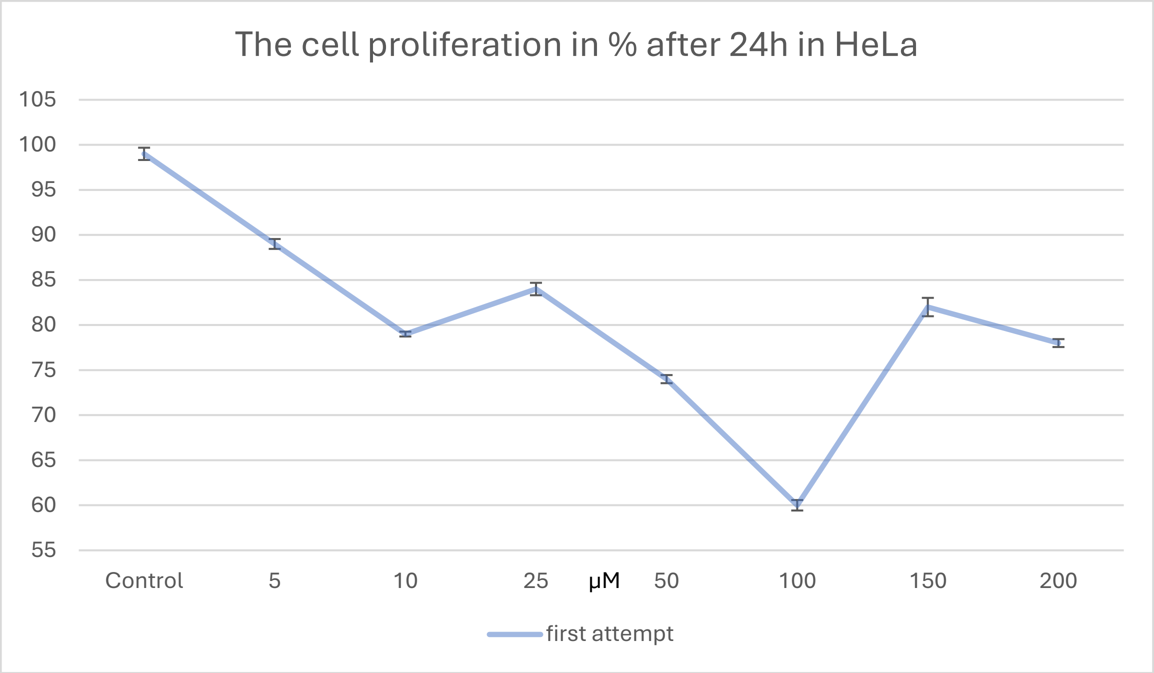

Figure S48. The cell proliferation in % after 24h exposition on the complex **2** in HeLa cell line

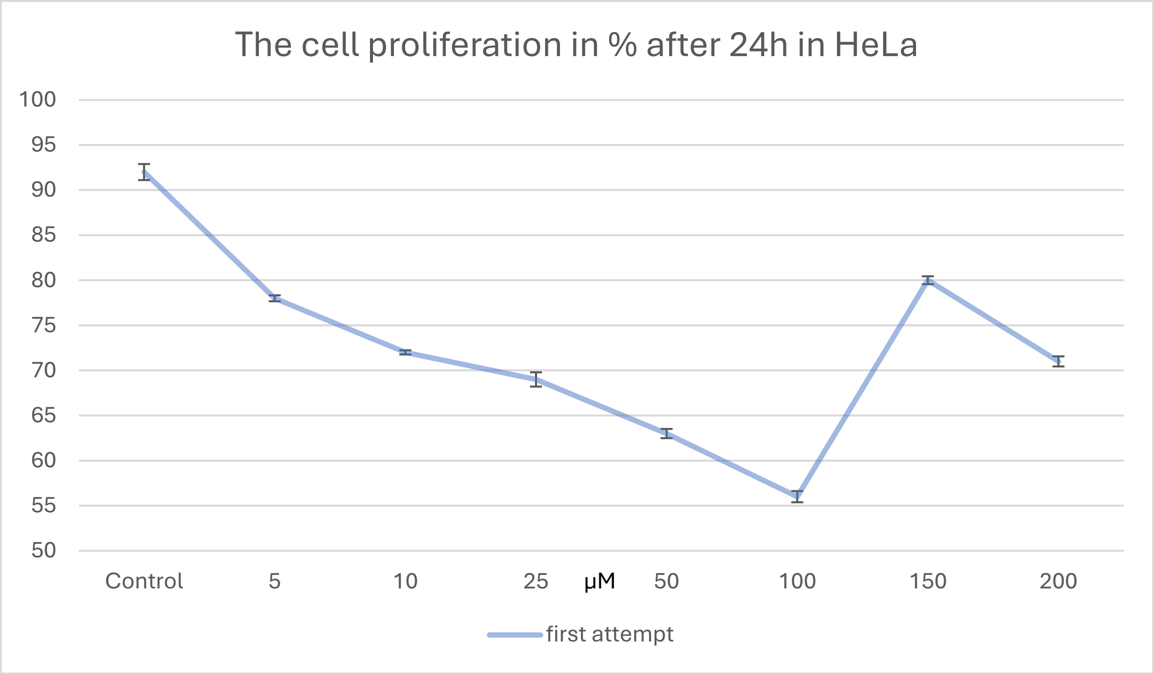

Figure S49. The cell proliferation in % after 24h exposition on the complex **3** in HeLa cell line

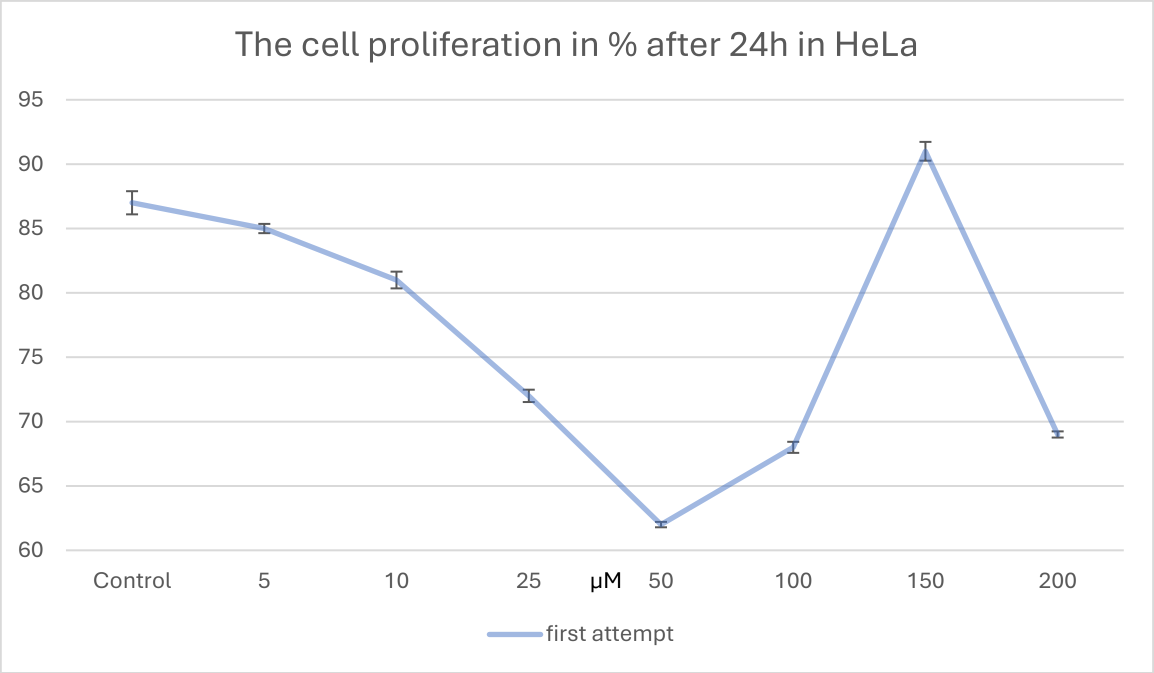

Figure S50. The cell proliferation in % after 24h exposition on the complex **4** in HeLa cell line

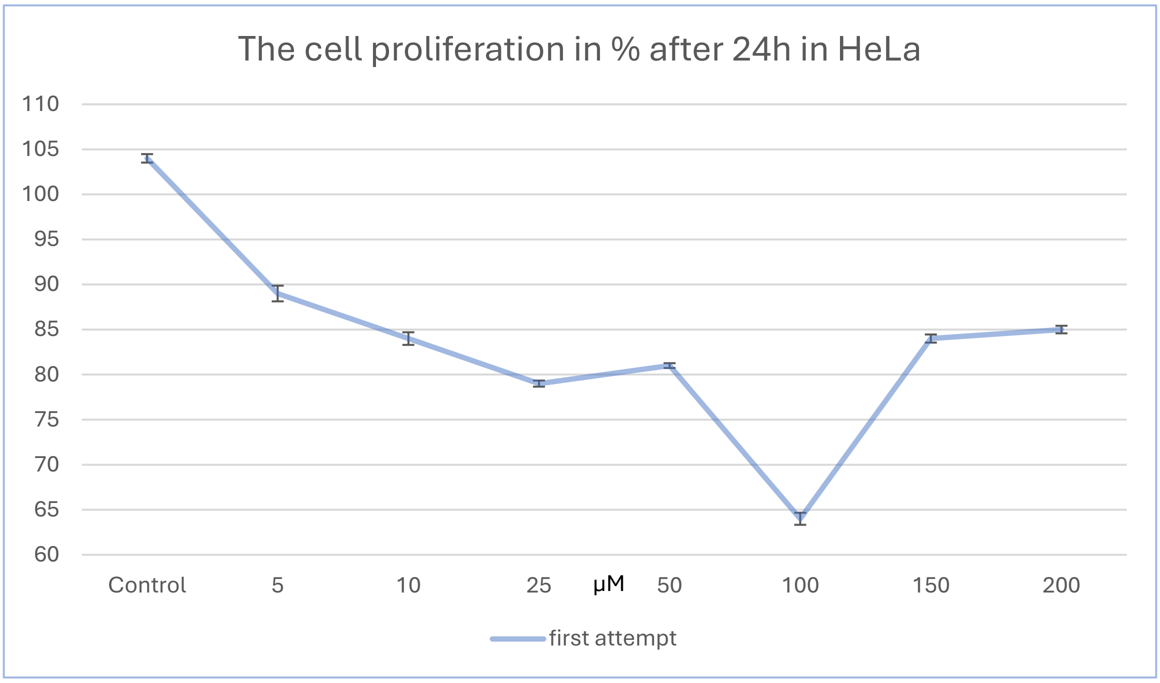

Figure S51. The cell proliferation in % after 24h exposition on the complex **5** in HeLa cell line

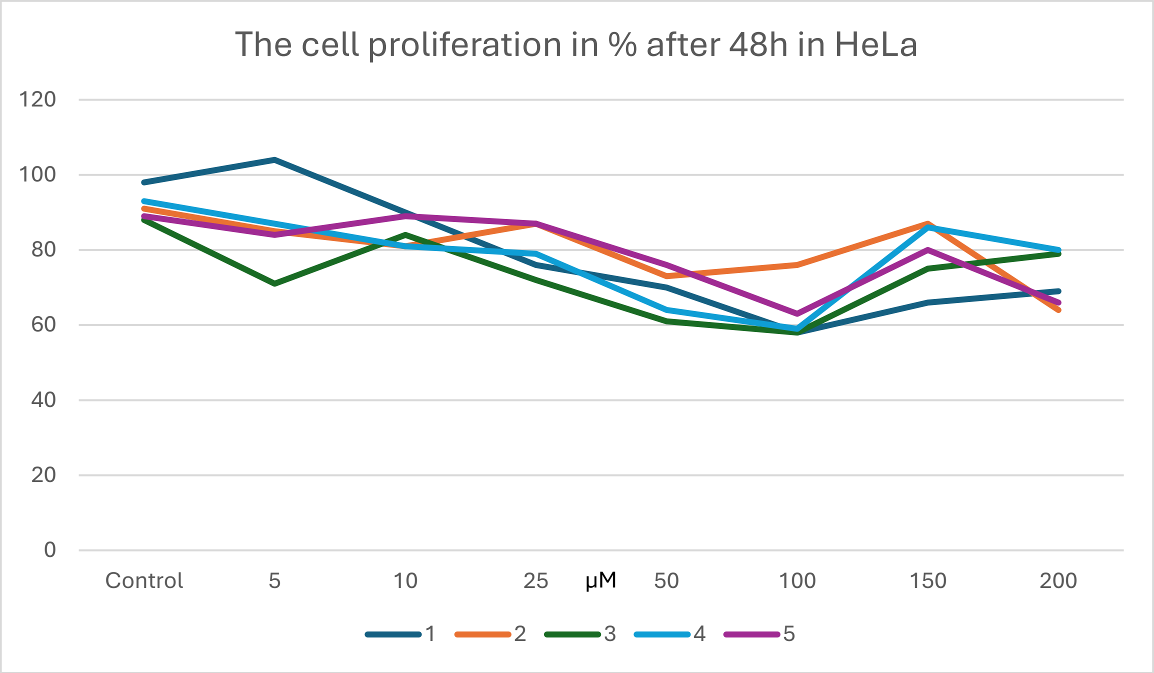

Figure S52. The cell proliferation in % after 48h exposition on the all complexes **1** – **5** in HeLa cell line

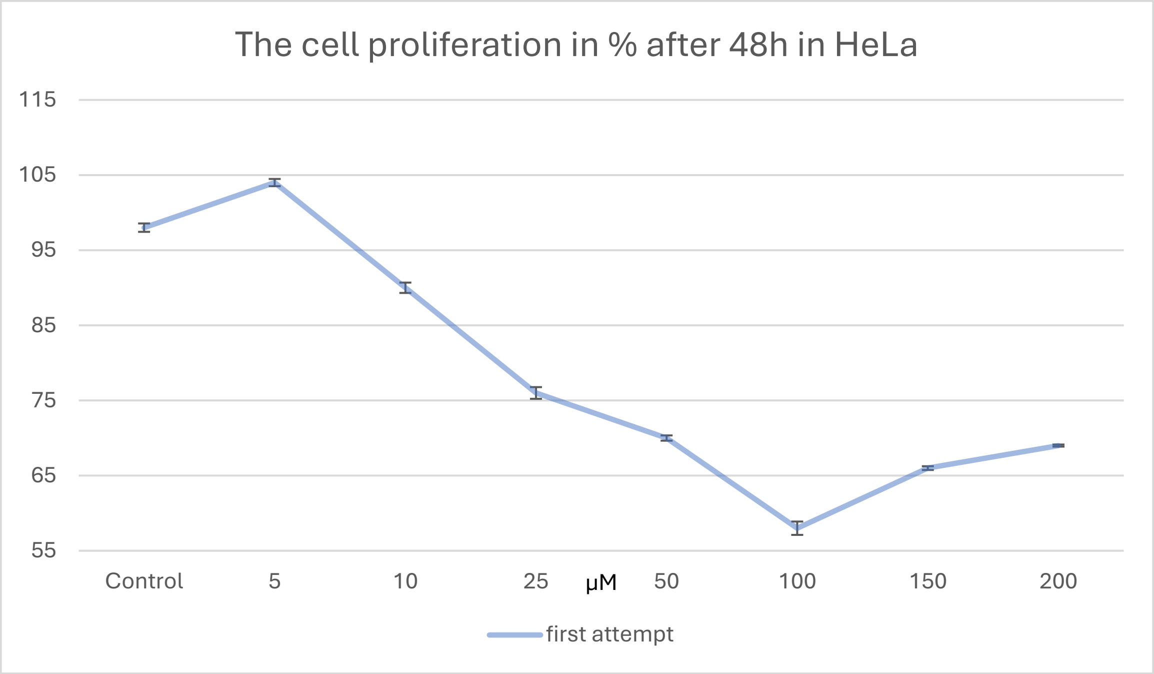

Figure S53. The cell proliferation in % after 48h exposition on the complex **1** in HeLa cell line

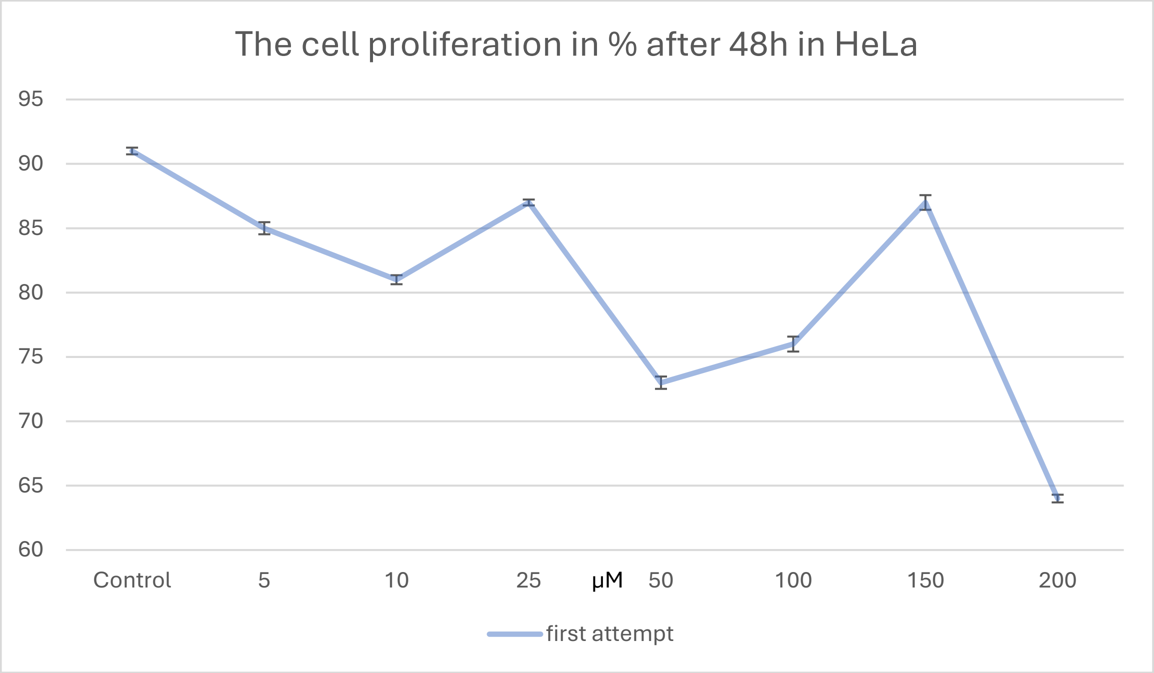

Figure S54. The cell proliferation in % after 48h exposition on the complex **2** in HeLa cell line

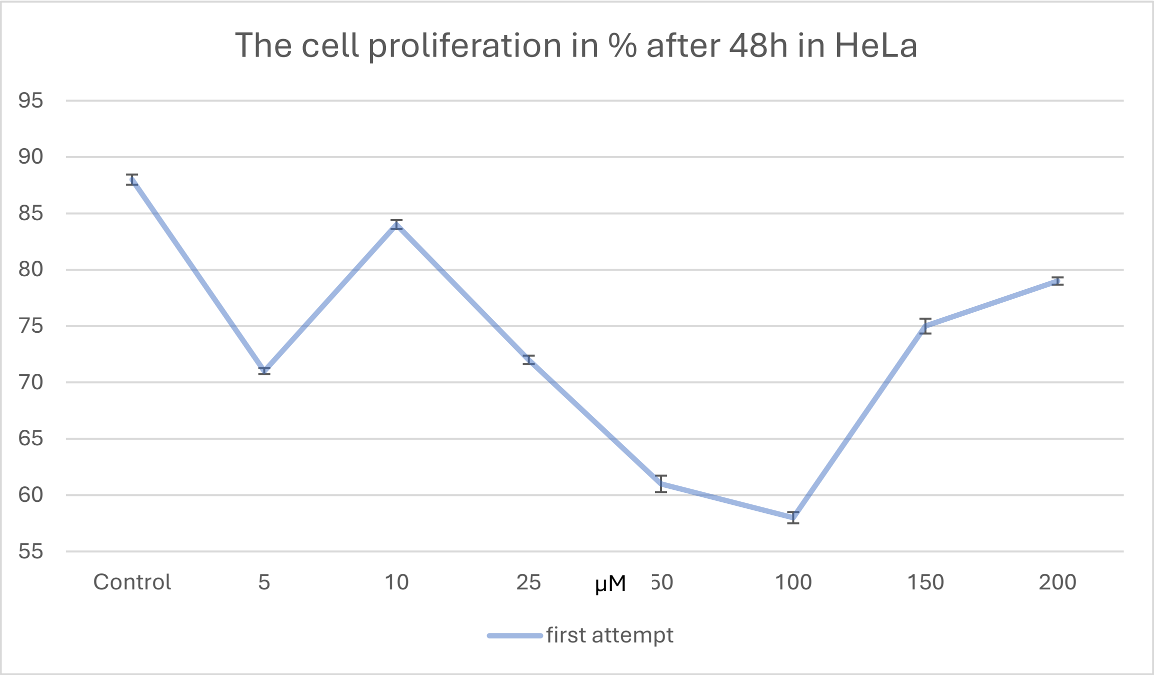

Figure S55. The cell proliferation in % after 48h exposition on the complex **3** in HeLa cell line

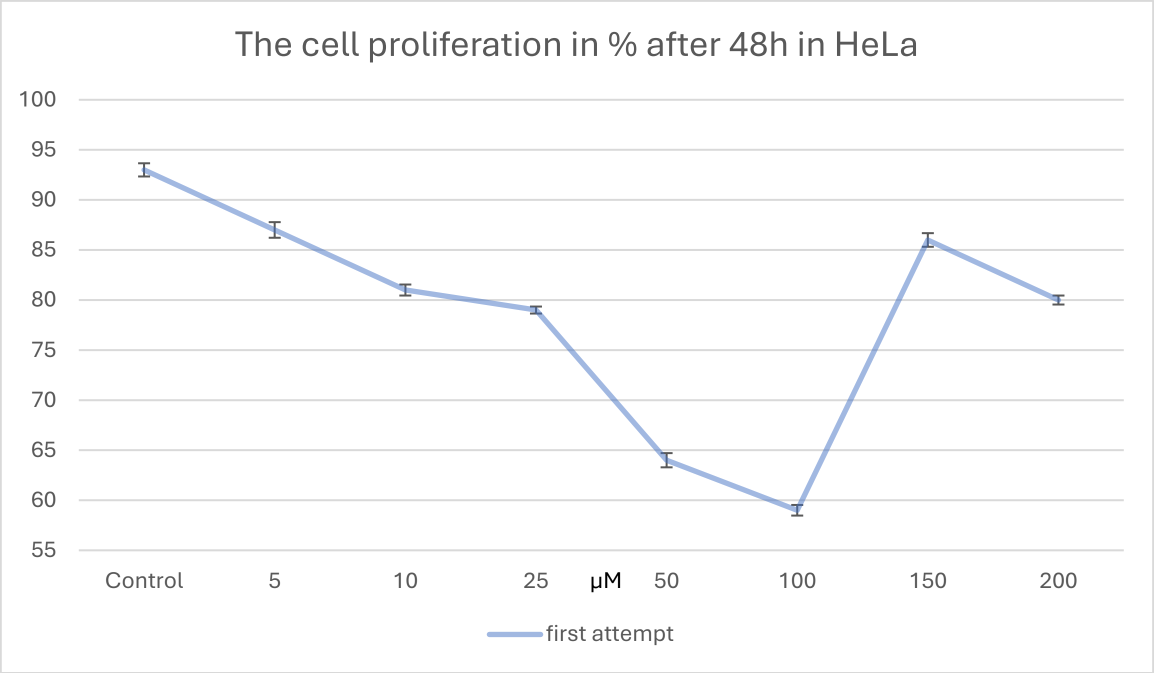

Figure S56. The cell proliferation in % after 48h exposition on the complex **4** in HeLa cell line

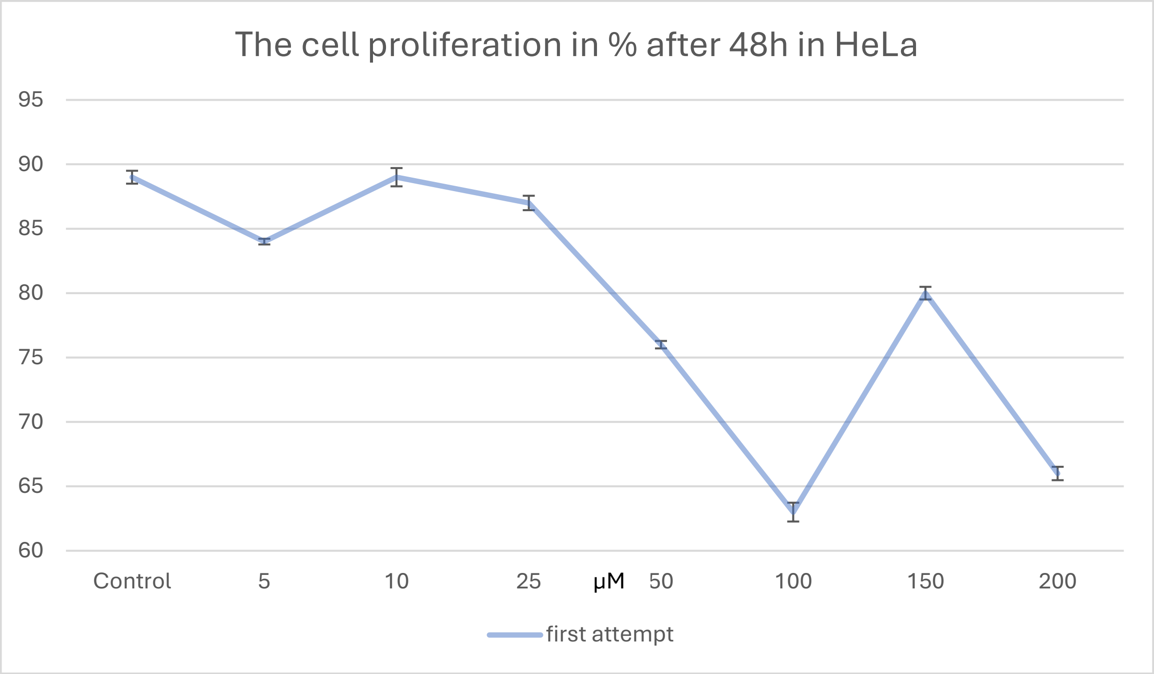

Figure S57. The cell proliferation in % after 48h exposition on the complex **5** in HeLa cell line
